# Supplementary material for: Organocatalyst-mediated five-pot synthesis of (–)-quinine
Source: Nat Commun. 2022 Dec 7;13:7503. doi: 10.1038/s41467-022-34916-z (PMC9729207; doi:10.1038/s41467-022-34916-z)
Supplement: Supplementary file 2 — Supplementary Information [file 41467_2022_34916_MOESM2_ESM.pdf]

Supplementary Information

Organocatalyst-mediated five-pot synthesis of (–)-quinine

Takahiro Terunuma[1], Yujiro, Hayashi[1]*

[1] Department of Chemistry, Graduate School of Science, Tohoku University 6-3 Aramaki Aza-Aoba, Aoba-ku, Sendai, Miyagi 980-8579, Japan

Experimental procedures and Characterization data

Table of Contents

1. Supplementary Methods ········ S2
   1.1 General information ········ S2
   1.2 Experimental section ········ S3
      1.2.1 Synthesis of alcohol **S3** ········ S3
      1.2.2 Synthesis of aldehyde **5** ········ S3
      1.2.3 Synthesis of nitroalkene **6** ········ S4
      1.2.4 Synthesis of imine precursor **9** ········ S5
      1.2.5 Synthesis of **S12** to determine *ee* ········ S5
      1.2.6 Synthesis of tetrahydropyridine **10** ········ S6
      1.2.7 Synthesis of piperidine **15** ········ S7
      1.2.8 Synthesis of aldehyde **18** ········ S8
      1.2.9 Synthesis of 2,4-dibromo-6-methoxyquinoline (**S19**) ········ S8
      1.2.10 Synthesis of alcohol **20** and **epi-20** ········ S9
      1.2.11 Synthesis of alcohol **20** from **epi-20** ········ S10
      1.2.12 Synthesis of (–)-quinine (**1**) ········ S11
      1.2.13 Synthesis of Synthesis of Br(C2')-quinine **25** ········ S12
      1.2.14 Synthesis of **26** ········ S13
      1.2.15 Synthesis of **27** ········ S13
      1.2.16 Synthesis of **28** ········ S14
   1.3 Copies of spectra ········ S15
2. Supplementary References ········ S74

1. Supplementary Methods

1.1 General information

General Remarks: All reactions were carried out under argon atmosphere and monitored by thin-layer chromatography using Merck 60 F254 precoated silica gel plates (0.25 mm thickness). Specific optical rotations were measured using a JASCO P-2200 polarimeter. FT-IR spectra were recorded on a JASCO FT/IR-4600HC1 spectrometer. ^{1}H and ^{13}C NMR spectra were recorded on an Agilent-400 MR (400 MHz for ^{1}H NMR, 100 M Hz for ^{13}C NMR) instrument. Data for ^{1}H NMR are reported as chemical shift (δ ppm), integration multiplicity (s = singlet, d = doublet, t = triplet, q = quartet, septet = sep, dd = doublet of doublets, ddd = doublet of doublet of doublets, ddt = doublet of doublet of triplets, dt = doublet of triplets, dq = doublet of quartets, m = multiplet, brs = broad singlet, brd = broad doublet, brt = broad triplet), coupling constant (Hz), Data for ^{13}C NMR are reported as chemical shift. High resolution ESI-TOF mass spectra were measured by Themo Orbi-trap LTQ XL instrument. HPLC analysis was performed on a HITACHI Elite LaChrom Series HPLC, UV detection monitored at appropriate wavelength respectively, using CHIRALPACK® IB (0.46 cm × 25 cm). Melting point was measured using Yanaco MP-J3. Flash chromatography was performed using silica gel 60N (spherical) or silica gel 60N (spherical) NH_2 of Kanto Chemical Co. Int., Tokyo, Japan. All reagents were purchased from commercial sources (Aldrich, FUJIFILM Wako chemicals, Kanto Chemical, TCI). **S12** (product code: QB-0349) was purchased from FUJIFILM Wako chemicals.

1.2 Experimental section

1.2.1 Synthesis of alcohol S3

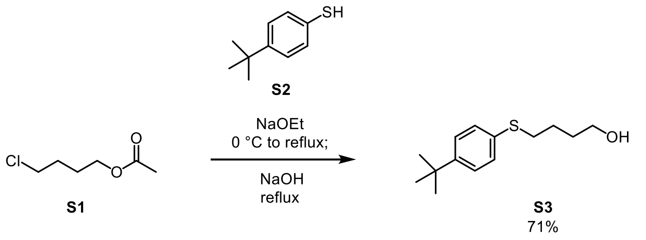

Using 4-^{t}Butylbenzenethiol (**S2**), **S3** was prepared in accordance with a literature procedure[1]. 4-^{t}Butylbenzenethiol (**S2**) (13.0 mL, 75.3 mmol) was added dropwise to a vigorously stirred solution of NaOEt (5.13 g, 75.3 mmol) in EtOH (380 mL) at 0 °C. The mixture was then stirred for further 15 minutes, and then 4-chlorobutyl acetate (**S1**) (10.4 mL, 75.3 mmol) was added dropwise over 30 minutes. The resulting solution was then heated under reflux for 6 h and stirred at room temperature for further 10 h. NaOH pellets (3.01 g, 75.3 mmol) were added, and the suspension was heated under reflux for 2 h. The reaction mixture was cooled to room temperature and poured into water. The solution was extracted with Et_2O three times, and the combined organic layer was washed with 1M NaOH solution twice and water twice, dried over $MgSO_4$. Removal of the solvents in vacuo gave crude product. The residue was purified by flash chromatography on silica gel (^{n}Hexane:AcOEt = 12:1) gave **S3** (12.7 g, 71%) as a colorless liquid. R_f = 0.28 (^{n}Hexane:EtOAc = 3:1, color reagent: Cerium molybdate stain)

^{1}H NMR (400 MHz, $CDCl_3$) δ 7.34-7.26 (m, 4H), 3.70-3.62 (m, 2H), 2.97-2.89 (m, 2H), 1.77-1.66 (m, 4H), 1.30 (s, 9H); **^{13}C NMR** (100 MHz, $CDCl_3$) δ 149.3, 133.0, 129.5, 126.0, 62.5, 34.5, 34.0, 31.8, 31.4, 25.7; **IR** (neat) ν 3320, 2959, 2867, 1497, 1362, 1268, 1120, 1012, 820, 727, 550, 508 cm^{-1}; **HRMS** (ESI): *m/z*: calculated for $C_{14}H_{22}OSNa^+$ $[M+Na]^+$: 261.1284, found: 261.1299.

1.2.2 Synthesis of aldehyde 5

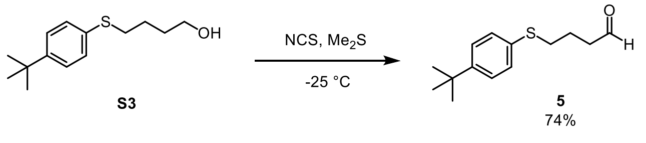

Using **S3**, **5** was prepared in accordance with a literature procedure[1]. Dimethyl sulfide (8.06 mL, 10.9 mmol) was added dropwise to a stirred solution of *N*-chlorosuccinimide (11.6 g, 8.72 mmol) in toluene (230 mL) at 0 °C. The resulting suspension was then cooled to -25 °C and **S3** (13.0 g, 54.5 mmol) was added dropwise to the stirred solution. Stirring was continued at -25 °C for a further 2 h and triethylamine (7.60 mL, 54.5 mmol) in toluene (2.0 ml) was added dropwise. After diluting with Et_2O, the organic layer was washed with 1% aqueous HCl solution twice and water twice, then dried over $MgSO_4$. Removal of the solvents under reduced pressure gave a crude product. The residue was purified

by flash chromatography on silica gel (^{n}Hexane:EtOAc = 15:1) gave **5** (9.62 g, 74%) as a colorless liquid. R_f = 0.40 (^{n}Hexane:EtOAc = 4:1, color reagent: Cerium molybdate stain)

^{1}H NMR (400 MHz, $CDCl_3$) δ 9.78 (t, J = 1.2 Hz, 1H) , 7.35-7.27 (m, 4H), 2.93 (t, J = 7.2 Hz, 2H), 2.62 (td, J = 7.2, 1.2 Hz, 2H), 1.96 (sep, J = 7.2 Hz, 2H), 1.31 (s, 9H); **^{13}C NMR** (100 MHz, $CDCl_3$) δ 201.7, 149.7, 132.2, 129.9, 126.2, 42.8, 34.6, 33.6, 31.4, 21.8; **IR** (neat) ν 2961, 2868, 1725, 1490, 1461, 1393, 1363, 1269, 1121, 1012, 822, 549 cm^{-1}; **HRMS** (ESI): m/z: calculated for $C_{14}H_{20}OSNa^+$ $[M+Na]^+$: 259.1127, found: 259.1135.

1.2.3 Synthesis of nitroalkene 6

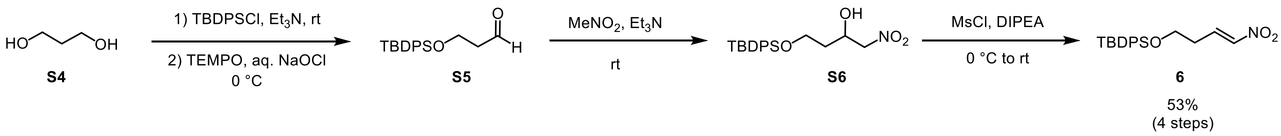

To a stirred solution of TBDPSCl (30.0 mL, 100 mmol) in CH_2Cl_2 (260 mL), 1,3-propanediol (**S4**) (20.0 mL, 300 mmol) and Et_3N (20.0 mL, 150 mmol) were added at room temperature. After stirring for 3 h at the same temperature, the reaction was quenched with water (100 mL). The organic layer was concentrated under reduced pressure. The residue was dissolved in hexane (300 mL) and washed with water (200 mL). The organic layer was concentrated under reduced pressure. To a stirred solution of crude material, TEMPO (310 mg, 2.00 mmol), KBr (1.20 g, 10.0 mmol) in $CHCl_3$ (200 mL) and saturated aqueous $NaHCO_3$ (120 mL), 6% NaOCl aqueous solution (60.0 mL, 110 mmol) was added dropwise at 0 ℃. The resulting mixture was stirred at the same temperature for 10 min. The organic layer was extracted with CH_2Cl_2 three times, and the combined organic layer was dried over Na_2SO_4, filtered, and concentrated under reduced pressure to afford crude aldehyde **S5.** To a stirred solution of crude material in nitromethane (26.0 mL, 500 mmol) was added triethylamine (1.39 mL, 10.0 mmol) dropwise over 5 minutes at room temperature. The solution was stirred for 16 h. Solvent was evaporated in vacuo and the crude **S6** was dissolved in CH_2Cl_2 (240 mL), MsCl (11.7 mL, 150 mmol) and ^{i}Pr$_2$EtN (53.2 mL, 300 mmol) were added to the mixture at 0 ℃. The mixture was allowed to warm to room temperature and stirred for 15 minutes. The reaction was quenched with water and diluted with CH_2Cl_2. The organic phase was washed with 2 M HCl solution, brine, dried over Na_2SO_4 and concentrated in vacuo. The residue was purified by flash column chromatography (^{n}Hexane:EtOAc = 25:1) to give **6** (18.8 g, 53%) as a yellow oil. R_f = 0.53 (^{n}Hexane:EtOAc = 4:1, color reagent: Cerium molybdate stain)

^{1}H NMR (400 MHz, $CDCl_3$) δ 7.67-7.61 (m, 4H), 7.50-7.36 (m, 6H), 7.30 (dt, J = 13.6, 7.2 Hz, 1H), 7.03 (dt, J =13.6, 1.2 Hz, 1H), 3.82 (t, J = 6.0 Hz, 2H), 2.47 (tdd, J = 7.2, 6.0, 1.2 Hz, 2H), 1.06 (s, 9H); **^{13}C NMR** (100 MHz, $CDCl_3$) δ 140.8, 139.9, 135.6, 133.2, 130.0, 127.9, 61.6, 31.7, 26.9, 19.2; **IR** (neat) ν 2931, 2858, 1525, 1472, 1428, 1352, 1112, 972, 822, 740, 702, 614 cm^{-1}; **HRMS** (ESI): m/z: calculated for $C_{20}H_{25}NO_3SiNa^+$ $[M+Na]^+$: 378.1496, found 378.1492. **mp**: 33.5-35.0 ℃.

1.2.4 Synthesis of imine precursor 9

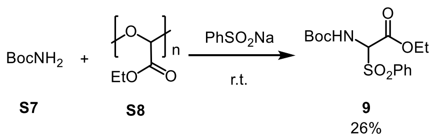

Using sodium benzenesulfinate dihydrate, **9** was prepared in accordance with a literature procedure[2]. ^{t}Butyl carbamate (**S7**) (11.7 g, 100 mmol), ethyl glyoxylate (**S8**) (50% in toluene, 43.0 g, 200 mmol) and sodium benzenesulfinate dihydrate (60.1 g, 300 mmol) were dissolved in formic acid (50% in H_2O, 100 mL) and stirred for 1.5 days at room temperature. The reaction mixture was poured into ice water (20 mL) and the generated white solid was washed with water and dried in vacuo to give **9** (8.92 g, 26%) as a white solid. R_f = 0.46 (^{n}Hexane:EtOAc = 2:1, color reagent: anisaldehyde)

^{1}H NMR (400 MHz, $CDCl_3$) δ 7.95 (d, J = 7.2 Hz, 2H), 7.68 (t, J = 7.2 Hz, 1H), 7.57 (d, J = 7.2 Hz, 2H), 5.80 (d, J = 10.0 Hz, 1H), 5.60 (d, J = 10.0 Hz, 1H), 4.33 (q, J = 7.2Hz, 2H), 1.34 (t, J = 7.2 Hz, 3H), 1.31 (s, 9H); **^{13}C NMR** (100 MHz, $CDCl_3$) δ 163.3, 153.4, 136.8, 134.5, 129.6, 129.2, 81.6, 73.5, 63.5, 28.0, 14.0; **IR** (neat) ν 3350, 2980, 1719, 1507, 1448, 1394, 1370, 1328, 1255, 1148, 1082, 1055, 1026, 688, 538 cm^{-1}; **HRMS** (ESI):*m/z*: calculated for $C_{15}H_{22}NO_6S^+$ $[M+H]^+$: 344.1162, found 344.1165. **mp**: 108-110 ℃.

1.2.5 Synthesis of S12 to determine *ee*

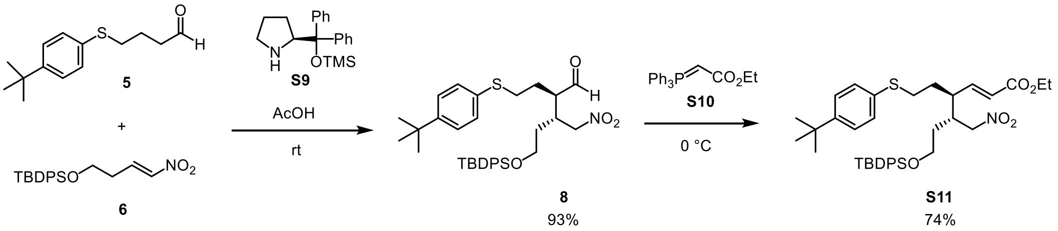

To a mixture of nitroalkene **6** (1.07 g, 3.00 mmol) and aldehyde **5** (0.850 g, 3.60 mmol) in toluene (3.0 mL), catalyst **S9** (94.8 mg, 0.300 mmol) and acetic acid (17.1 μL, 0.300 mmol) were added at room temperature. After stirring for 12 h, solvent was evaporated in vacuo. The residue was purified by flash chromatography on silica gel (^{n}Hexane:EtOAc = 20:1) gave **8** (1.7 g, 93%) as a yellow oil. R_f = 0.40 (^{n}Hexane:EtOAc = 4:1, color reagent: anisaldehyde), $[\alpha]_D^{19}$ +8.9 (c 1.20, $CHCl_3$).

8: **^{1}H NMR** (400 MHz, $CDCl_3$) δ 9.70 (s, 1H), 7.73-7.64 (m, 4 H), 7.52-7.34 (m, 6H), 7.39-7.29 (m, 4H), 4.53 (dd, J = 13.2, 6.0 Hz, 1H), 4.32 (dd, J = 13.2, 7.6 Hz, 1H), 3.78-3.65 (m, 2H), 3.09 (ddd, J = 13.6, 8.0, 6.0 Hz, 2 H), 2.93-2.82 (m, 2H), 2.16-2.03 (m, 1H), 1.69-1.54 (m, 2H), 1.54-1.44 (m, 1H), 1.35 (s, 9H), 1.10 (s, 9H); **^{13}C NMR** (100 MHz, $CDCl_3$) δ 202.1, 149.8, 135.6, 135.6, 133.1, 131.6, 130.0, 130.0, 129.9, 129.6, 127.9, 127.9, 126.2, 77.5, 76.5, 61.0, 51.2, 34.5, 33.9, 32.0, 31.3, 26.9, 24.4, 19.2; **IR** (neat) ν 2960, 2859, 1724, 1553, 1488, 1472, 1428, 1382, 1362, 1268, 1112, 1011, 823, 739, 703, 614, 505 cm^{-1}; **HRMS** (ESI): *m/z*: calculated for $C_{34}H_{45}O_4SSiNa^+$ $[M+Na]^+$: 614.2731, found: 614.2735.

To a mixture of aldehyde **8** (31.9 mg, 54.0 μmol) in toluene (540 μL), ylide **S10** (55.7 mg, 160 μmol) was added at 0 °C. After stirring for 5 h, excess solvent was evaporated in vacuo. The residue was purified by flash chromatography on silica gel (^{n}Hexane:EtOAc = 20:1) gave **S11** (27.3 mg, 74%) as a yellow oil. R_f = 0.40 (Toluene:EtOAc = 10:1, color reagent: anisaldehyde), $[\alpha]_D^{19}$ +8.9 (c 1.20, $CHCl_3$). The enantiomeric ratio was determined by HPLC using CHIRALPACK® IB (^{n}Hexane: ^{i}PrOH = 99:1; flow rate 1.0 mL/min, major isomer t_R = 12.1 min, minor isomer t_R = 13.3 min) (98% *ee*).

S11: ^{1}**H NMR** (400 MHz, $CDCl_3$) δ 7.67-7.58 (m, 4H), 7.47-7.39 (m, 6H), 7.34-7.19 (m, 4H), 6.63 (dd, *J* = 15.2, 8.8 Hz, 1H), 5.81 (d, *J* =15.2 Hz, 1H), 4.33 (d, *J* = 4.8 Hz, 2H), 4.19 (q, *J* = 7.2 Hz, 2H), 3.68 (t, *J* = 5.6 Hz, 2H), 2.94-2.82 (m, 1H), 2.73-2.63 (m, 1H), 2.62-2.52 (m, 2H), 1.84-1.71 (m, 1H), 1.70-1.59 (m, 1H), 1.54-1.42 (m, 1H), 1.33-1.23 (m, 12H), 1.03 (s, 9H), 0.92-0.78 (m, 1H); ^{13}**C NMR** (100 MHz, $CDCl_3$) δ 165.9, 149.9, 146.9, 135.7, 133.3, 133.3, 132.0, 130.0, 128.0, 126.2, 125.0, 76.9, 61.1, 60.8, 42.2, 38.3, 34.6, 32.1, 31.7, 31.4, 30.4, 27.0, 19.2, 14.4; **IR** (neat) ν 2959, 1719, 1654, 1553, 1472, 1428, 1368, 1428, 1368, 1267, 1185, 1112, 823, 738, 704, 661, 648, 613, 559 cm^{-1}; **HRMS** (ESI): *m/z*: calculated for $C_{38}H_{51}NO_5SSiNa^+$ $[M+Na]^+$: 684.3149, found: 684.3155.

1.2.6 Synthesis of tetrahydropyridine 10

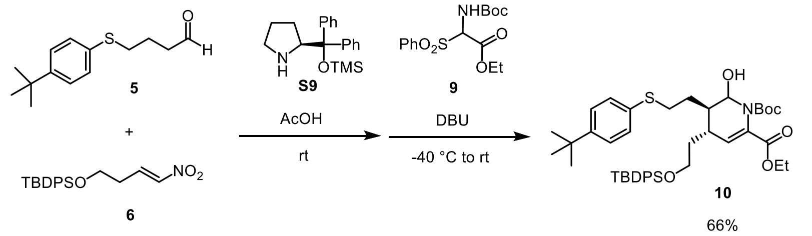

To a mixture of nitroalkene **6** (10.5 g, 29.5 mmol) and aldehyde **5** (8.38 g, 35.4 mmol) in toluene (29.5 mL), catalyst **S9** (961 mg, 2.95 mmol) and AcOH (169 μL, 2.95 mmol) were added at room temperature. The reaction mixture was stirred for 15 h. Toluene (266 mL) and **9** (20.3 g, 59.1 mmol) were added to the crude mixture. DBU (13.2 mL, 88.6 mmol) was added to the reaction mixture at -40 °C. After stirring for 5 h, The reaction mixture was warmed to room temperature and additional DBU (6.6 mL, 29.5 mmol) was added. After stirring for 2 h, the reaction was quenched with water. The resulting mixture was extracted with EtOAc three times. The combined organic layer was washed with brine, dried over Na_2SO_4, filtered, and concentrated in vacuo to give the crude products. Purification by silica gel column chromatography (^{n}Hexane:EtOAc = 8:1) afforded **10** (14.5 g, 66%, dr = 1:1) as a pale-yellow amorphous solid. R_f = 0.32 (^{n}Hexane:EtOAc = 4:1, color reagent: ninhydrin), $[\alpha]_D^{24}$ +31.8 (c 0.86, $CHCl_3$)

^{1}**H NMR** (400 MHz, $CDCl_3$) δ 7.69-7.61(m, 4H), 7.45-7.33(m, 6H), 7.31-7.22 (m, 4H), 6.10 (d, *J* = 3.6 Hz, 0.5H), 5.97 (d, *J* = 3.2 Hz, 0.5H), 5.82 (s, 0.5H), 5.68 (s, 0.5H), 4.33-4.09 (m, 2H), 3.79-3.64 (m, 2H), 3.09 (ddd, *J* = 12.4, 9.2, 5.6 Hz, 1H), 2.97-2.89 (m, 1H), 2.89-2.78 (m, 1H), 2.39-2.30 (m, 1H), 2.16-2.10 (m, 0.5H), 1.83-1.76 (m, 3.5H), 1.60-1.48 (m, 1H), 1.48-1.38 (m, 9H), 1.35-1.17 (m,

12H), 1.80-1.00 (m, 9H); **^{13}C NMR** (100 MHz, $CDCl_3$) δ 165.5, 165.3, 149.6, 149.4, 135.7, 135.7, 135.7, 135.7, 135.6, 133.7, 133.7, 133.7, 132.8, 132.5, 130.0, 129.8, 129.4, 127.8, 127.8, 126.1, 126.1, 126.1, 126.1, 124.8, 82.2, 82.1, 78.8, 75.9, 61.7, 61.3, 61.3, 61.2, 53.6, 42.3, 42.2, 37.3, 34.5, 34.5, 33.4, 32.9, 32.0, 31.9, 31.6, 31.4, 31.4, 29.3, 28.3, 28.2, 28.1, 27.0, 26.8, 19.3, 19.2, 14.3; **IR** (neat) ν 2964, 2253, 1709, 1473, 1428, 1370, 1261, 1162, 1112, 906, 823, 731, 650, 615 cm^{-1}; **HRMS** (ESI):*m/z*: calculated for $C_{43}H_{59}NO_6SiNa^+$ [M+Na]$^+$: 768.3725, found 768.3724.

1.2.7 Synthesis of piperidine 15

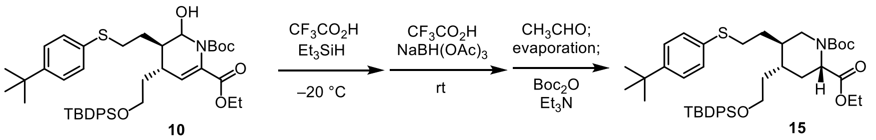

Triethylsilane (25.9 mL, 163 mmol) and TFA (12.5 mL, 163 mmol) were added to the solution of **10** (8.10 mg, 10.9 mmol) in CH_2Cl_2 (109 mL) at -20 °C. After stirring for 15 h, TFA (35.9 mL) was added to the reaction mixture and stirred for 30 min at room temperature. $NaBH(OAc)_3$ (4.60 g, 21.7 mmol) was added to the reaction mixture and stirred for 30 min. Acetaldehyde (1.81 mL, 32.6 mmol) was added and stirred for 15 min at room temperature. The solvent was removed under reduced pressure. 1,4-dioxane (54.5 mL), water (54.5 mL), triethylamine (15.1 mL, 109 mmol) and Boc_2O (22.4 mL, 97.7 mmol) were added to the crude mixture. The reaction mixture was stirred for 15 min and the reaction was quenched with 10% aqueous citric acid. The organic phase was extracted with CH_2Cl_2 three times, dried over Na_2SO_4, and concentrated in vacuo after filtration. The residue was purified by column chromatography (^{n}Hexane:EtOAc =15:1) to give **15** (6.20 g, 78%) as a pale yellow oil. R_f = 0.39 (^{n}Hexane:EtOAc = 6:1, color reagent: ninhydrin), $[\alpha]_D{}^{28}$ +8.7 (c 0.75, $CHCl_3$).

^{1}H NMR (400 MHz, $CDCl_3$) δ 7.67-7.61 (m, 4H), 7. 46-7.35 (m, 6H), 7.32-7.24 (m, 4H), 4.19-4.07 (m, 2H), 3.78-3.60 (m, 3H), 3.21 (brd, *J*= 13.6 Hz, 1H), 3.02 (brs, 1H), 2.84 (ddd, *J* = 15.2, 8.4, 5.2 Hz, 1H), 2.04-1.95 (m, 1 H), 1.79-1.69 (m, 2H), 1.68-1.46 (m, 6H), 1.43 (s, 9H), 1.29 (s, 9H), 1.24 (t, 7.2 Hz, 3H), 1.04 (s, 9H); **^{13}C NMR** (100 MHz, $CDCl_3$) δ 172.9, 155.8, 149.4, 135.7, 135.7, 135.7, 133.8, 133.8, 133.0, 129.8, 129.8, 129.6, 127.8, 126.1, 80.2, 61.7. 61.0, 38.0, 36.7, 34.5, 33.3, 32.1, 31.4, 28.5, 27.0, 19.3, 19.3, 19.3, 14.3, 6.7, 5.9; **IR** (neat) ν 2959, 2858, 1738, 1428, 1391, 1270, 1189, 1112, 1029, 915, 822, 735, 703, 614, 568, 555, 503 cm^{-1}; **HRMS** (ESI):*m/z*: calculated for $C_{43}H_{61}NO_5SSiNa^+$ [M+Na]$^+$: 754.3932, found 754.3933.

1.2.8 Synthesis of aldehyde 18

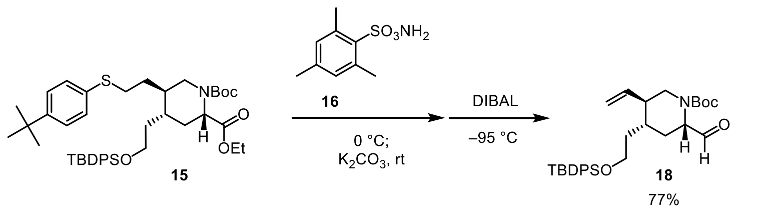

To a mixture of **15** (4.50 g, 6.15 mmol) in CH_2Cl_2 (41.0 mL), **16** (1.98 g, 9.22 mmol) was added at 0 ℃. After stirring for 30 minutes, K_2CO_3 (2.55 g, 18.4 mmol) was added, and the reaction mixture was stirred for 13 h. DIBAL (30.7 mL, 1.0 M solution in toluene, 30.7 mmol) was slowly added to the reaction mixture at -95 ℃. After stirring for 30 minutes, the reaction was quenched with MeOH (30.7 mL) at -95 ℃. To the resulting mixture, saturated aqueous Rochelle's salt solution was added at room temperature. The aqueous layer was extracted with EtOAc three times. The combined organic layer was washed with brine, dried over Na_2SO_4, filtered, and concentrated in vacuo to give the crude products. Purification by silica gel column chromatography (^{n}Hexane: EtOAc = 15:1) afforded **18** (2.46 g, 77%) as a pale-yellow oil. R_f = 0.60 (^{n}Hexane: EtOAc = 4:1, color reagent: ninhydrin), $[\alpha]_D{}^{26}$ -16.6 (c 0.67, $CHCl_3$)

^{1}H NMR (400 MHz, $CDCl_3$) δ 9.42 (d, J = 2.4 Hz, 1H), 7.68-7.59 (m, 4H), 7.46-7.34 (m, 6H), 5.54 (ddd, J = 17.6, 10.0, 8.4 Hz, 1H), 5.15-5.06 (m, 2H), 3.80-3.62 (m, 3H), 3.55 (dq, J = 10.8, 2.8 Hz, 1H), 2.81 (dd, J = 12.0, 10.8 Hz, 1H), 1.97-1.81 (m, 3H), 1.47 (s, 9H), 1.31-1.09 (m, 3H), 1.04 (s, 9H); **^{13}C NMR** (100 MHz, $CDCl_3$) δ 196.6, 156.3, 138.6, 135.7, 135.7, 133.8, 133.8, 129.8, 129.8, 127.8, 127.8, 117.4, 81.7, 63.6, 61.3, 48.6, 45.5, 36.2, 34.9, 29.4, 28.3, 27.0, 19.3; **IR** (neat) ν 2931, 1734, 1687, 1367, 1248, 1166, 1111, 919, 823, 739, 703, 615, 524 cm^{-1}; **HRMS** (ESI):*m/z*: calculated for $C_{31}H_{43}NO_4SiNa^+$ $[M+Na]^+$: 544.2854, found 544.2847.

1.2.9 Synthesis of 2,4-dibromo-6-methoxyquinoline (19)

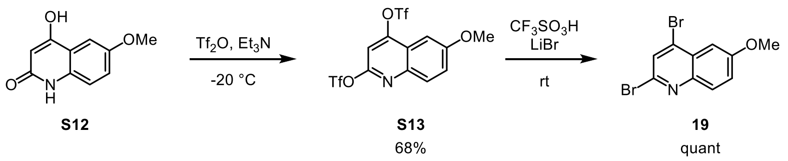

To a mixture of **S12** (1.18 g, 6.17 mmol) and Et_3N (3.16 mL, 22.7 mmol) in CH_2Cl_2 (19 mL), Tf_2O (2.45 mL, 14.5 mmol) was added dropwise at -20 ℃. After stirring for 30 minutes, the reaction was quenched with saturated aqueous $NaHCO_3$ solution. The aqueous layer was extracted with CH_2Cl_2 three times. The combined organic layer was dried over Na_2SO_4, filtered, and concentrated in vacuo to give the crude products. Purification by silica gel column chromatography (^{n}Hexane: EtOAc = 20:1) afforded **S13** (1.89 g, 67%) as a colorless amorphous solid. R_f = 0.43 (^{n}Hexane:EtOAc = 10:1, color reagent: Cerium molybdate stain).

S13: **^{1}H NMR** (400 MHz, $CDCl_3$) δ 8.01 (d, J = 9.2 Hz, 1H), 7.55 (dd, J = 9.2, 2.8 Hz, 1H), 7.28 (d, J = 2.8 Hz, 1H), 7.26 (s, 1H), 3.98 (s, 3H); **^{13}C NMR** (100 MHz, $CDCl_3$) δ 160.2, 154.0, 151.1, 143.0, 130.9, 126.0, 122.4, 120.4, 117.2, 105.6, 98.7, 56.0; **IR** (neat) ν 3445, 1608, 1508, 1440, 1423, 1327, 1212, 1129, 1111, 993, 885, 851, 835, 819, 760, 606, 586 cm^{-1}; **HRMS** (ESI):*m/z*: calculated for $C_{12}H_8F_6NO_7S_2^+$ $[M+H]^+$: 455.9641, found 455.9645.

Using **S13**, **19** was prepared in accordance with a literature procedure[3]. To a mixture of **S13** (1.16 g, 2.55 mmol) and LiBr (2.21 g, 25.5 mmol) in MTBE (6.4 mL), TfOH (249 μL, 2.80 mmol) was added over 10 minutes at room temperature. After stirring for 24 h, the reaction was quenched with saturated aqueous $NaHCO_3$ solution. The aqueous layer was extracted with CH_2Cl_2 three times. The combined organic layer was dried over Na_2SO_4, filtered, and concentrated in vacuo to give the crude products. Purification by silica gel column chromatography (^{n}Hexane: EtOAc = 20:1) afforded **19** (800 mg, 99%) as a colorless amorphous solid. R_f = 0.40 (^{n}Hexane: EtOAc = 10:1, color reagent: Cerium molybdate stain).

19: **^{1}H NMR** (400 MHz, $CDCl_3$) δ 7.90 (d, J = 9.2 Hz, 1H), 7.76 (s, 1H), 7.36 (dd, J = 9.2, 2.8 Hz, H), 7.31 (d, J = 2.8 Hz, 1H), 3.95 (s, 3H); **^{13}C NMR** (100 MHz, $CDCl_3$) δ 159.2, 144.7, 137.6, 133.6, 130.7, 128.8, 127.9, 124.1, 104.8, 55.9; **IR** (neat) ν 3709, 2978, 1619, 1557, 1494, 1447, 1398, 1278, 1232, 1092, 914, 856, 840 824, 504 cm^{-1}; **HRMS** (ESI):*m/z*: calculated for $C_{10}H_8Br_2NO^+$ $[M+H]^+$: 317.8947, found 317.8946.

1.2.10 Synthesis of alcohol 20 and epi-20

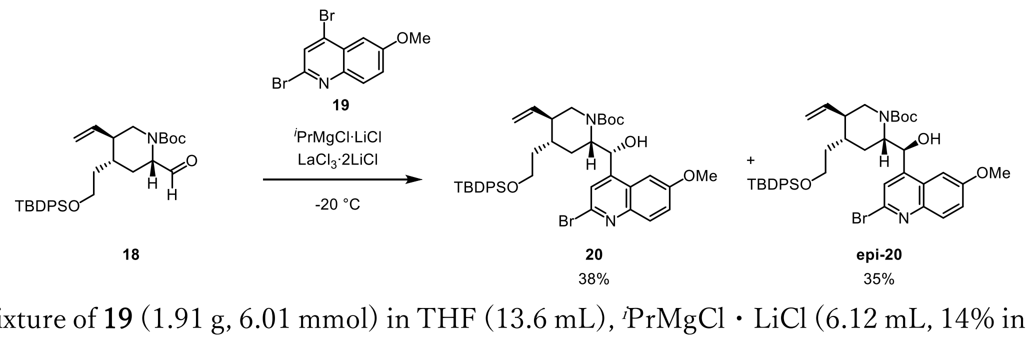

To a mixture of **19** (1.91 g, 6.01 mmol) in THF (13.6 mL), ^{i}PrMgCl・LiCl (6.12 mL, 14% in THF, 5.61 mmol) and $LaCl_3$・2LiCl (10.0 mL, 6.01 mmol, 0.6 M) were added at room temperature. After stirring for 2 h, aldehyde **18** (1.05 g, 2.00 mmol) in THF (6.8 mL) was added at -20 ℃. After stirring for 30 minutes, the reaction was quenched with saturated aqueous NH_4Cl solution. The aqueous layer was extracted with EtOAc three times. The combined organic layer was washed with brine, dried over Na_2SO_4, filtered, and concentrated in vacuo to give the crude products. Purification by silica gel column chromatography (^{n}Hexane: EtOAc = 15:1 to 10:1 to 5:1) afforded **20** (578 mg, 39%) as a pale-yellow amorphous solid and **epi-20** (530 mg, 36%) as a pale-yellow amorphous solid.

20: R_f = 0.43 (^{n}Hexane: EtOAc = 4:1, color reagent: ninhydrin), $[\alpha]_D^{29}$ -16.1 (c 0.60, $CHCl_3$); **^{1}H NMR** (400 MHz, $CDCl_3$) δ 7.83 (d, J = 9.2 Hz, 1H), 7.73 (d, J = 2.8 Hz, 1H), 7.70-7.65 (m, 1H), 7.64

(s, 1H), 7.55-7.46 (m, 4H), 7.42-7.22 (m, 6H), 5.75-5.56 (m, 2H), 5.01-4.90 (m, 2H), 4.15-4.03 (m, 1H), 4.00 (s, 3H), 3.66 (dd, *J* = 14.6, 2.0 Hz, 1H), 3.57-3.41 (m, 3H), 1.96-1.87 (m, 1H), 1.84 (brs, 1H), 1.73-1.56 (m, 2H), 1.50 (s, 9), 1.44-1.23 (m, 1H), 1.30-1.22 (m, 1H), 1.17 (dq, *J* = 13.3, 2.8 Hz, 1H) 0.87 (s, 9H); **^{13}C NMR** (100 MHz, $CDCl_3$) δ 158.2, 158.1, 149.0, 144.2, 140.9, 139.2, 135.6, 135.6, 135.4, 135.4, 134.0, 133.7, 133.6, 130.0, 129.5, 129.5, 127.6, 127.5, 127.5, 125.5, 122.8, 122.7, 114.3, 102.5, 80.0, 70.9, 61.8, 58.2, 56.0, 46.3, 45.8, 37.0, 33.9, 28.4, 26.9, 26.7, 18.9; **IR** (neat) ν 3390, 3072, 2930, 1675, 1621, 1557, 1507, 1472, 1419, 1365, 1289, 1239, 1153, 1111, 911, 832, 735, 703, 614 cm^{-1}; **HRMS** (ESI): *m/z*: calculated for $C_{41}H_{52}BrN_2O_5Si^+$ $[M+H]^+$: 759.2823, found 759.2813.

epi-20: R_f = 0.31 (EtOAc: ^{n}Hexane: = 1:4, color reagent: ninhydrin), $[\alpha]_D^{25}$ +28.3 (c 1.09, $CHCl_3$); **^{1}H NMR** (400 MHz, $CDCl_3$) δ 7.96 (d, *J* = 9.6 Hz, 1H), 7.60-7.46 (m, 6H), 7.43-7.29 (m, 7H), 5.67 (ddd, *J* = 16.8, 10.4, 8.8 Hz, 1H), 5.18 (brt, *J* = 5.2 Hz, 1H), 5.03-4.94 (m, 2H), 4.32-4.22 (m, 1H), 3.92 (s, 3H), 3.57-3.42 (m, 3H), 3.38-3.25 (m, 1H), 1.94-1.82 (m, 1H), 1.77-1.62 (m, 3H), 1.55-1.33 (m, 9H), 1.34-1.04 (m, 3H), 0.92 (s, 9H) **^{13}C NMR** (100 MHz, $CDCl_3$) δ 158.0, 148.8, 145.0, 140.4, 139.1, 135.6, 135.5, 133.7, 133.7, 130.9, 129.8, 129.7, 127.8, 127.7, 127.7, 126.3, 124.4, 122.1, 115.3, 102.9, 81.2, 61.6, 55.7, 46.7, 36.3, 34.3, 31.4, 29.8, 28.3, 26.8, 23.0, 22.8, 19.1; **IR** (neat) ν 3401, 2930, 1688, 1621, 1557, 1412, 1364, 1233, 1150, 1109, 831, 704 cm^{-1}; **HRMS** (ESI): *m/z*: calculated for $C_{41}H_{52}BrN_2O_5Si^+$ $[M+H]^+$: 759.2823, found 759.2812.

1.2.11 Synthesis of alcohol 20 from epi-20

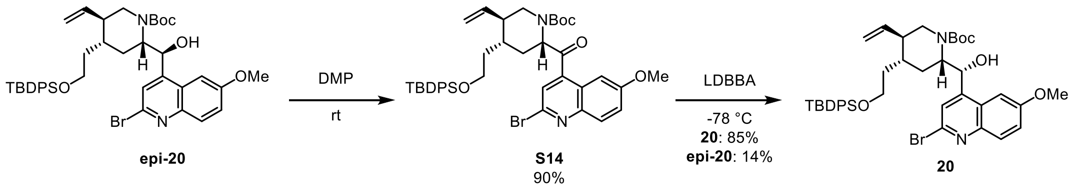

To a mixture of **epi-20** (50.0 mg, 65.8 μmol) in CH_2Cl_2 (1.3 mL), DMP (83.7 mg, 197 μmol) was added at 0 °C. After stirring for 5 h at room temperature, the reaction was quenched with saturated aqueous $NaHCO_3$. The aqueous layer was extracted with CH_2Cl_2 three times. The combined organic layer was dried over Na_2SO_4, filtered, and concentrated in vacuo to give the crude products. Purification by silica gel column chromatography (^{n}Hexane:EtOAc = 10:1) afforded **S14** (45.0 mg, 90%) as a yellow oil. R_f = 0.54 (^{n}Hexane:EtOAc = 3:1, color reagent: ninhydrin), $[\alpha]_D^{22}$ +1.8 (c 0.86, $CHCl_3$)

^{1}H NMR (400 MHz, $CDCl_3$) δ 7.94 (d, *J* = 9.2 Hz, 1H), 7.72-7.67 (m, 2H), 7.60-7.54 (m, 4H), 7.43-7.27 (m, 7H), 5.75-5.60 (m, 1H), 5.14-5.04 (m, 2H), 4.64 (brs, 1H), 3.89 (s, 3H), 3.72-3.57 (m, 3H), 3.33 (brs, 1H), 2.14-1.93 (m, 2H), 1.92-1.77 (m, 1H), 1.68-1.50 (m, 1H), 1.47-1.23 (m, 2H), 1.35 (s, 9H), 0.94 (s, 9H); **^{13}C NMR** (100 MHz, $CDCl_3$) δ 199.7, 159.2, 145.9, 139.4, 137.8, 135.6, 135.6, 133.7, 130.3, 129.8, 129.8, 127.8, 126.1, 125.0, 123.8, 116.4, 104.0, 81.1, 61.6, 61.5, 55.8, 36.5, 35.0, 31.4, 29.6, 28.4, 28.3, 26.9, 19.2; **IR** (neat) ν 2931. 1684, 1619, 1550, 1471, 1408, 1366, 1283, 1242,

1156, 1095, 915, 736, 703 cm^{-1}; **HRMS** (ESI):m/z: calculated for $C_{41}H_{50}BrN_2O_5Si^+$ [M+H]$^+$: 757.2667, found 757.2666.

To a solution of **S14** (255 mg, 0.336 mmol) in CH_2Cl_2 (34 mL), LDBBA[4,5] (2.75 mL, 0.50 M in THF, 1.35 mmol) was slowly added at -20℃. After being stirred for 15 min, the reaction was quenched by adding saturated aqueous NH_4Cl. The aqueous layer was extracted with CH_2Cl_2 three times. The combined organic layer was dried over Na_2SO_4, filtered, and concentrated in vacuo to give the crude products. Purification by silica gel column chromatography (^{n}Hexane:EtOAc = 10:1) afforded **20** (218 mg, 85%) as a pale-yellow amorphous solid and **epi-20** (35.8 mg, 14%) as a pale-yellow amorphous solid.

1.2.12 Synthesis of (−)-Quinine (1)

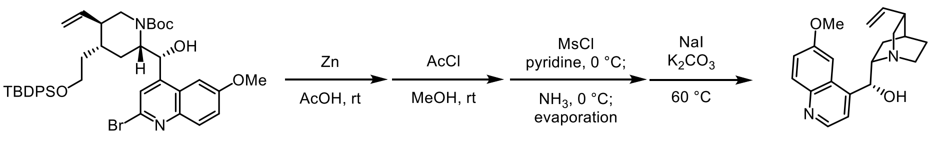

20

(−)-quinine (**1**)
55%

To a solution of **20** (200 mg, 0.263 mmol) in AcOH (5.3 mL), Zn powder (86.0 mg, 1.32 mmol) was added. After stirring for 13 h, the solution of AcCl (558 μL, 7.90 mmol) in MeOH (5.3 mL) was added to the reaction mixture. After stirring for 2 h, solvent was removed under in vacuo. To the solution of the resulting mixture in CH_2Cl_2 (5.3 mL) and THF (1.3 mL) were added MsCl (0.102 mL, 1.32 mmol) and pyridine (0.531 mL, 6.58 mmol) at 0 ℃. After stirring for 30 minutes, NH_3 (8.55 mL, 2 M in MeOH, 17.1 mmol) was added to the reaction mixture. The solvents were removed in vacuo after stirring for 30 min. To the reaction mixture in MeCN (26 mL), NaI (197 mg, 1.32 mmol) and K_2CO_3 (909 mg, 6.58 mmol) were added. After stirring for 6 h at 60 ℃, the reaction mixture was cooled to room temperature and quenched with 1 M NaOH solution. The resulting mixture was extracted with CH_2Cl_2 five times. The combined organic layer was dried over Na_2SO_4, filtered, and concentrated in vacuo to give the crude products. The residue was purified by silica gel column chromatography (SiO_2-NH, CH_2Cl_2:MeOH=100:1) to give **1** (47.0 mg, 55%) as a white amorphous solid. R_f = 0.40 (MeOH:CH_2Cl_2 = 1:6, color reagent: ninhydrin), $[\alpha]_D^{23}$ -168.6 (c 0.80, MeOH).

^{1}H NMR (400 MHz, $CDCl_3$) δ 8.36 (d, J = 4.8 Hz, 1H), 7.82 (d, J = 9.6 Hz, 1H), 7.42 (d, J = 4.8 Hz, 1H), 7.24-7.19 (m, 2H), 5.78-5.60 (m, 2H), 5.47 (brs, 1H), 4.96-4.82 (m, 2H), 3.86 (s, 3H), 3.51-3.40 (m, 1H), 3.11-2.96 (m, 2H), 2.64-3.51 (m, 2H), 2.21 (brs, 1H), 1.81-1.63 (m, 3H), 1.51-1.37 (m, 2H); **^{13}C NMR** (100 MHz, $CDCl_3$) δ 157.7, 148.6, 147.3, 143.9, 142.0, 131.2, 126.7, 121.4, 118.6, 114.4, 101.5, 71.8, 60.0, 57.0, 55.7, 43.3, 40.1, 28.0, 27.7, 21.6; **IR** (neat) ν 2933, 1621, 1591, 1509, 1472, 1432, 1362, 1242, 1175, 1098, 1030, 1002, 915, 830, 732, 642, 532, 506 cm^{-1}; **HRMS** (ESI):*m/z*: calculated for $C_{20}H_{25}N_2O_2^+$ [M+H]$^+$: 325.1911, found 325.1912. All spectra and optical rotation were in accordance with an authentic sample and the literature[6].

1.2.13 Synthesis of Br(C2')-quinine 25

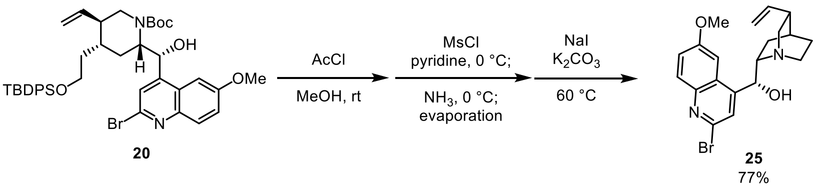

To a solution of **20** (20 mg, 26.3 μmol) in CH_2Cl_2 (530 μL), the solution of AcCl (120 μL, 1.58 mmol) in MeOH (530 μL) was added to the reaction mixture. After stirring for 12 h, solvent was removed under in vacuo. To the solution of the resulting mixture in CH_2Cl_2 (530 μL) and THF (133 μL) were added MsCl (10.2 μL, 132 μmol) and pyridine (21.2 μL, 263 μmol) at 0 ℃. After stirring for 30 minutes, NH_3 (263 μL, 2 M in MeOH, 526 μmol) was added to the reaction mixture. The solvents were removed in vacuo after stirring for 30 min. To the reaction mixture in MeCN (2.6 mL), NaI (20.0 mg, 132 μmol) and K_2CO_3 (36.4 mg, 263 μmol) were added. After stirring for 4 h at 60 ℃, the reaction mixture was cooled to room temperature and quenched with 1 M NaOH solution. The resulting mixture was extracted with CH_2Cl_2 five times. The combined organic layer was dried over Na_2SO_4, filtered, and concentrated in vacuo to give the crude products. The residue was purified by PTLC (SiO_2-NH, CH_2Cl_2:MeOH=10:1) to give **25** (8.2 mg, 77%) as a white amorphous solid. R_f = 0.40 (MeOH:CH_2Cl_2 = 1:6, color reagent: ninhydrin), $[\alpha]_D^{28}$ -53.4 (c 1.27, $CHCl_3$).
^{1}H NMR (400 MHz, $CDCl_3$) δ 7.67 (s, 1H), 7.44 (d, J= 9.2 Hz, 1H), 6.89 (d, J= 9.2 Hz, 1H), 6.73 (s, 1H), 6.28 (s, 1H), 6.07 (brs, 1H), 5.46 (ddd, J= 16.8, 10.0, 6.8 Hz, 1H), 5.01-4.88 (m, 2H), 4.50-4.36 (m, 1H), 3.64 (s, 3H), 3.39 (t, J= 12.0 Hz, 1H), 3.20 (t, J= 8.8 Hz, 1H), 3.08 (dt, J= 11.6, 4.8 Hz, 1H), 3.00-2.92 (m, 1H), 2.66 (brs, 1H), 2.24-2.11 (m, 1H), 2.07-1.95 (m, 2H), 1.89-1.74 (m, 1H), 1.24 (brt, J= 11.6 Hz, 1H); **^{13}C NMR** (100 MHz, $CDCl_3$) δ 158.5, 146.7, 144.0, 138.7, 137.1, 130.7, 124.0, 123.5, 122.9, 117.6, 100.0, 65.8, 60.1, 57.6, 55.0, 44.5, 37.2, 26.9, 24.3, 18.4; **IR** (neat) ν 2937, 1620, 1578, 1557, 1506, 1454, 1411, 1288, 1235, 1175, 1098, 1033, 910, 831, 733, 648 cm^{-1}; **HRMS** (ESI):m/z: calculated for $C_{20}H_{24}BrN_2O_2^+$ $[M+H]^+$: 403.1016, found 403.1018. All spectra were in accordance with the literature[7].

1.2.14 Synthesis of 26

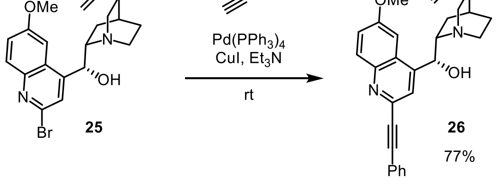

Toluene (1.0 mL), THF (0.72 mL) and Et_3N (0.30 mL) were degassed by bubbling argon for a few minutes. **25** (20.0 mg, 49.6 μmol), ethynylbenzene (30.0 μL, 273 μmol), CuI (2.3 mg, 12 μmol) and $Pd(PPh_3)_4$ (2.9 mg, 2.5 μmol) were added to the prepared solution at room temperature. After stirring

for 1 h at the same temperature, solvents were removed in vacuo. The crude mixture was purified by PTLC (SiO_2-NH, CH_2Cl_2:MeOH = 15:1) afforded **26** (16.2 mg, 77%) as a pale-yellow amorphous solid. R_f = 0.55 (CH_2Cl_2: MeOH = 6:1, color reagent: Cerium molybdate stain), $[\alpha]_D^{25}$ +23.6 (c 0.77, $CHCl_3$)

^{1}H NMR (400 MHz, $CDCl_3$) δ 7.87 (d, J = 9.2 Hz, 1H), 7.81 (s, 1H), 7.68-7.59 (m, 2H), 7.42-7.35 (m, 3H), 7.19 (dd, J = 9.2, 2.4 Hz, 1H), 7.03 (d, J = 2.4 Hz, 1H), 5.94 (brs, 1H), 5.64 (ddd, J = 17.6, 10.4, 7.2 Hz, 1H), 5.01-4.91 (m, 2H), 4.01-3.87 (m, 1H), 3.79 (s, 3H), 3.28-3.14 (m, 2H), 2.93-2.76 (m, 2H), 2.45 (brs, 1H), 2.03-1.89 (m, 3H), 1.71-1.69 (m, 1H), 1.50-1.39 (m, 1H), 1.35-1.22 (m, 1H); **^{13}C NMR** (100 MHz, $CDCl_3$) δ 158.6, 146.2, 144.2, 140.6, 139.8, 132.3, 131.6, 129.1, 128.5, 122.5, 122.4, 122.1, 115.9, 100.7, 89.8, 89.2, 77.4, 60.1, 56.4, 56.2, 43.8, 38.9, 27.5, 27.5, 26.3, 20.2; **IR** (neat) ν 3368, 2935, 1620, 1592, 1551, 1496, 1474, 1408, 1358, 1234, 1118, 1027, 917, 832, 756, 690, 586 cm^{-1};**HRMS** (ESI):*m/z*: calculated for $C_{28}H_{29}N_2O_2^+$ $[M+H]^+$: 425.2224, found 425.2215.

1.2.15 Synthesis of 27

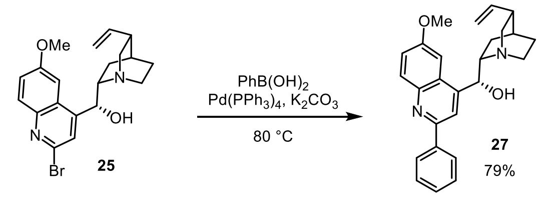

Toluene (1.0 mL) and MeOH (1.0 mL) were degassed by bubbling argon for a few minutes. **25** (20.0 mg, 49.6 μmol), K_2CO_3 (13.7 mg, 99.2 μmol), $Pd(PPh_3)_4$ (5.7 mg, 5.0 μmol) and $PhB(OH)_2$ (9.1 mg, 74 μmol) were added to the prepared solution at room temperature. After stirring for 1 h at 80 ℃, the reaction mixture was cooled to room temperature and quenched with water. The resulting mixture was extracted with CH_2Cl_2 five times. The combined organic layer was combined, dried over Na_2SO_4, filtered, and concentrated in vacuo to give the crude products. The crude mixture was purified by PTLC (SiO_2-NH, CH_2Cl_2:MeOH = 15:1) afforded **27** (15.6 mg, 79%) as an off-white amorphous solid. R_f = 0.45 (CH_2Cl_2: MeOH = 6:1, color reagent: Cerium molybdate stain), $[\alpha]_D^{25}$ -26.0 (c 0.77, $CHCl_3$)

^{1}H NMR (400 MHz, $CDCl_3$) δ 8.11-8.04 (m, 3H), 7.96 (s, 1H), 7.51-7.38 (m, 3H), 7.33 (dd, J = 9.2, 2.8 Hz, 1H), 7.16 (d, J = 2.8 Hz 1H), 5.72 (ddd, J = 18.0, 10.4, 7.6 Hz, 1H), 5.61 (brs, 1H), 5.00-4.87 (m, 2H), 3.89 (s, 3H), 3.61-3.49 (m, 1H), 3.20-3.08 (m, 2H), 2.78-2.65 (m, 2H), 2.30 (brs, 1H), 1.86-1.71 (m, 3H), 1.58-1.46 (m, 2H), 1.31-1.20 (m, 1H); **^{13}C NMR** (100 MHz, $CDCl_3$) δ 157.8, 154.7, 147.9, 144.5, 141.8, 139.8, 132.0, 129.8, 129.1, 128.9, 127.4, 125.5 121.9, 116.3, 115.7, 114.7, 101.1, 72.2, 60.0, 57.1, 55.8, 43.4, 40.0, 28.0, 27.6, 21.6; **IR** (neat) ν 3326, 2931, 1621, 1597, 1557, 1498, 1456, 1352, 1230, 1031, 916, 832, 755, 696 cm^{-1}; **HRMS** (ESI):*m/z*: calculated for $C_{26}H_{29}N_2O_2$ [M+H]: 401.2224, found 401.2230.

1.2.16 Synthesis of 28

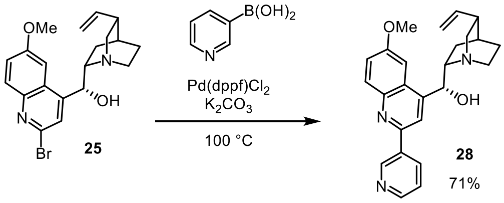

1,4-dioxane (600 μL) and H_2O (120 μL) were degassed by bubbling argon for a few minutes. **25** (20.0 mg, 49.6 μmol), K_2CO_3 (13.7 mg, 99.2 μmol), Pd(dppf)Cl_2 (3.6 mg, 5.0 μmol) and pyridin-3-ylboronic acid (9.1 mg, 74 μmol) were added to the prepared solution at room temperature. After stirring for 30 minutes at 100 ℃, the reaction mixture was cooled to room temperature and quenched with water. The resulting mixture was extracted with CH_2Cl_2 five times. The combined organic layer was dried over Na_2SO_4, filtered, and concentrated in vacuo to give the crude products. The crude mixture was purified by PTLC (SiO_2-NH, CH_2Cl_2:MeOH = 10:1) afforded **28** (14.1 mg, 71%) as a brown amorphous solid. R_f = 0.36 (CH_2Cl_2: MeOH = 6:1, color reagent: Cerium molybdate stain), $[\alpha]_D^{25}$ -18.6 (c 0.60, $CHCl_3$) **^{1}H NMR** (400 MHz, $CDCl_3$) δ 9.19 (d, *J* = 2.0 Hz, 1H), 8.60 (dd, *J* = 4.8, 2.0 Hz, 1H), 8.41 (dt, *J* = 8.0, 2.0 Hz, 1H), 8.05 (d, *J* = 9.2 Hz, 1H), 7.96 (s, 1H), 7.41-7.34 (m, 2H), 7.27-7.23 (m, 1H), 5.76 (ddd, *J* = 17.6, 10.0, 7.6 Hz, 1H), 5.56 (d, *J* = 4.0 Hz, 1H), 5.01-4.88 (m, 2H), 3.92 (s, 3H), 3,51-3.41 (m, 1H), 3.23-3.14 (m, 1H), 3.10 (dd, *J* = 13.6, 10.0 Hz, 1H), 2.74-2.63 (m, 2H), 2.23 (brs, 1H), 1.88-1.67 (m, 4H), 1.65-1.56 (m, 1H), 1.56-1.44 (m, 1H); **^{13}C NMR** (100 MHz, $CDCl_3$) δ 158.0, 151.7, 149.6, 149.2, 148.4, 144.6, 142.1, 135.4, 134.9, 132.0, 126.0, 123.8, 122.2, 116.0, 114.5, 101.4, 72.5, 60.2, 57.2, 55.8, 43.4, 40.1, 28.0, 27.8, 22.0; **IR** (neat) ν 3217, 2936, 2868, 1622, 1596, 1505, 1486, 1454, 1426, 1352, 1265, 1231, 1028, 912, 833, 733, 709 cm^{-1}; **HRMS** (ESI):*m/z*: calculated for $C_{25}H_{28}N_3O_2$ [M+H]: 402.2176, found 402.2183.

1.3 Copies of spectra

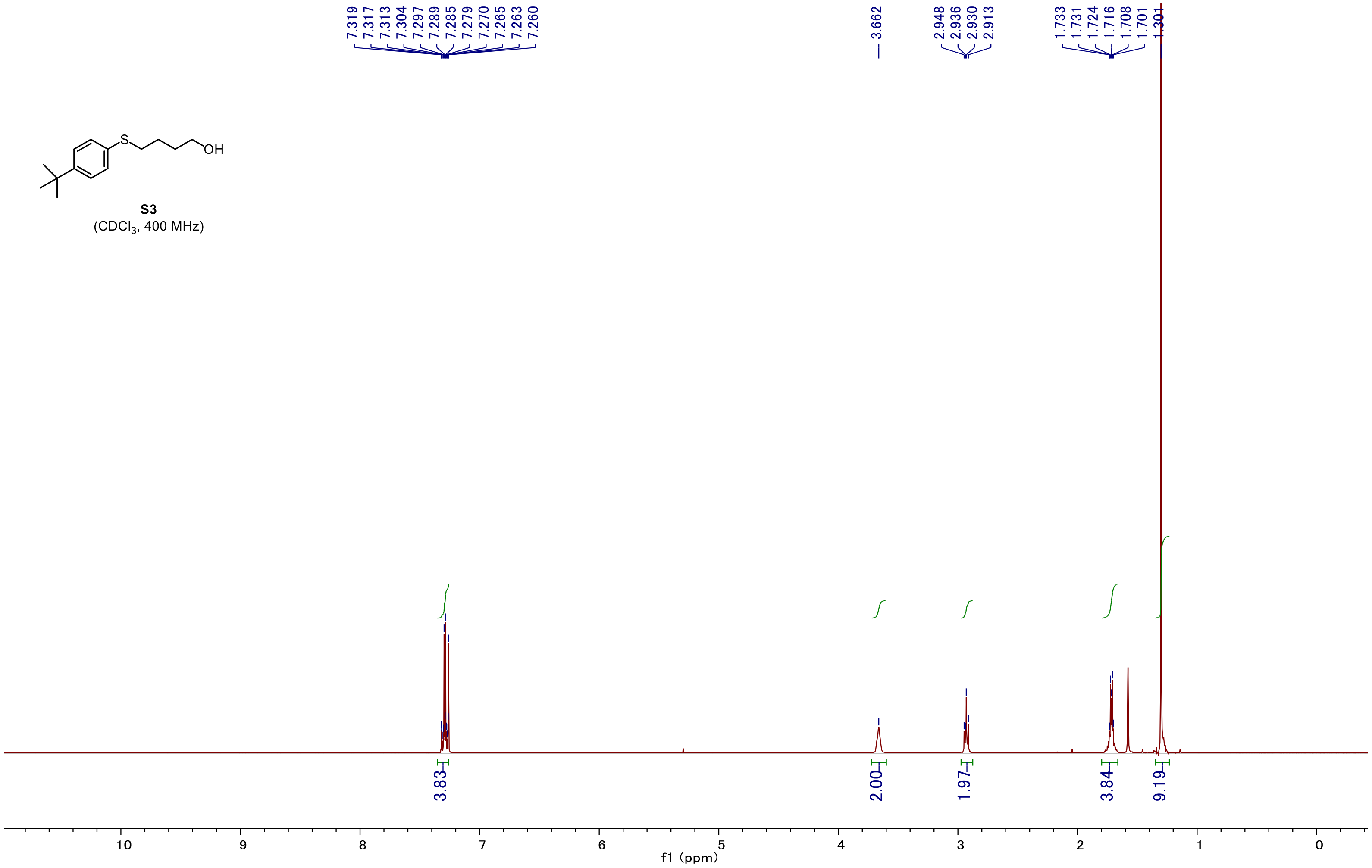

Supplementary Figure 1 ^{1}H NMR (400 MHz, $CDCl_3$) spectra of **S3**

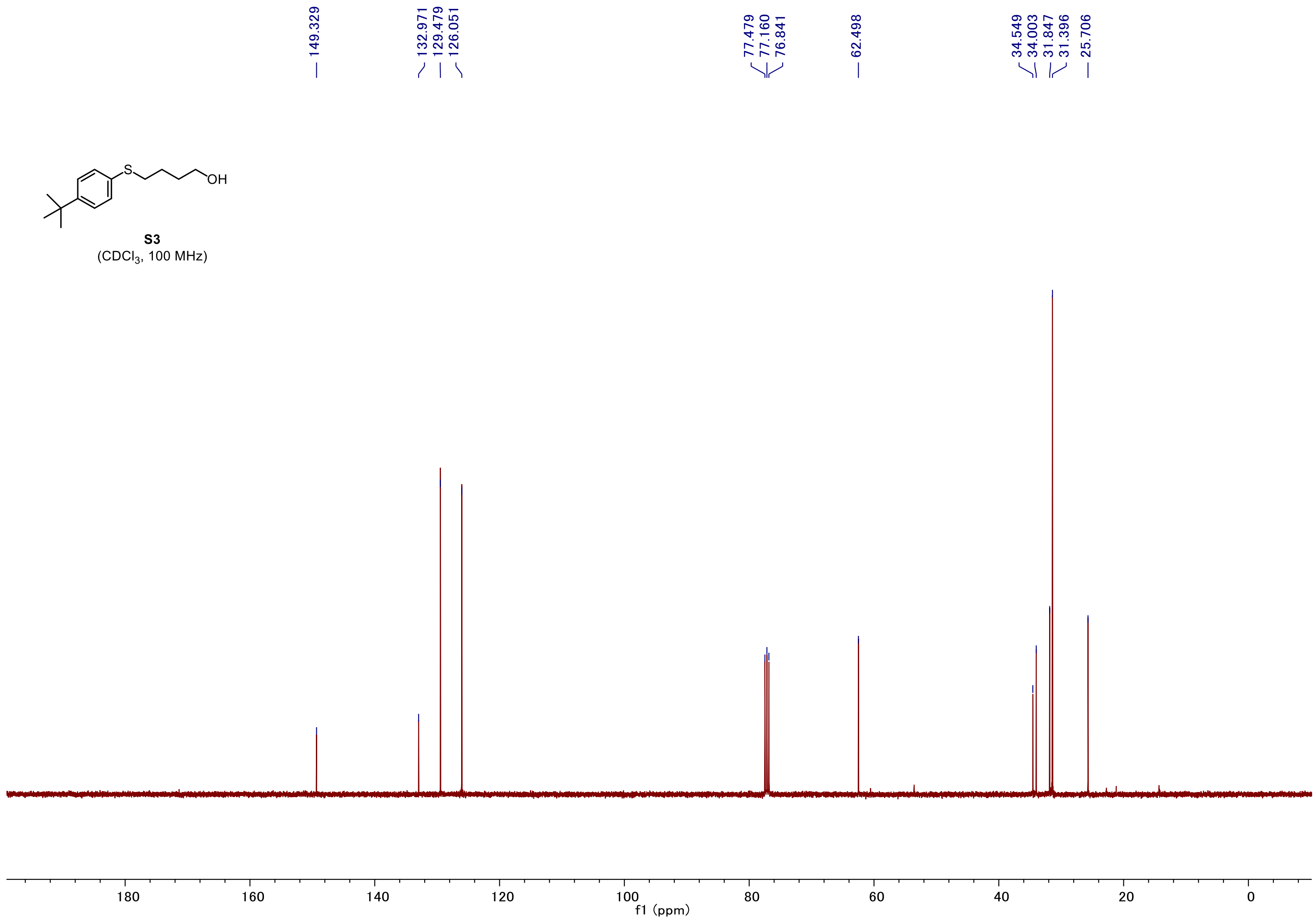

Supplementary Figure 2 ^{13}C NMR (100 MHz, $CDCl_3$) spectra of **S3**

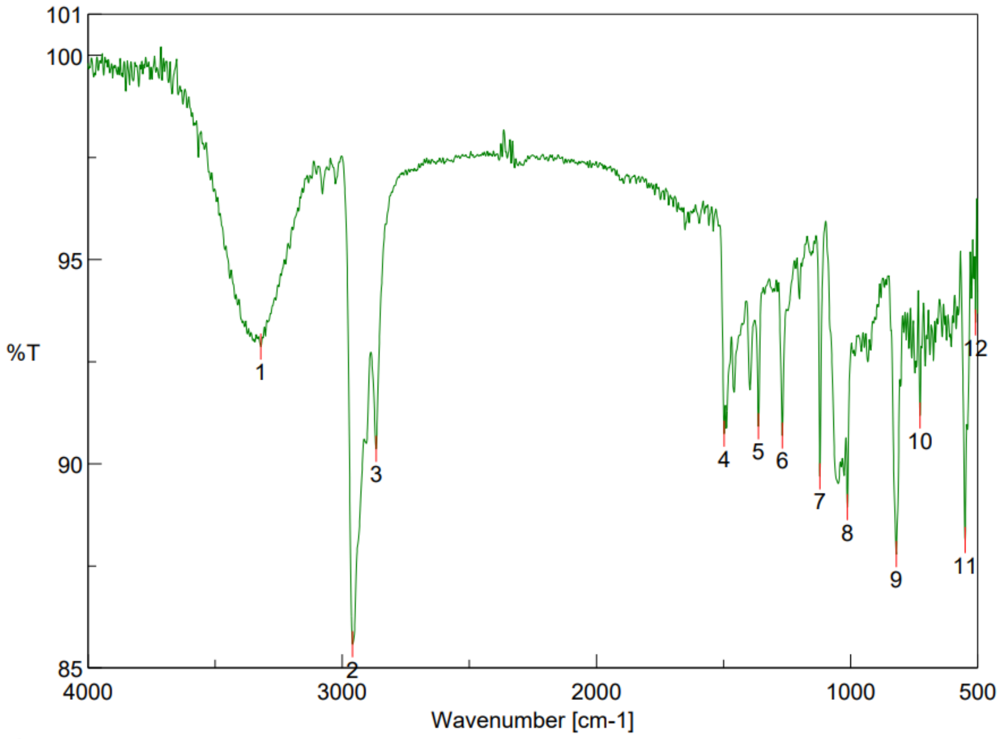

[ピーク検出結果]

No.	位置	強度	No.	位置	強度
1	3319.86	92.8656	2	2959.23	85.5764
3	2866.67	90.357	4	1497.45	90.7336
5	1362.46	90.9121	6	1267.97	90.6861
7	1120.44	89.6897	8	1012.45	88.932
9	820.563	87.7859	10	727.032	91.1804
11	549.613	88.1268	12	508.151	93.4558

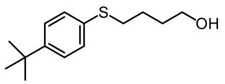

S3

Supplementary Figure 3 IR (neat) spectra of **S3**

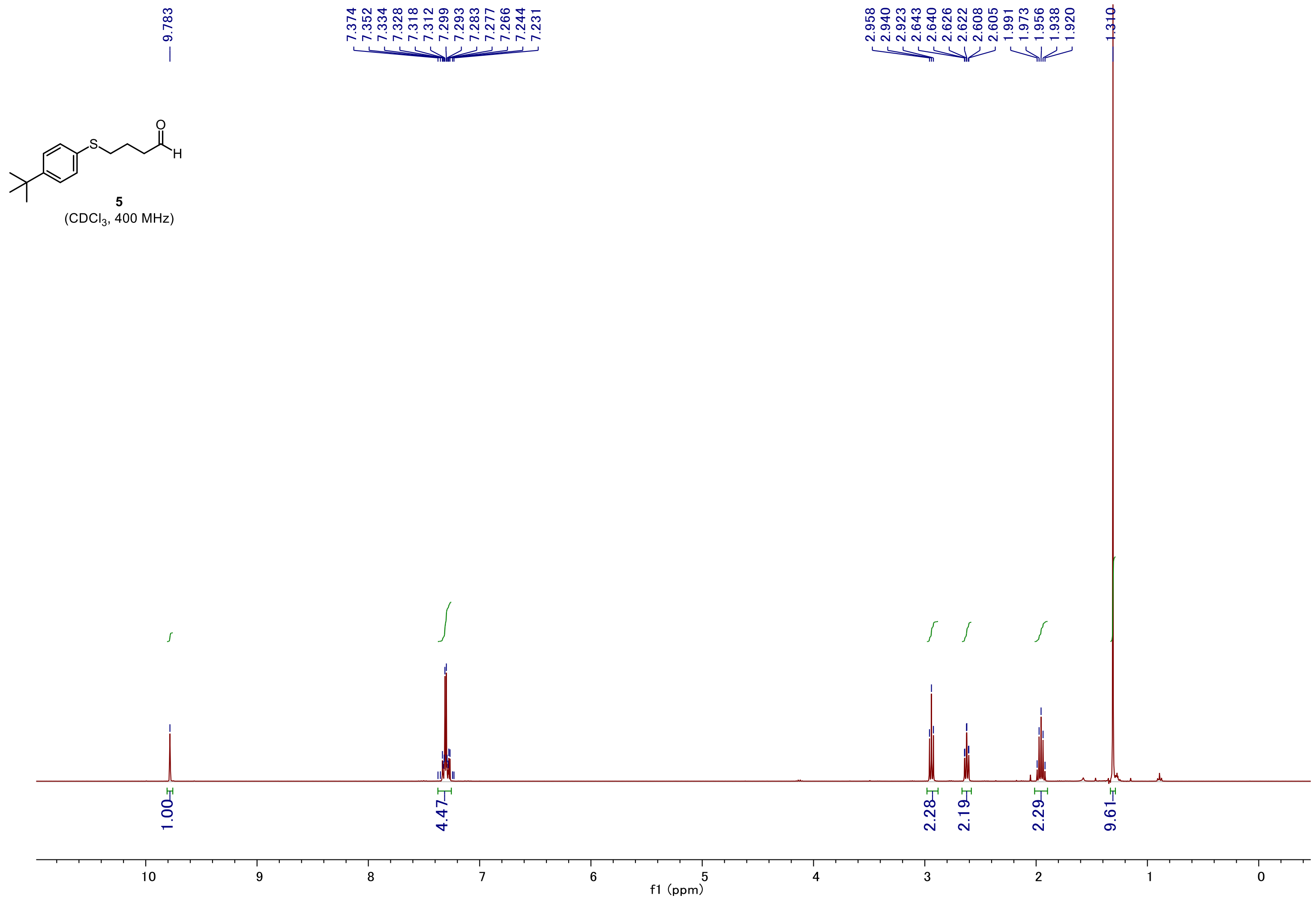

Supplementary Figure 4 ^{1}H NMR (400 MHz, $CDCl_3$) spectra of **5**

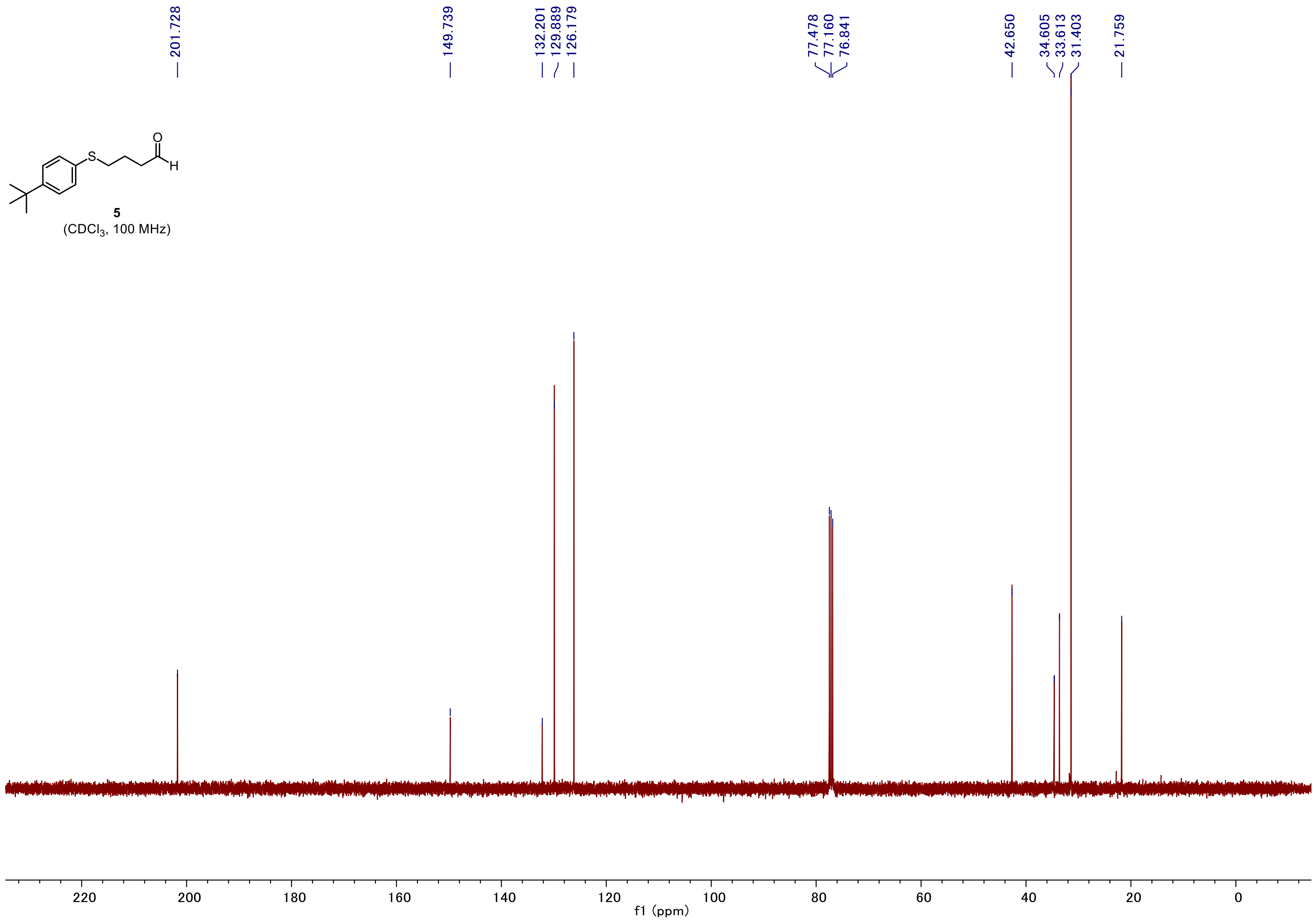

Supplementary Figure 5 ^{13}C NMR (100 MHz, $CDCl_3$) spectra of **5**

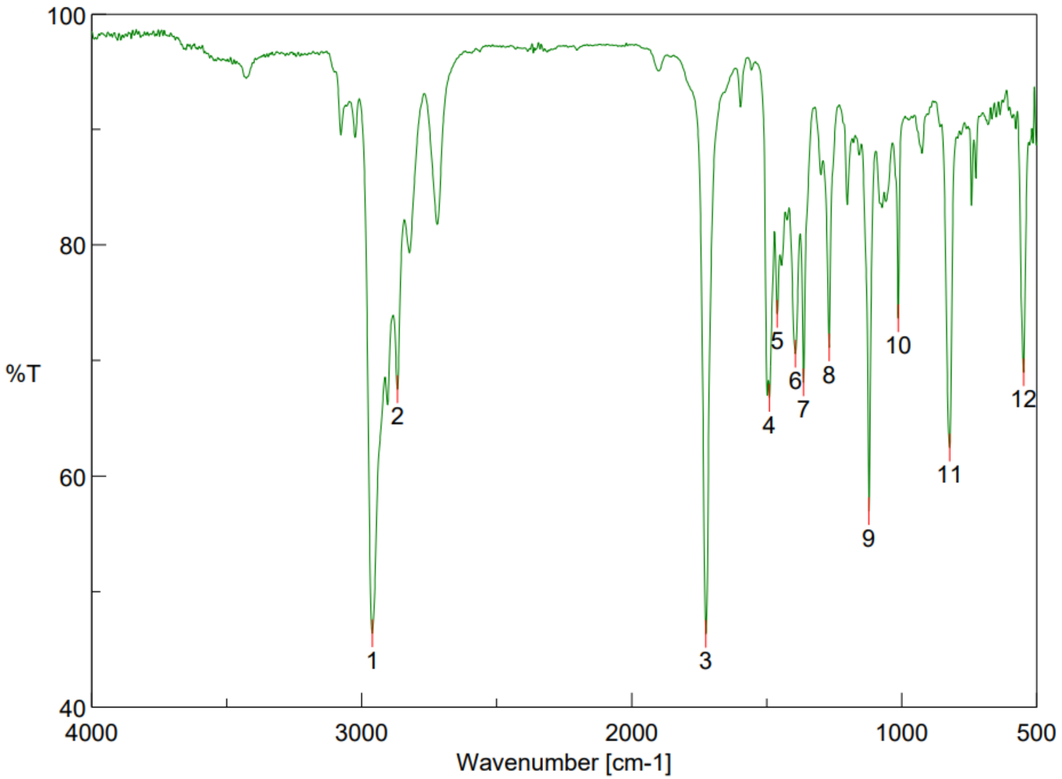

[ピーク検出結果]

No.	位置	強度	No.	位置	強度
1	2961.16	46.4005	2	2867.63	67.4819
3	1725.01	46.3464	4	1489.74	66.7376
5	1460.81	73.9958	6	1393.32	70.5662
7	1363.43	68.0956	8	1268.93	71.1228
9	1121.4	56.9556	10	1012.45	73.6431
11	822.491	62.4481	12	548.649	68.9745

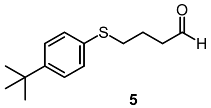

5

Supplementary Figure 6 IR (neat) spectra of **5**

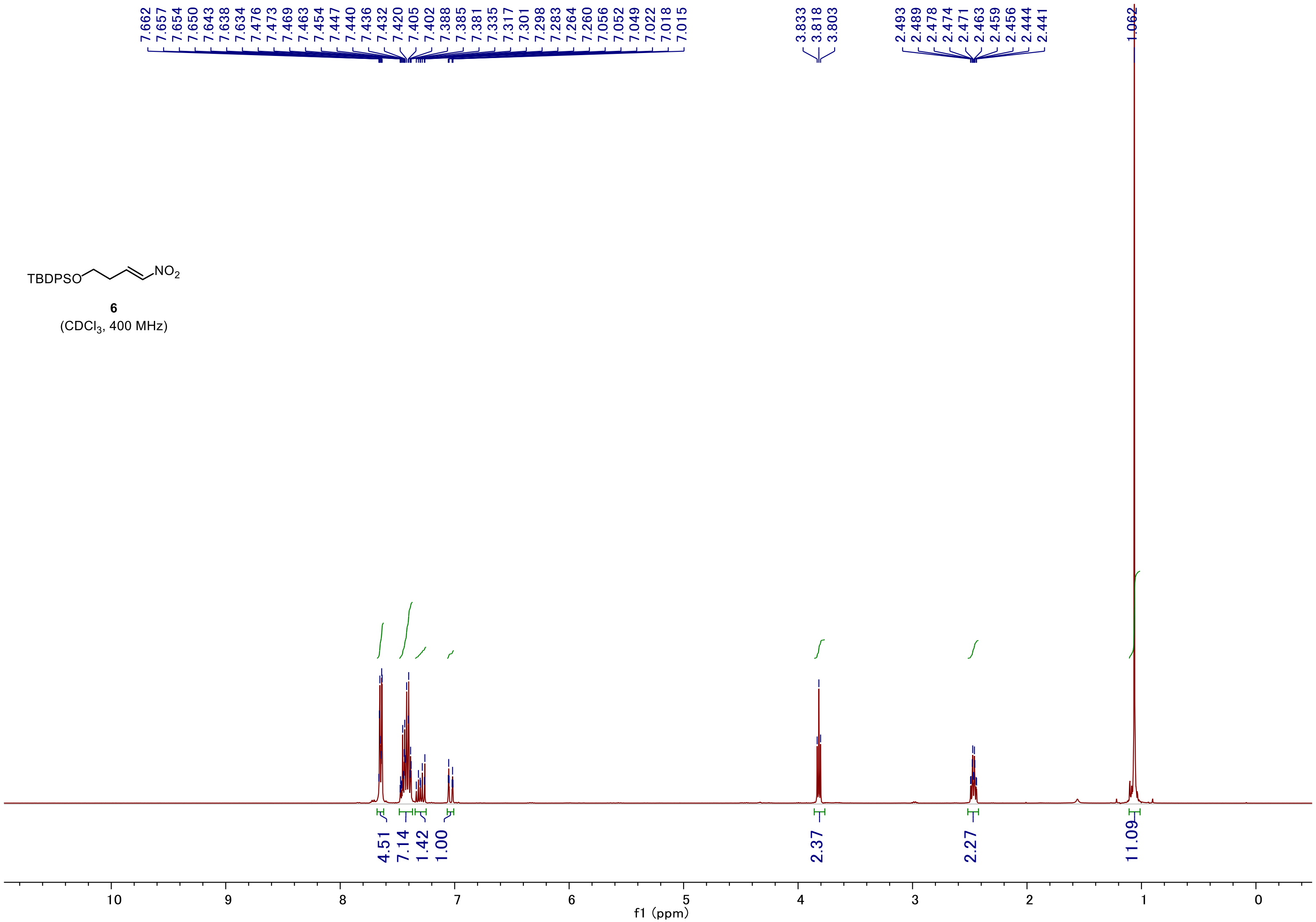

Supplementary Figure 7 ^{1}H NMR (400 MHz, $CDCl_3$) spectra of **6**

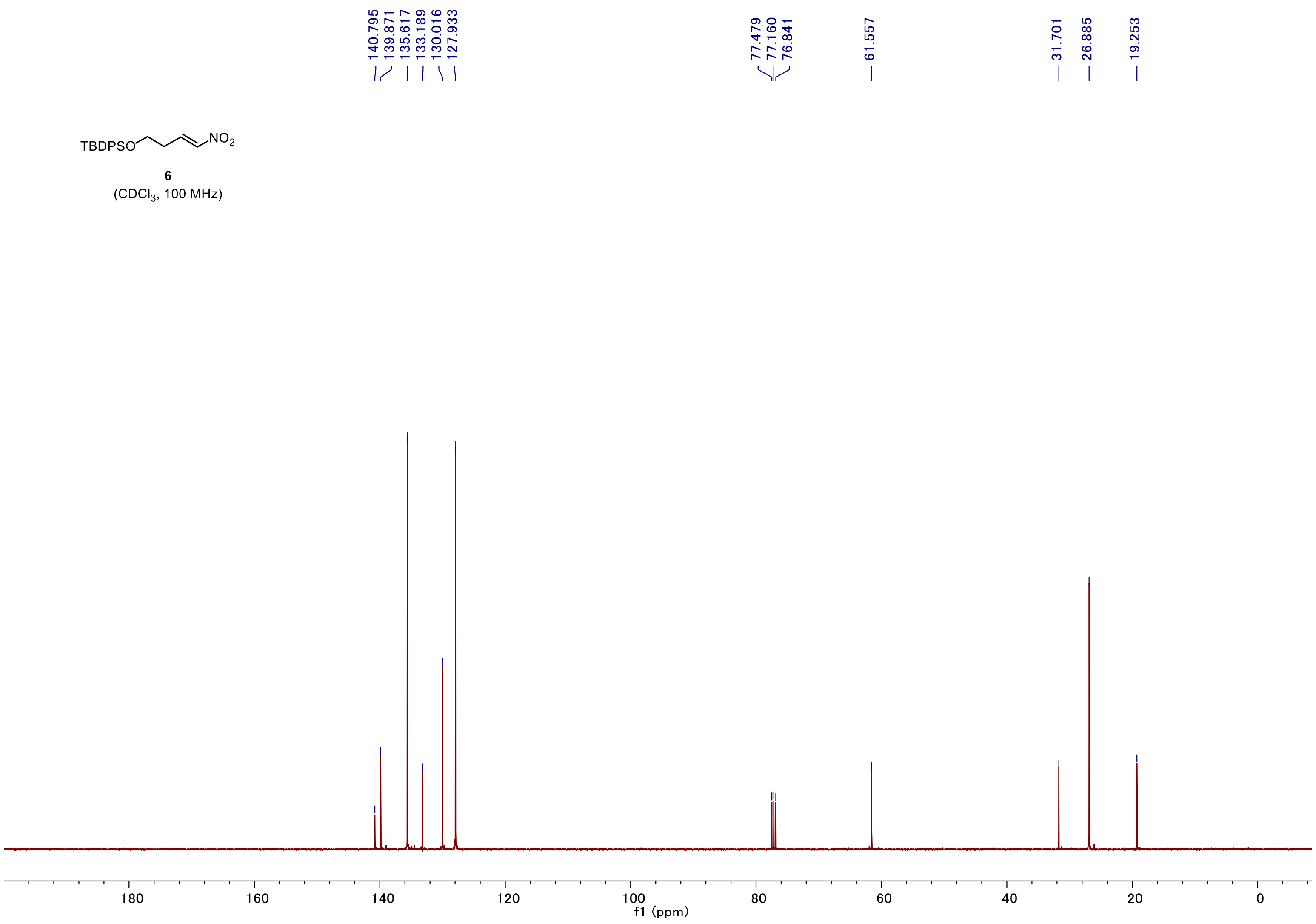

Supplementary Figure 8 ^{13}C NMR (100 MHz, $CDCl_3$) spectra of **6**

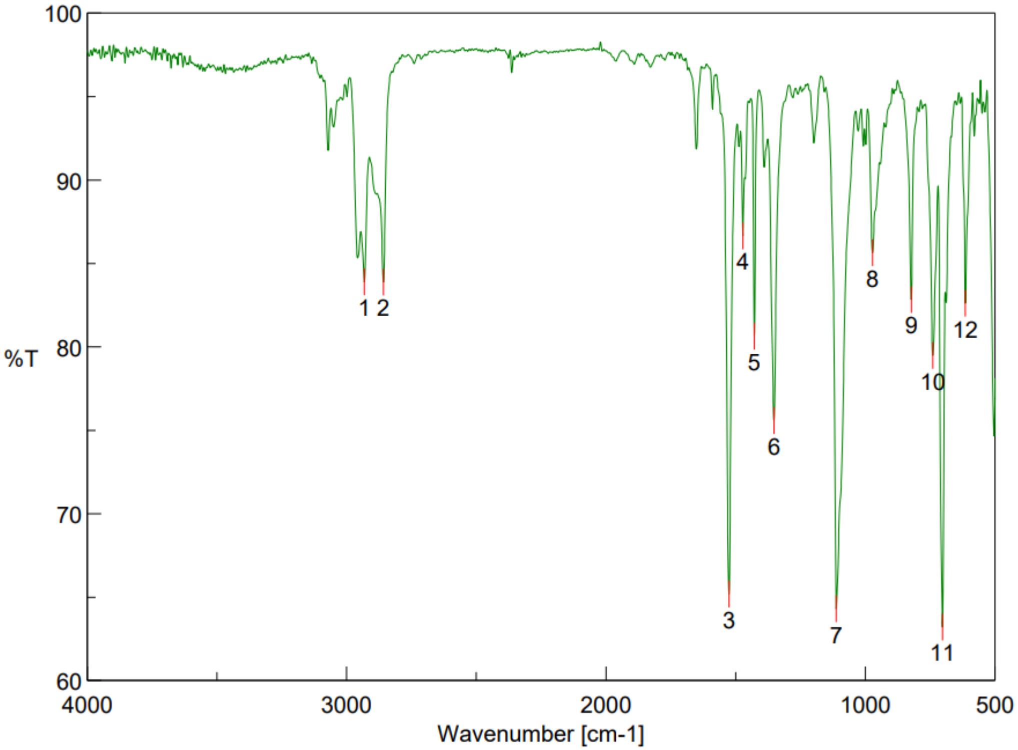

［ピーク検出結果］

No.	位置	強度	No.	位置	強度
1	2931.27	83.8821	2	2857.99	83.8721
3	1525.42	65.1786	4	1472.38	86.6316
5	1428.03	80.625	6	1351.86	75.5564
7	1111.76	64.2958	8	971.947	85.6274
9	822.491	82.8298	10	739.567	79.4699
11	701.962	63.1997	12	614.217	82.5975

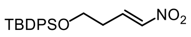

6

Supplementary Figure 9 IR (neat) spectra of **6**

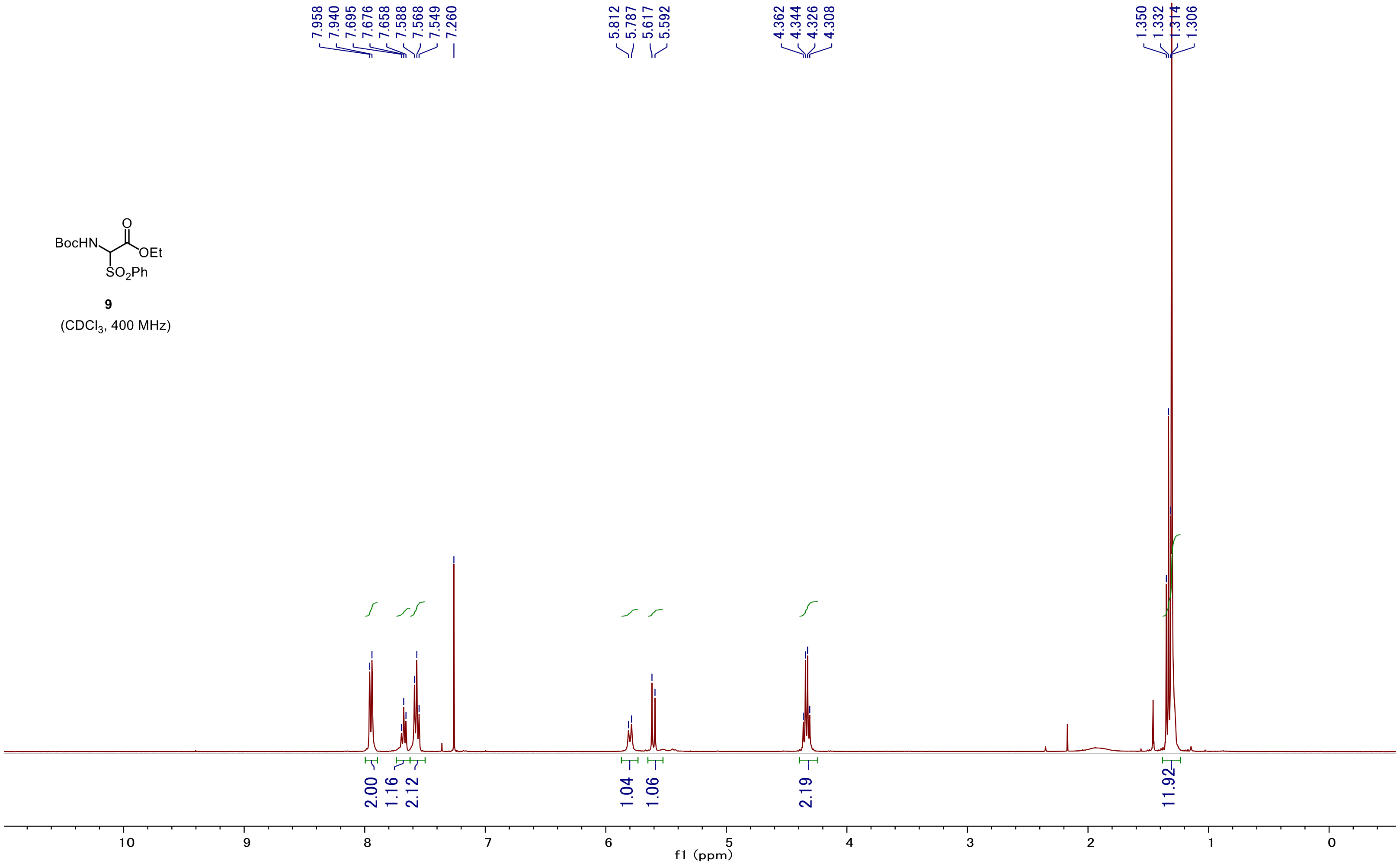

Supplementary Figure 10 ^{1}H NMR (400 MHz, $CDCl_3$) spectra of **9**

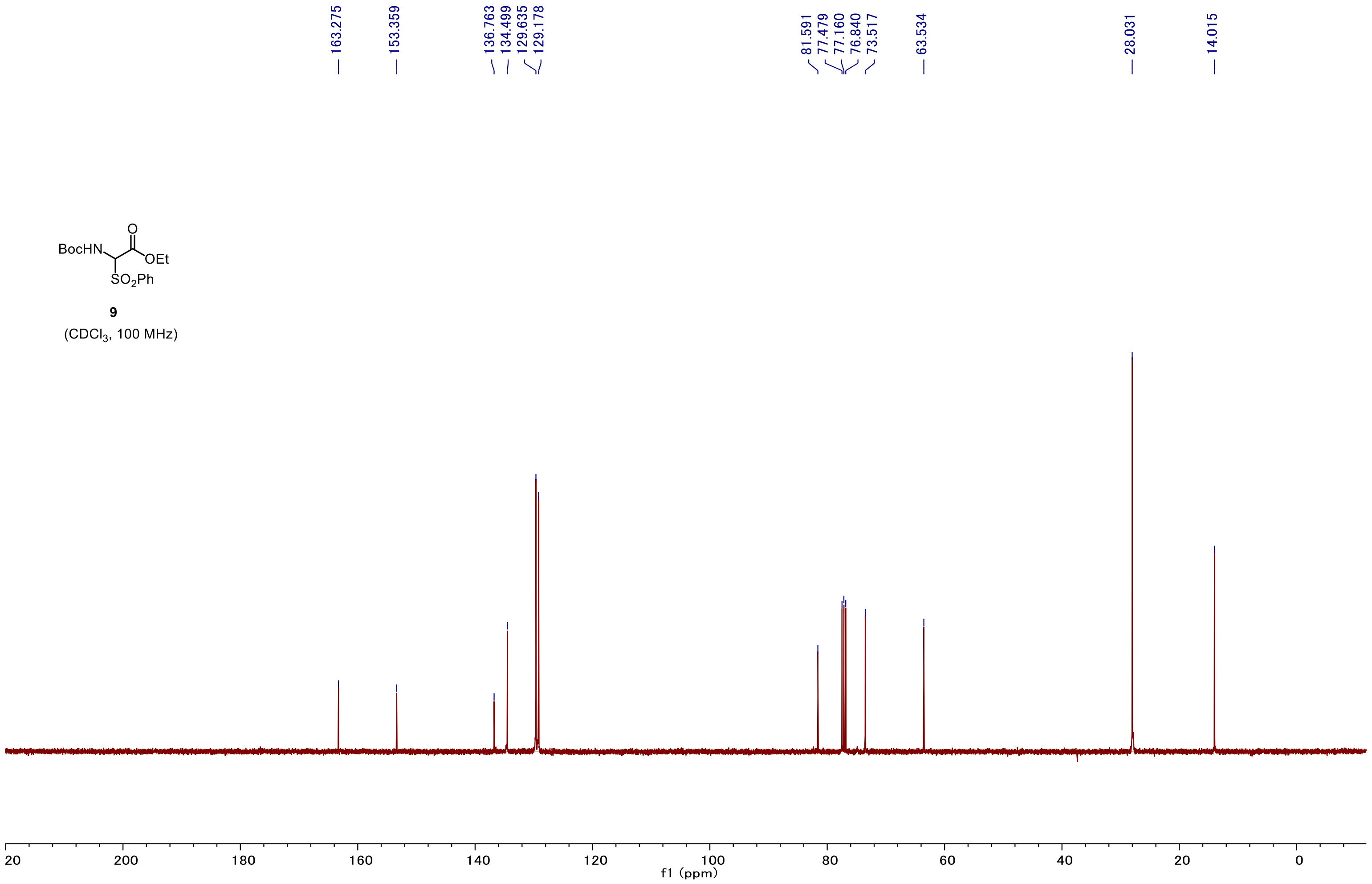

Supplementary Figure 11 ^{13}C NMR (100 MHz, $CDCl_3$) spectra of **9**

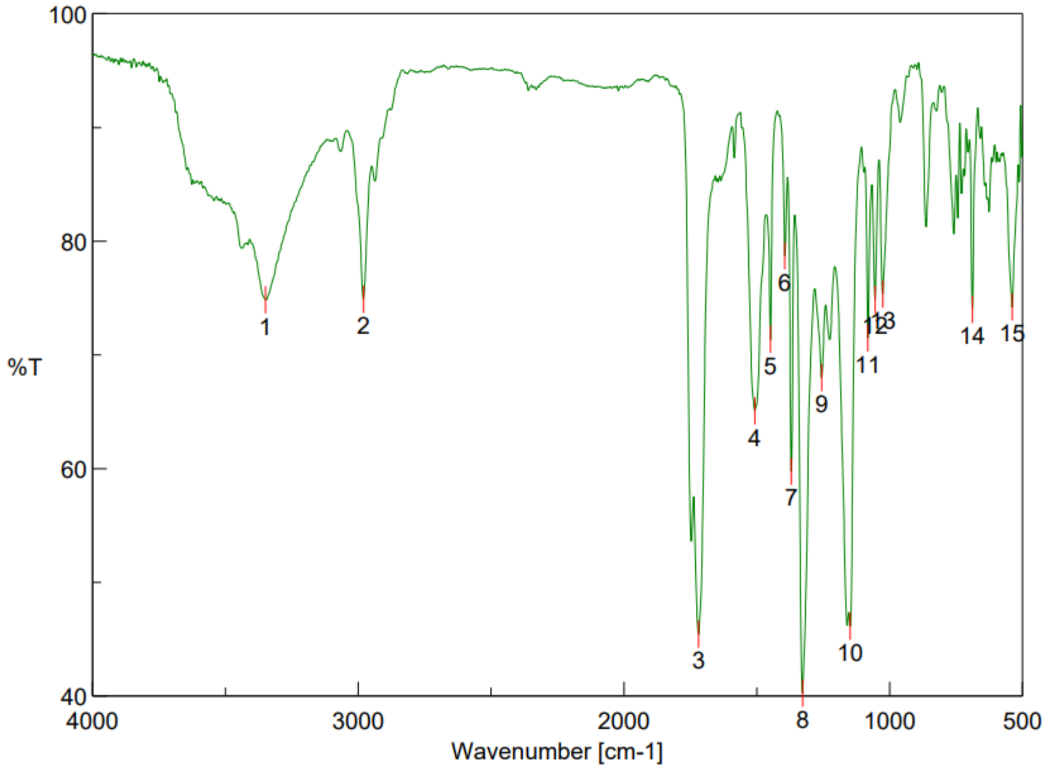

[ピーク検出結果]

No.	位置	強度	No.	位置	強度
1	3349.75	74.8228	2	2980.45	74.8853
3	1719.23	45.4415	4	1507.1	65.0726
5	1448.28	71.3652	6	1394.28	78.6975
7	1370.18	59.7551	8	1327.75	40.2502
9	1255.43	67.9874	10	1148.4	46.1436
11	1081.87	71.484	12	1054.87	74.8483
13	1025.94	75.3197	14	688.463	73.9731
15	538.042	74.2187			

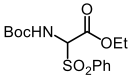

9

Supplementary Figure 12 IR (neat) spectra of **9**

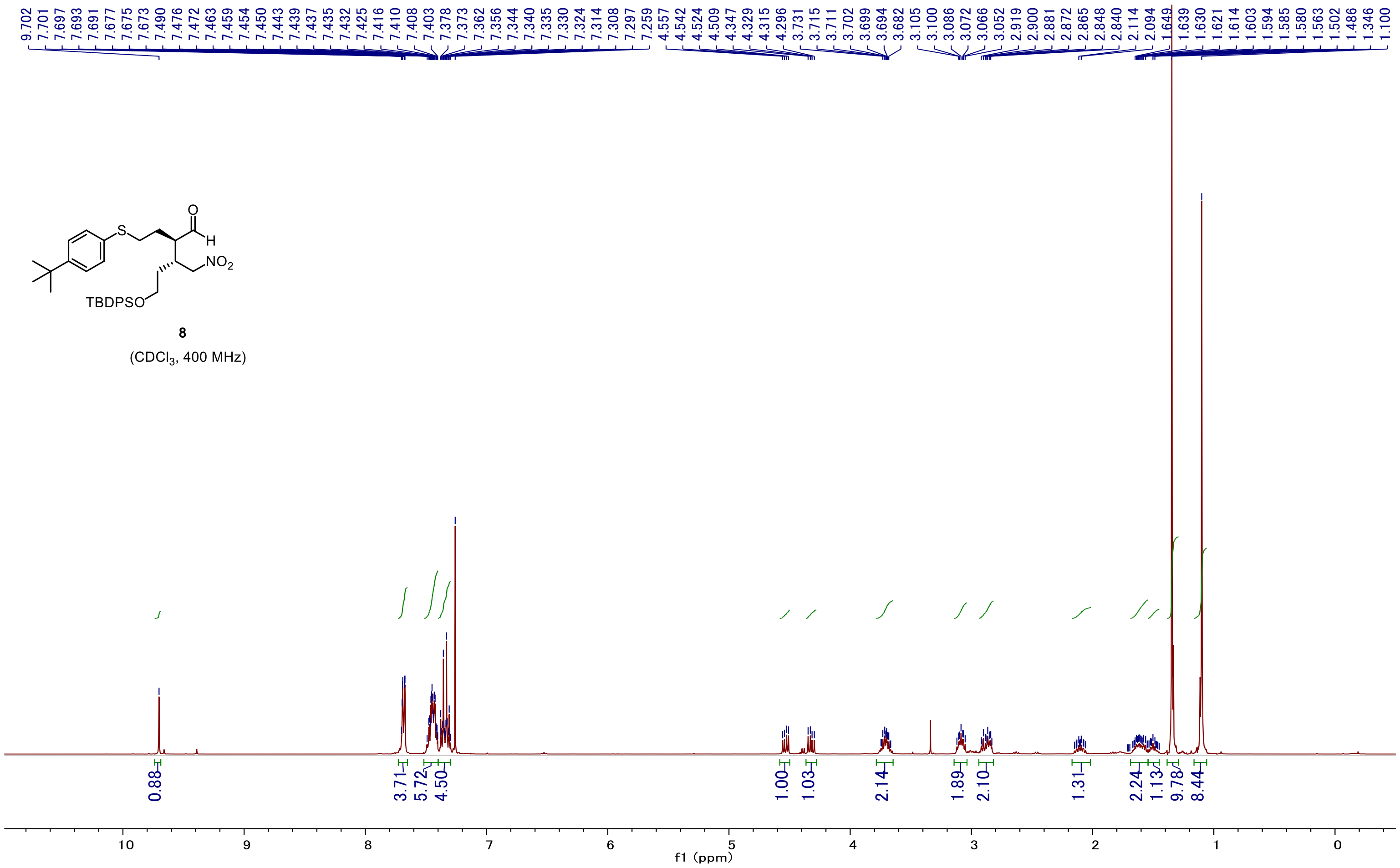

Supplementary Figure 13 ^{1}H NMR (400 MHz, $CDCl_3$) spectra of **8**

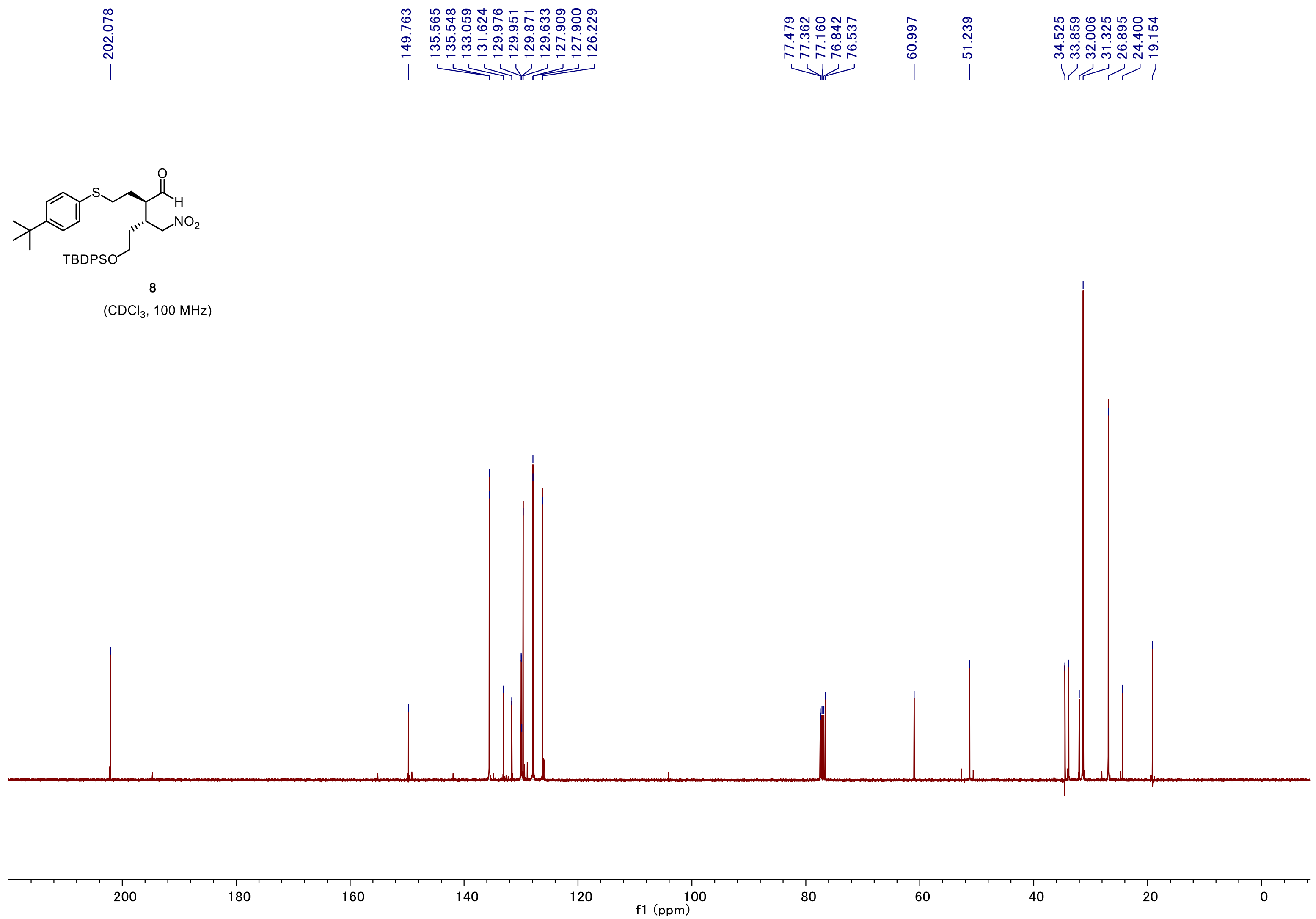

Supplementary Figure 14 ^{13}C NMR (100 MHz, $CDCl_3$) spectra of **8**

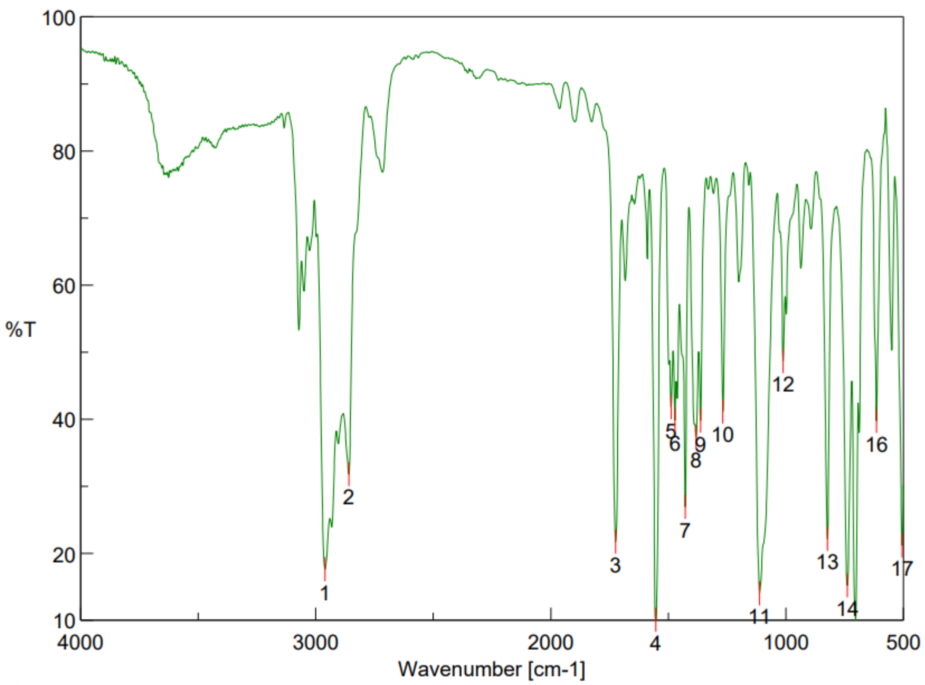

[ピーク検出結果]

No.	位置	強度	No.	位置	強度
1	2960.2	17.616	2	2858.95	31.8044
3	1724.05	21.7107	4	1553.38	10.0931
5	1487.81	41.7598	6	1472.38	39.8433
7	1428.03	26.9331	8	1381.75	37.3369
9	1362.46	39.7698	10	1267.97	41.1082
11	1111.76	14.004	12	1011.48	48.662
13	823.455	22.1495	14	738.603	15.2219
15	702.926	10.0183	16	614.217	39.7207
17	505.258	21.1557			

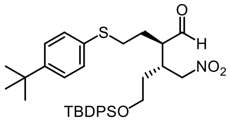

8

Supplementary Figure 15 IR (neat) spectra of **8**

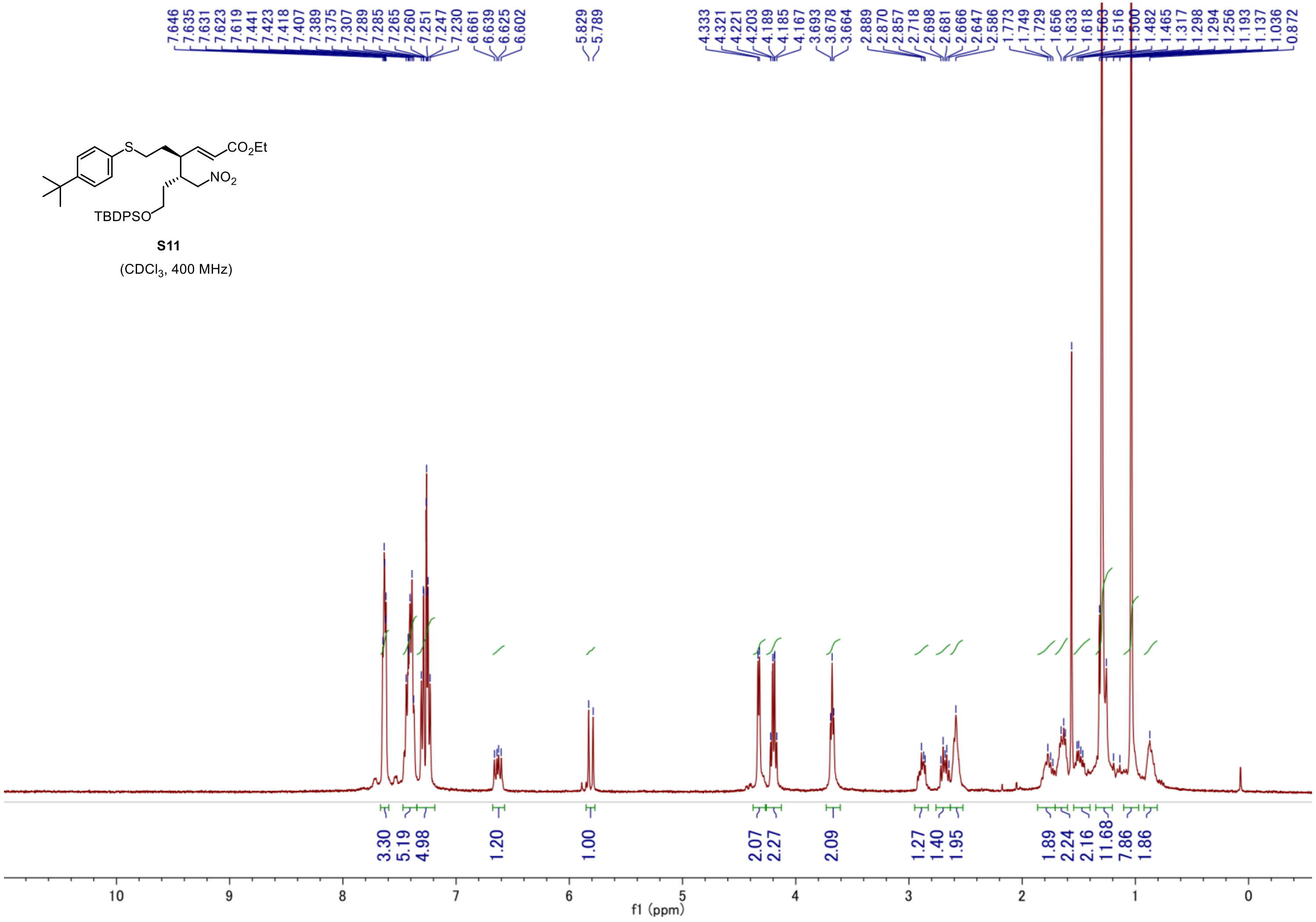

Supplementary Figure 16 ^{1}H NMR (400 MHz, $CDCl_3$) spectra of **S11**

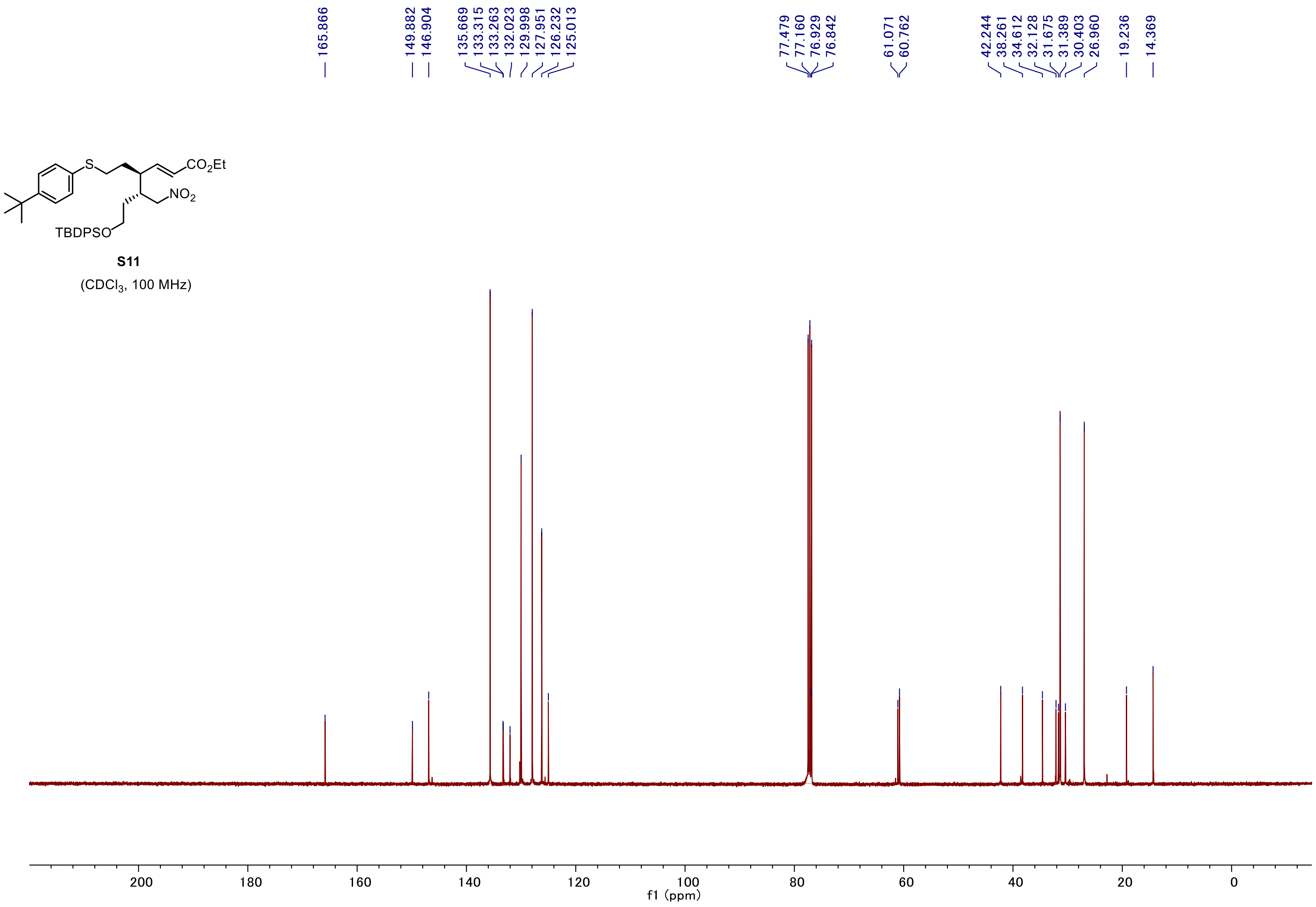

Supplementary Figure 17 ^{13}C NMR (100 MHz, $CDCl_3$) spectra of **S11**

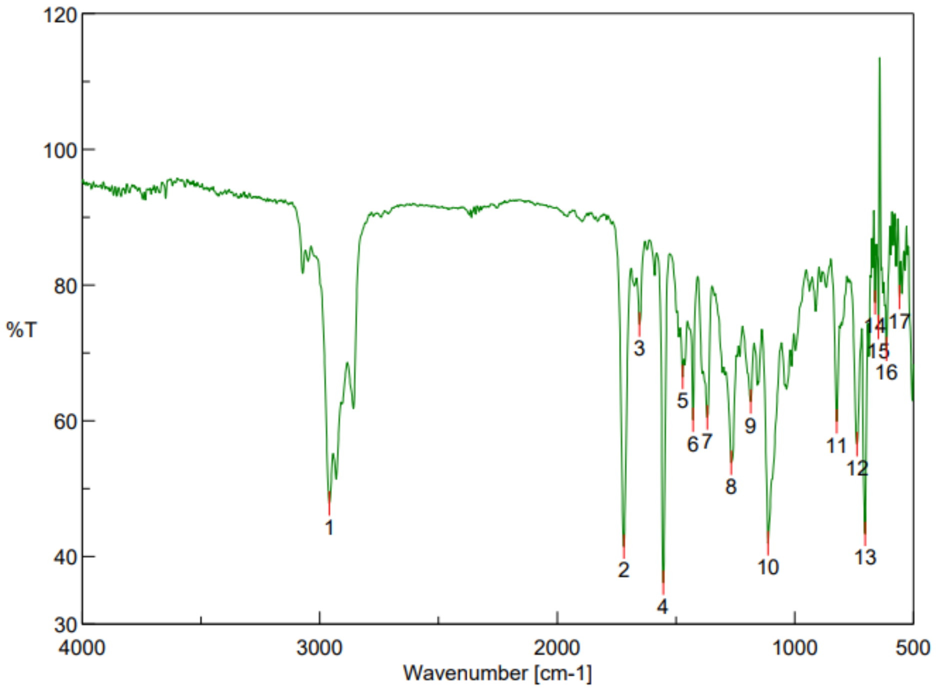

[ピーク検出結果]

No.	位置	強度	No.	位置	強度
1	2959.23	47.7735	2	1719.23	41.4124
3	1653.66	74.1641	4	1553.38	36.096
5	1472.38	66.4344	6	1428.03	60.0991
7	1368.25	60.4572	8	1267	53.7684
9	1185.04	62.8111	10	1111.76	41.9139
11	823.455	59.8965	12	737.639	56.5053
13	703.89	43.2898	14	661.464	77.4093
15	647.965	73.7837	16	613.252	70.5613
17	559.255	78.1989			

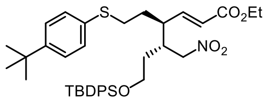

S11

Supplementary Figure 18 IR (neat) spectra of **S11**

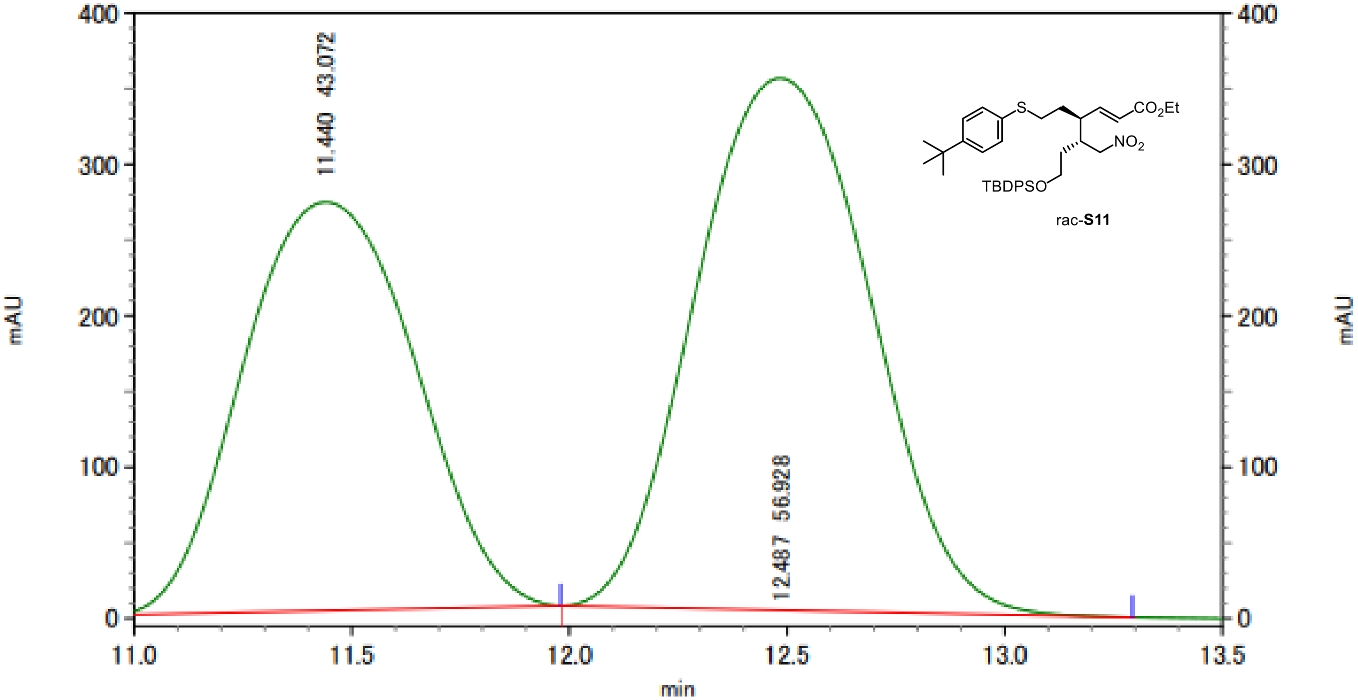

Result
1: 215.0 nm, 4.0 nm 結果

Pk #	Retention time / min	Integration/ %
1	11.440	43.072
2	12.487	56.928
合計 Tota		100.000

Supplementary Figure 19 HPLC trace for the rac-**S11**

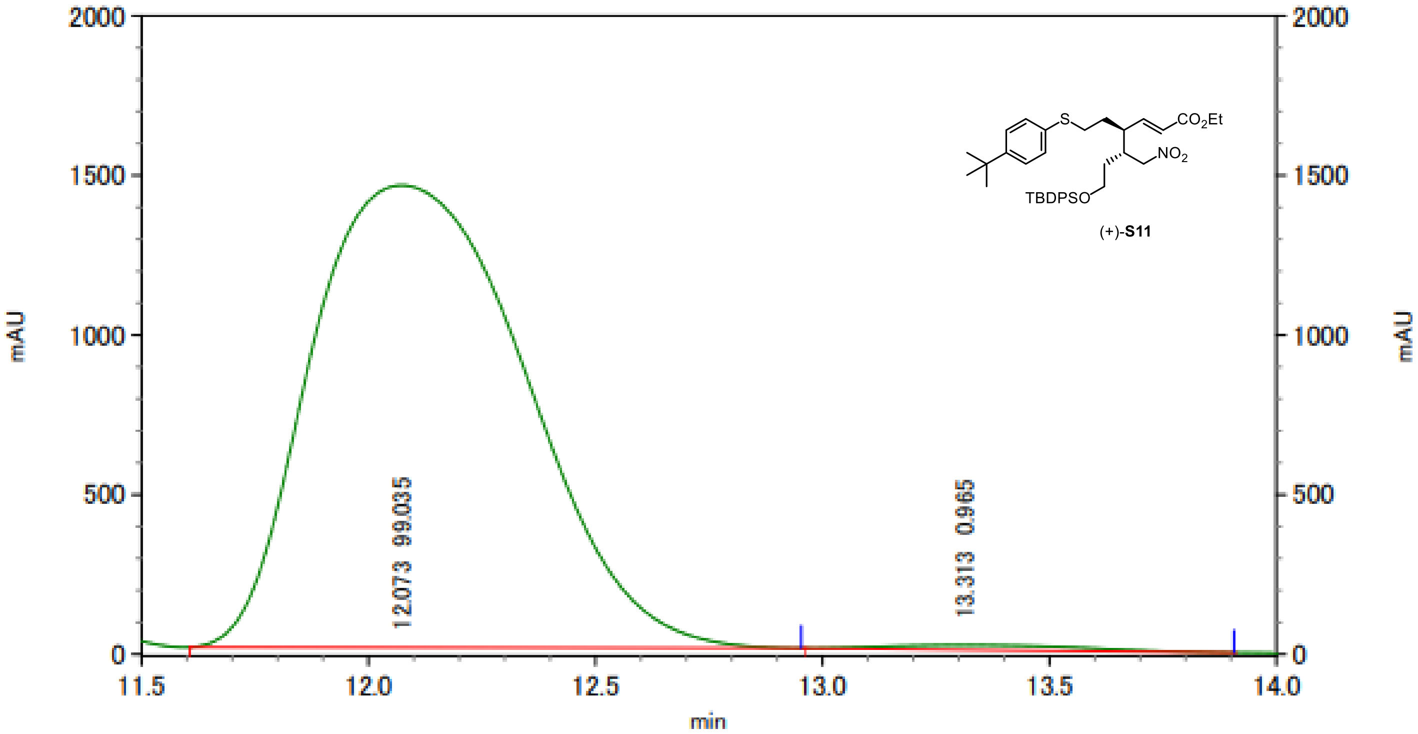

Result
1: 215.0 nm, 4.0 nm 結果

Pk #	Retention time / min	Integration/ %
1	12.073	99.035
2	13.313	0.965
合計		100.000

Tota

Supplementary Figure 20 HPLC trace for the (+)-**S11**

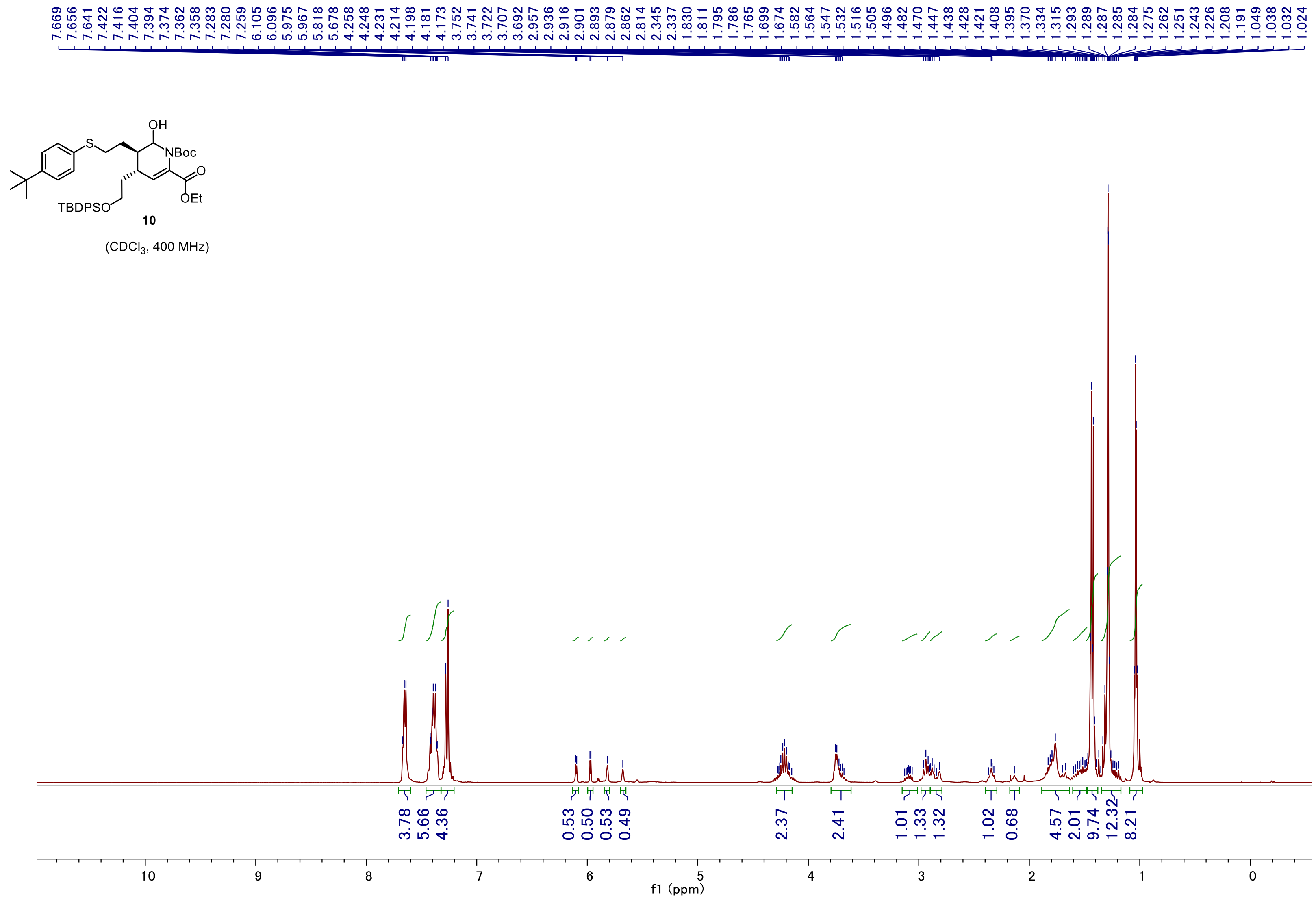

Supplementary Figure 21 ^{1}H NMR (400 MHz, $CDCl_3$) spectra of **10**

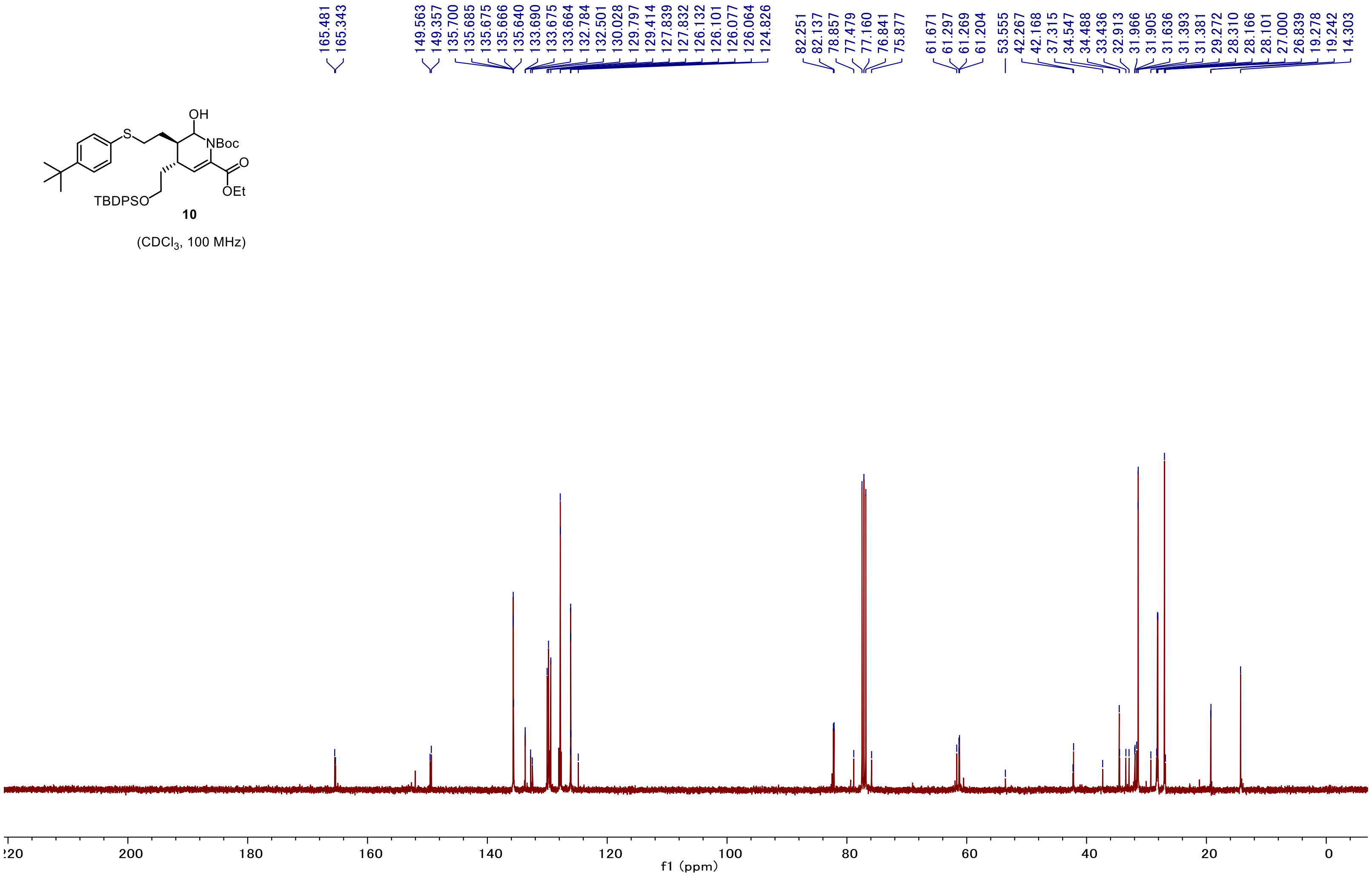

Supplementary Figure 22 ^{13}C NMR (100 MHz, $CDCl_3$) spectra of **10**

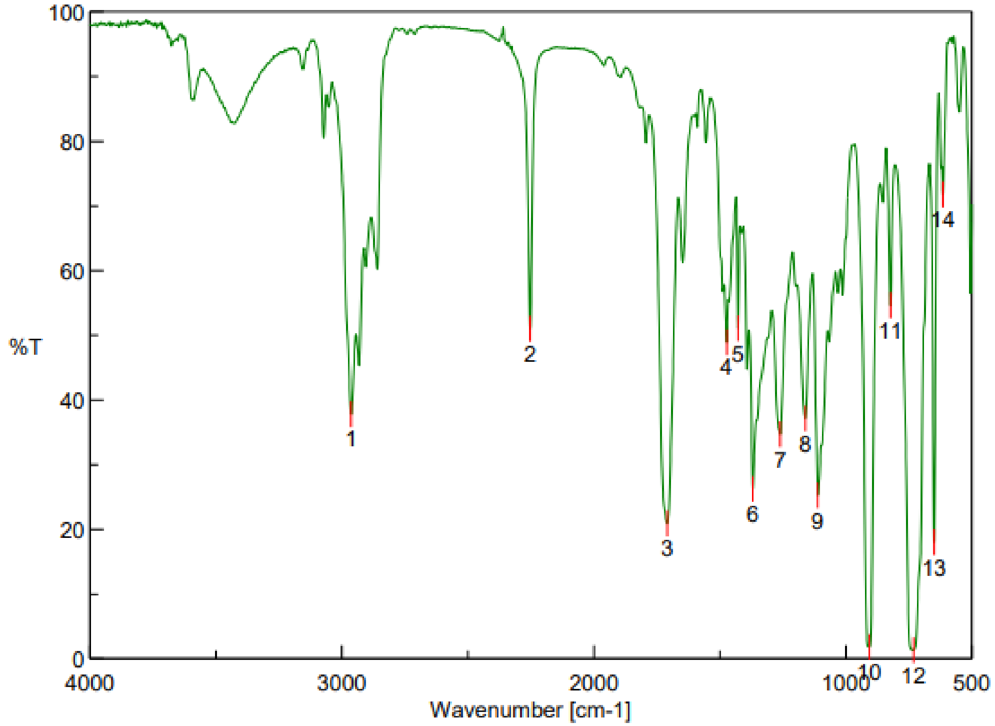

[ピーク検出結果]

No.	位置	強度	No.	位置	強度
1	2964.05	37.7959	2	2253.41	50.9892
3	1708.62	20.9231	4	1473.35	48.9139
5	1428.03	51.0781	6	1370.18	26.238
7	1261.22	34.7088	8	1161.9	37.0997
9	1111.76	25.2563	10	906.379	1.80028
11	823.455	54.6094	12	730.889	1.31151
13	649.893	18.0369	14	615.181	71.7311

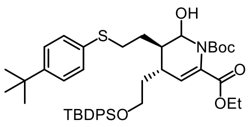

10

Supplementary Figure 23 IR (neat) spectra of **10**

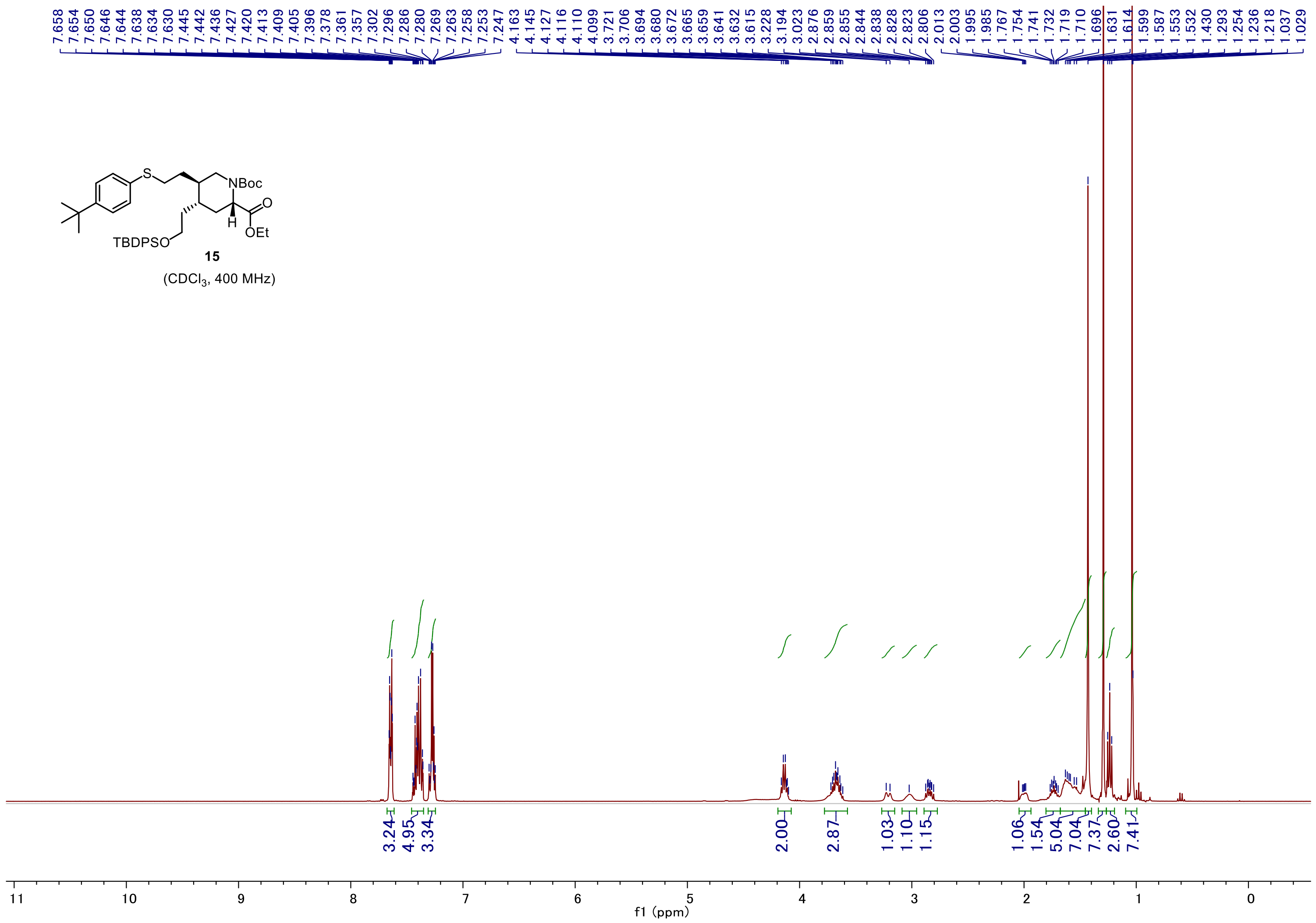

Supplementary Figure 24 ¹H NMR (400 MHz, $CDCl_3$) spectra of **15**

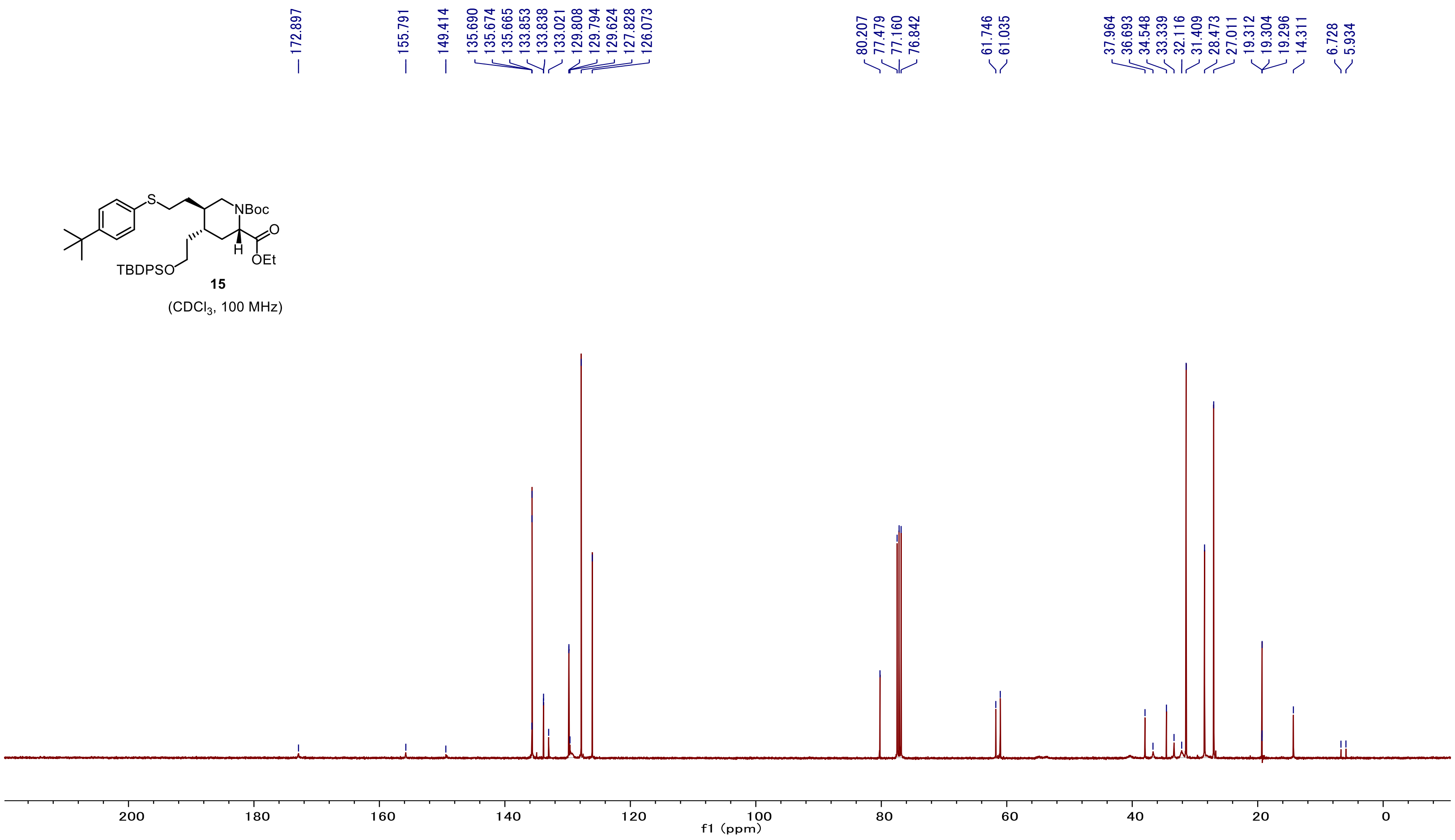

Supplementary Figure 25 ^{13}C NMR (100 MHz, $CDCl_3$) spectra of **15**

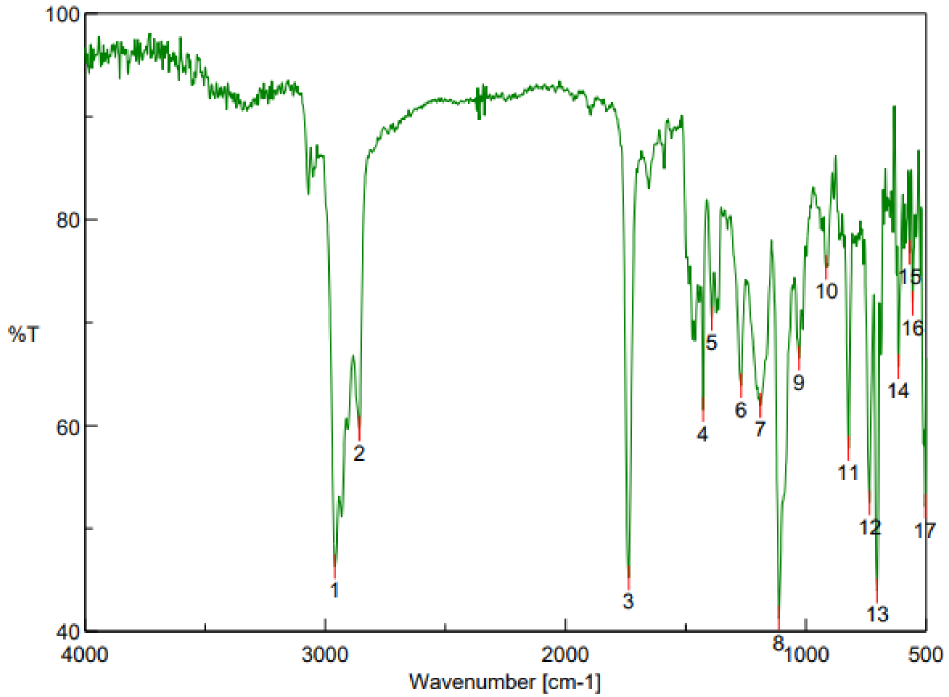

[ピーク検出結果]

No.	位置	強度	No.	位置	強度
1	2959.23	46.301	2	2857.99	59.6866
3	1737.55	45.194	4	1428.03	61.5281
5	1391.39	70.3973	6	1269.9	63.8781
7	1188.9	61.9525	8	1111.76	41.2729
9	1028.84	66.5053	10	915.058	75.3277
11	822.491	57.7726	12	734.746	52.4836
13	702.926	43.9475	14	614.217	65.7225
15	567.934	76.8123	16	555.398	71.8664
17	503.33	52.1401			

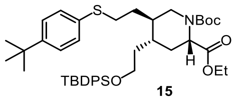

15

Supplementary Figure 26 IR (neat) spectra of **15**

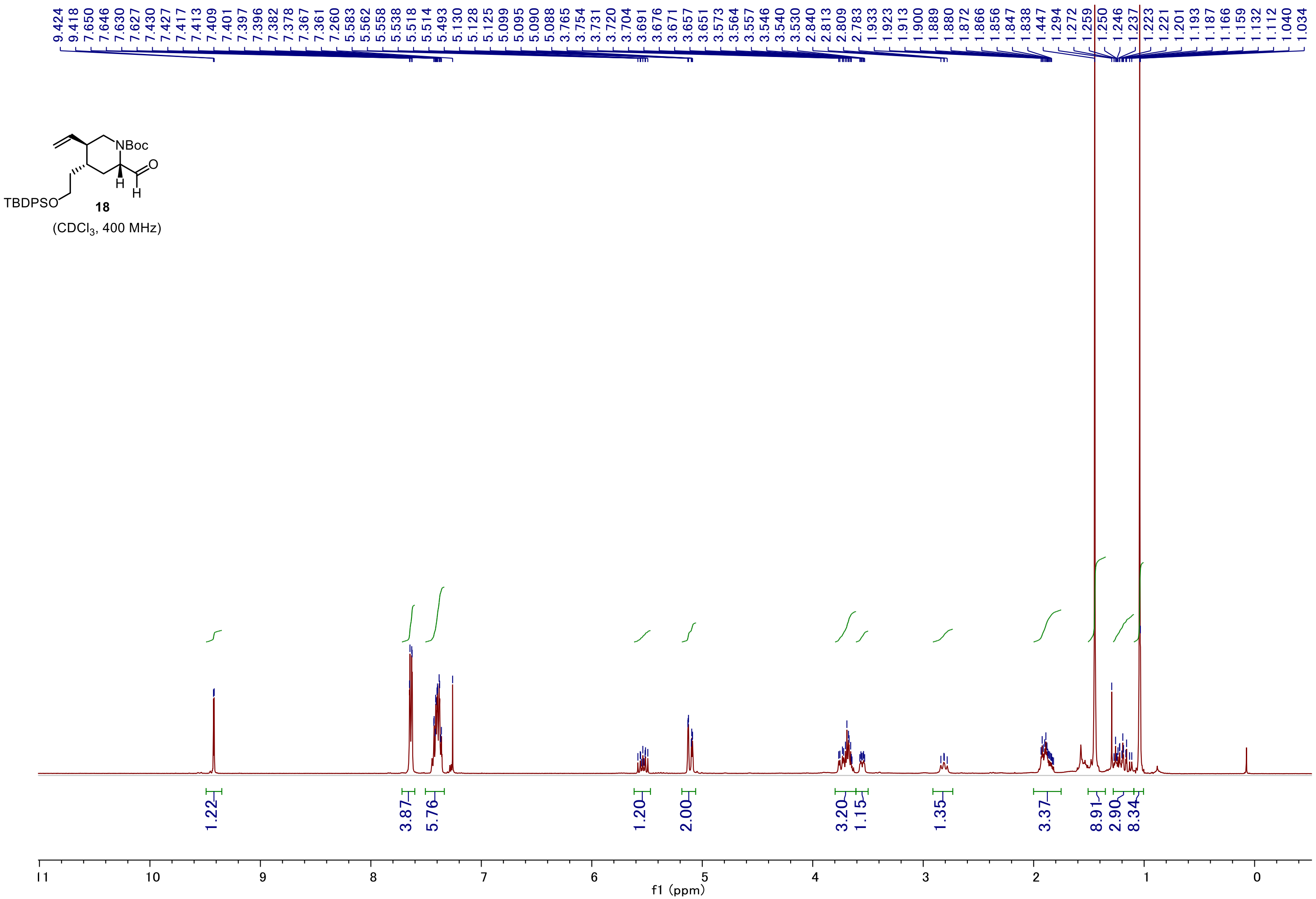

Supplementary Figure 27 ^{1}H NMR (400 MHz, $CDCl_3$) spectra of **18**

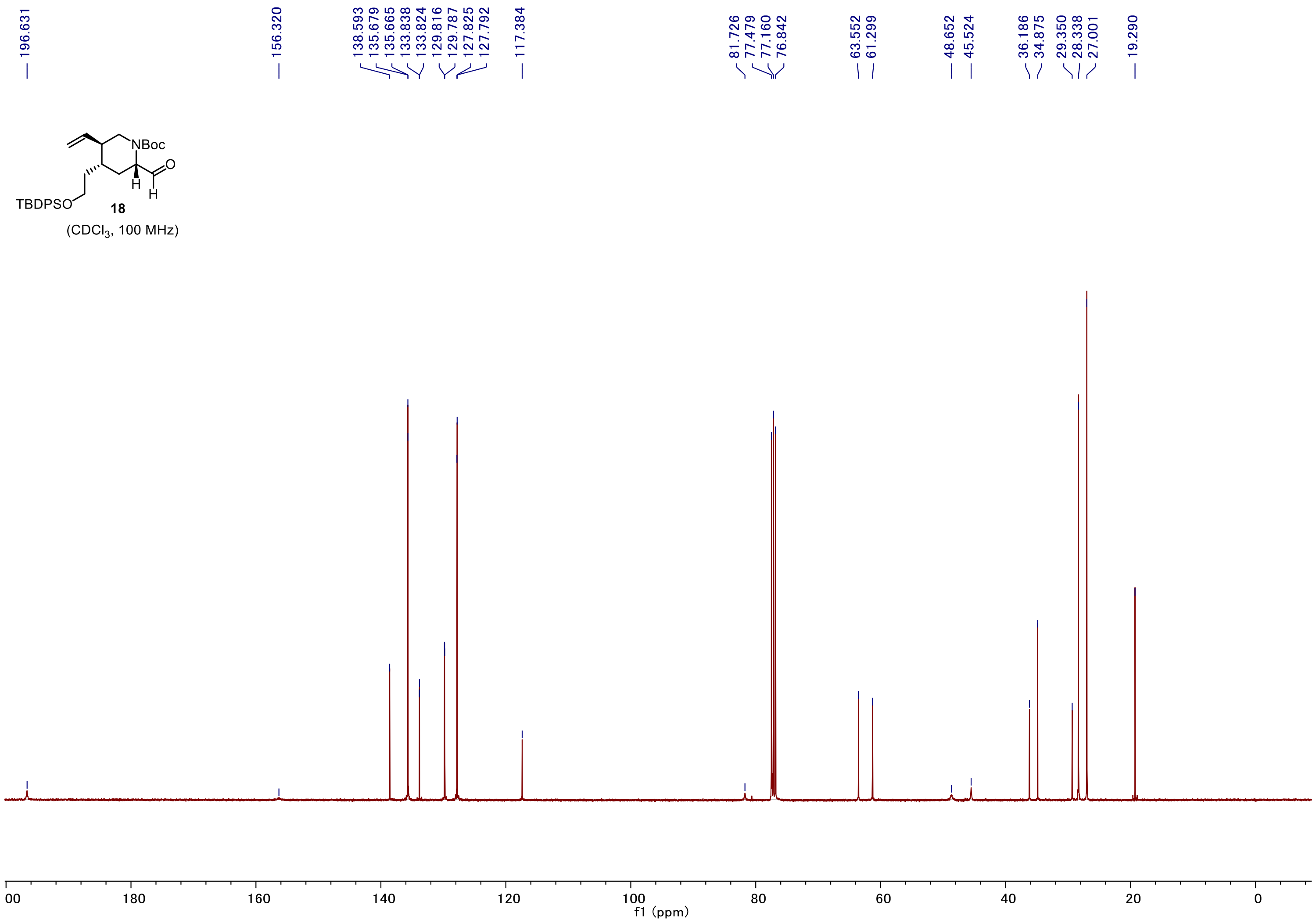

Supplementary Figure 28 ^{13}C NMR (100 MHz, $CDCl_3$) spectra of **18**

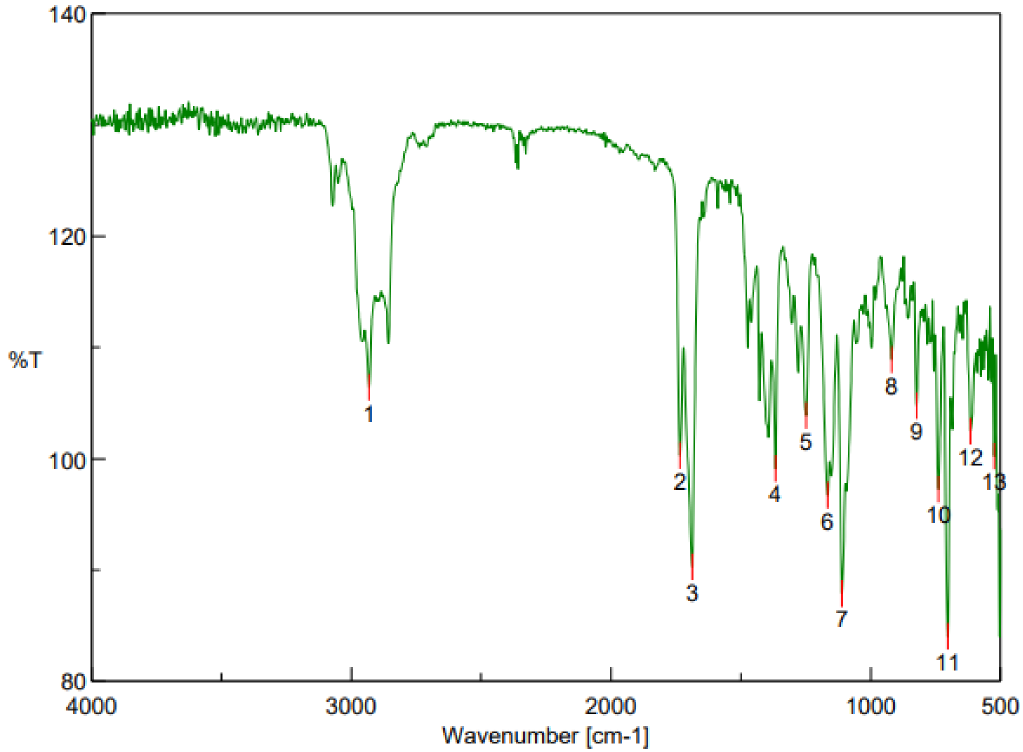

[ピーク検出結果]

No.	位置	強度	No.	位置	強度
1	2931.27	106.393	2	1733.69	100.269
3	1687.41	90.2574	4	1367.28	99.0964
5	1247.72	103.863	6	1165.76	96.7057
7	1110.8	87.8746	8	918.914	108.894
9	823.455	104.738	10	738.603	97.2589
11	702.926	84.0253	12	615.181	102.457
13	523.579	100.227			

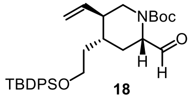

18

Supplementary Figure 29 IR (neat) spectra of **18**

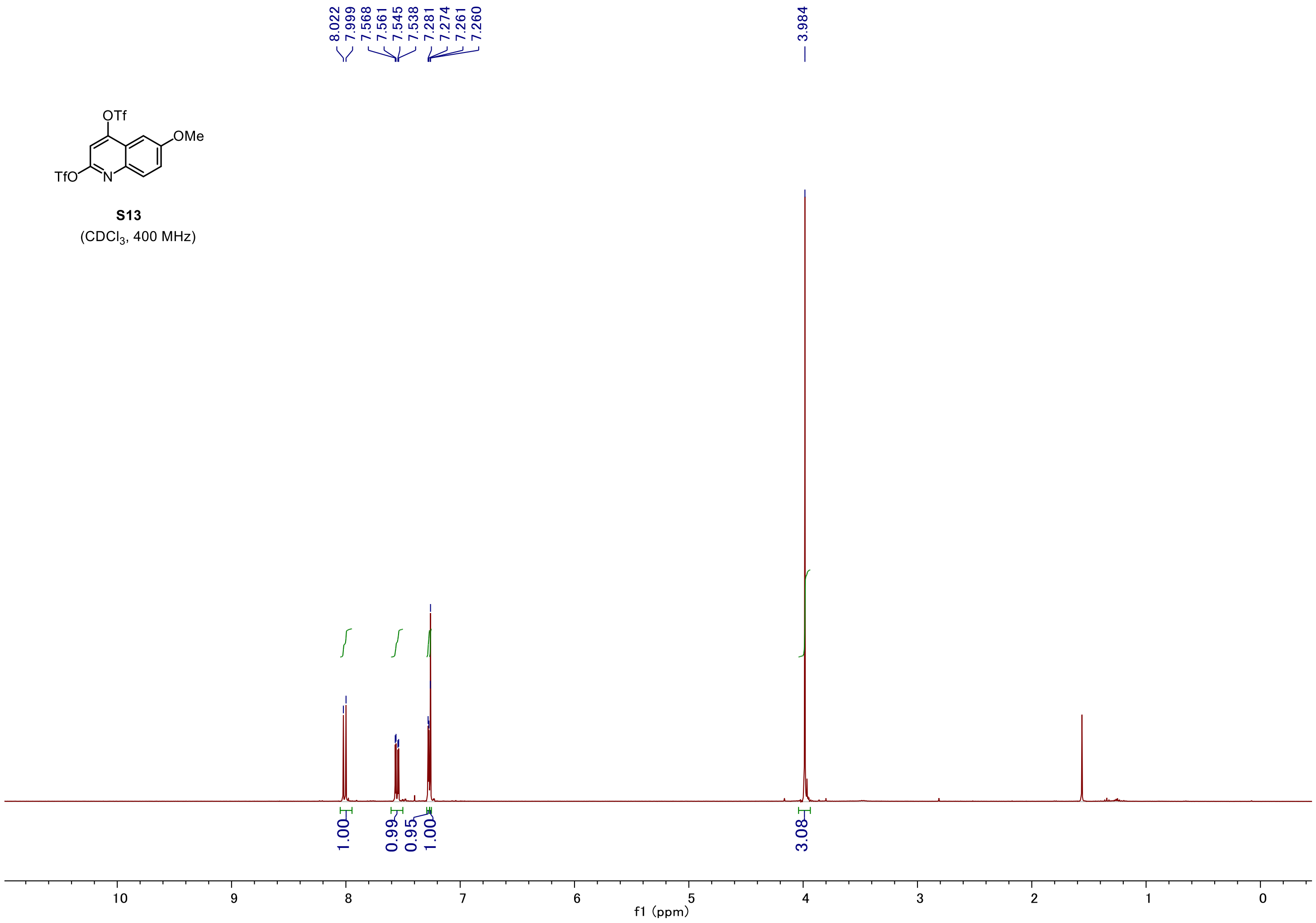

Supplementary Figure 30 ^{1}H NMR (400 MHz, $CDCl_3$) spectra of **S13**

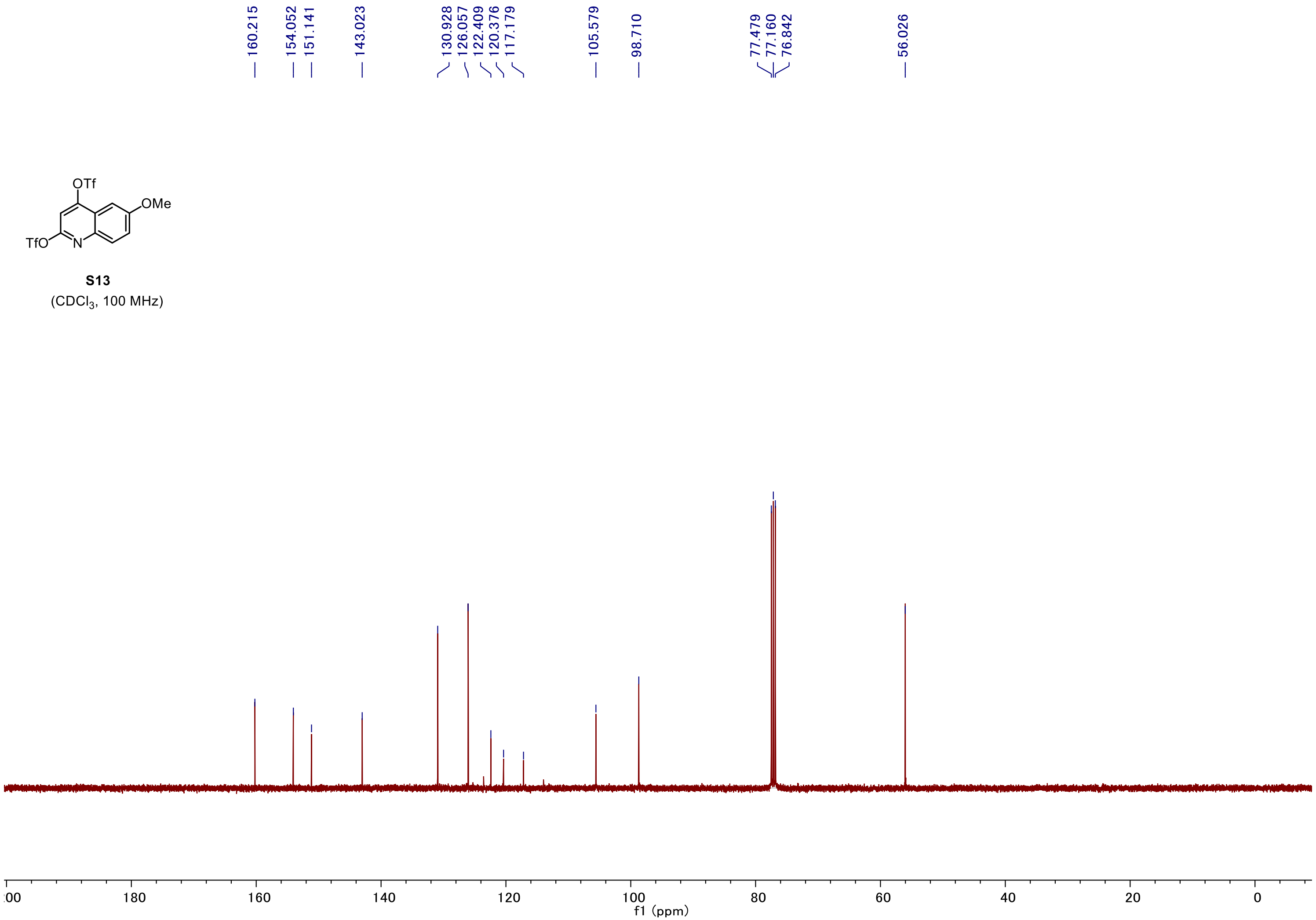

Supplementary Figure 31 ^{13}C NMR (100 MHz, $CDCl_3$) spectra of **S13**

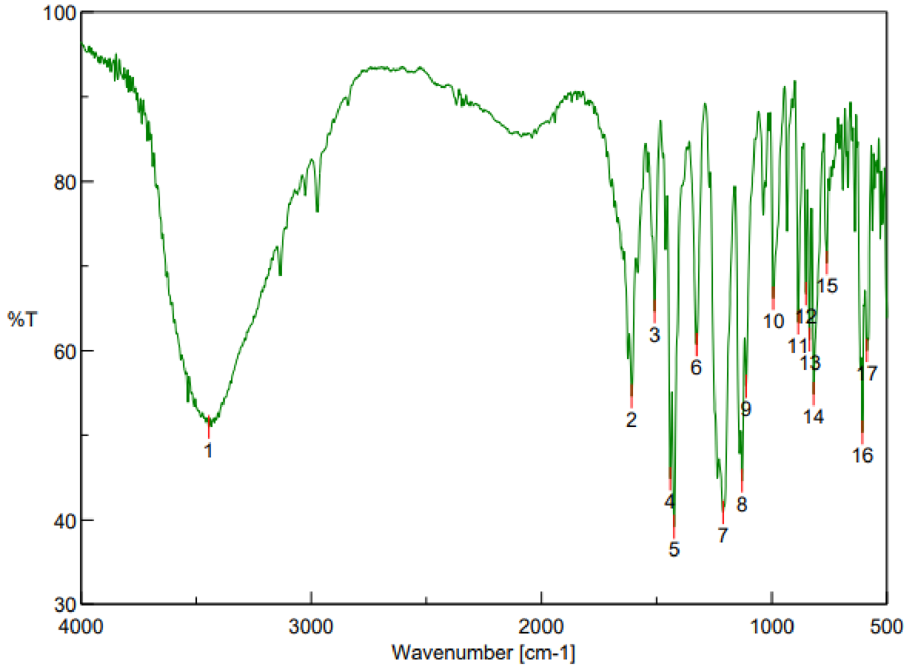

[ピーク検出結果]

No.	位置	強度	No.	位置	強度
1	3445.21	50.9575	2	1608.34	54.5896
3	1508.06	64.6041	4	1440.56	44.8372
5	1423.21	39.1476	6	1326.79	60.7112
7	1212.04	40.8633	8	1129.12	44.5971
9	1110.8	55.758	10	993.16	66.1544
11	885.166	63.2955	12	851.418	66.7021
13	835.026	61.2952	14	818.634	54.8554
15	759.816	70.3575	16	605.539	50.3072
17	586.254	60.0075			

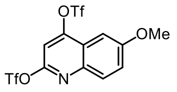

S13

Supplementary Figure 32 IR (neat) spectra of **S13**

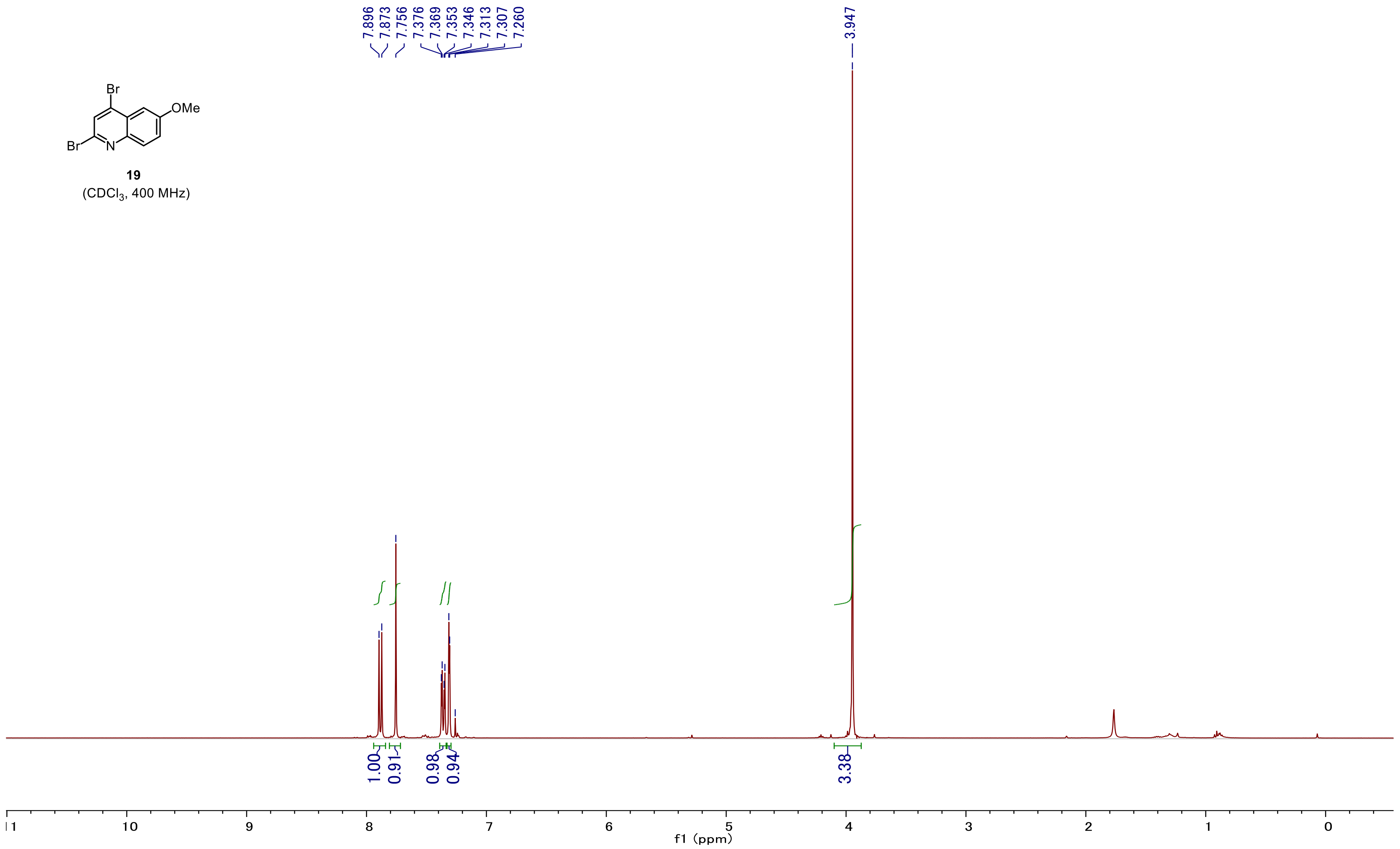

Supplementary Figure 33 ^{1}H NMR (400 MHz, $CDCl_3$) spectra of **19**

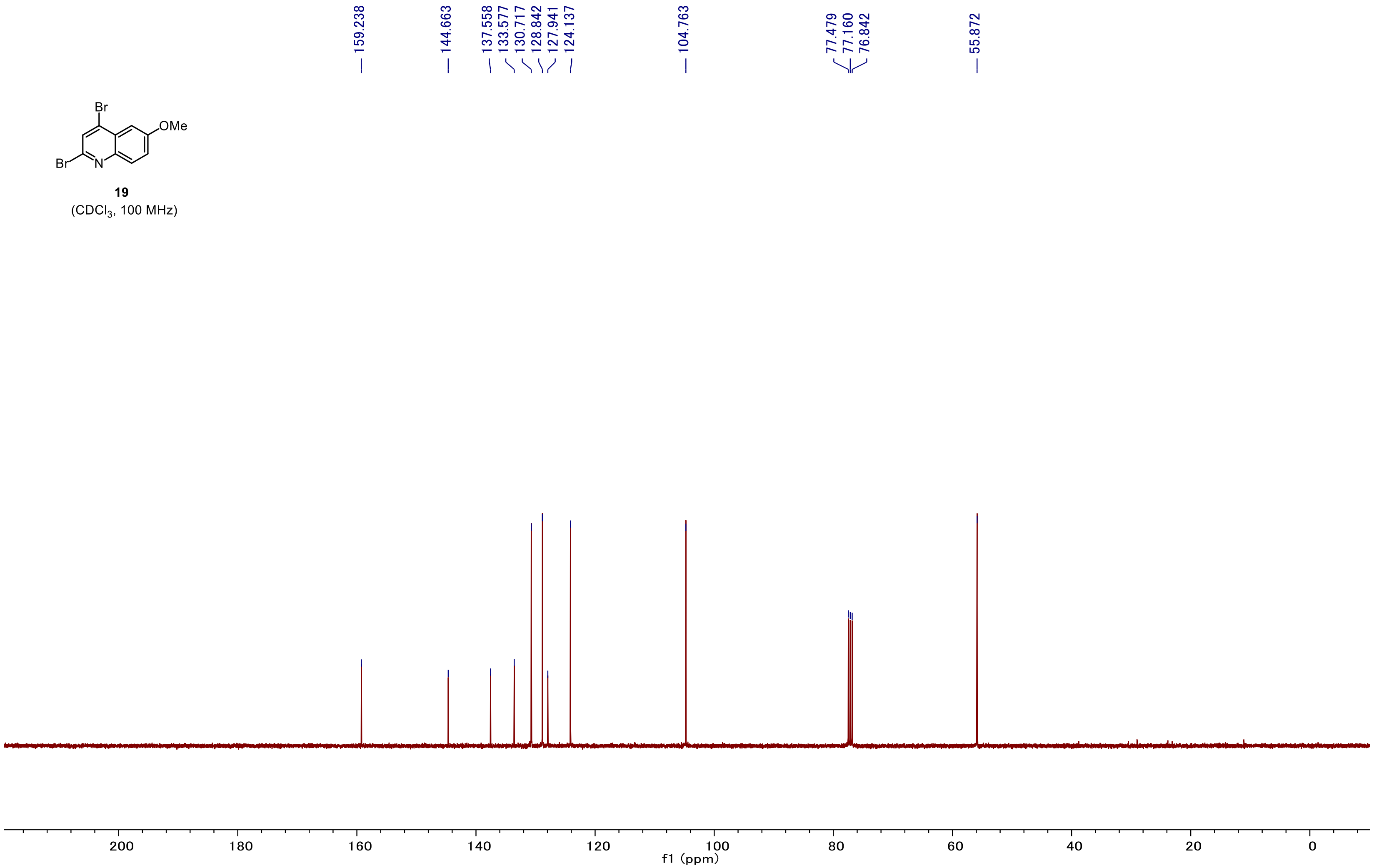

Supplementary Figure 34 ^{13}C NMR (100 MHz, $CDCl_3$) spectra of **19**

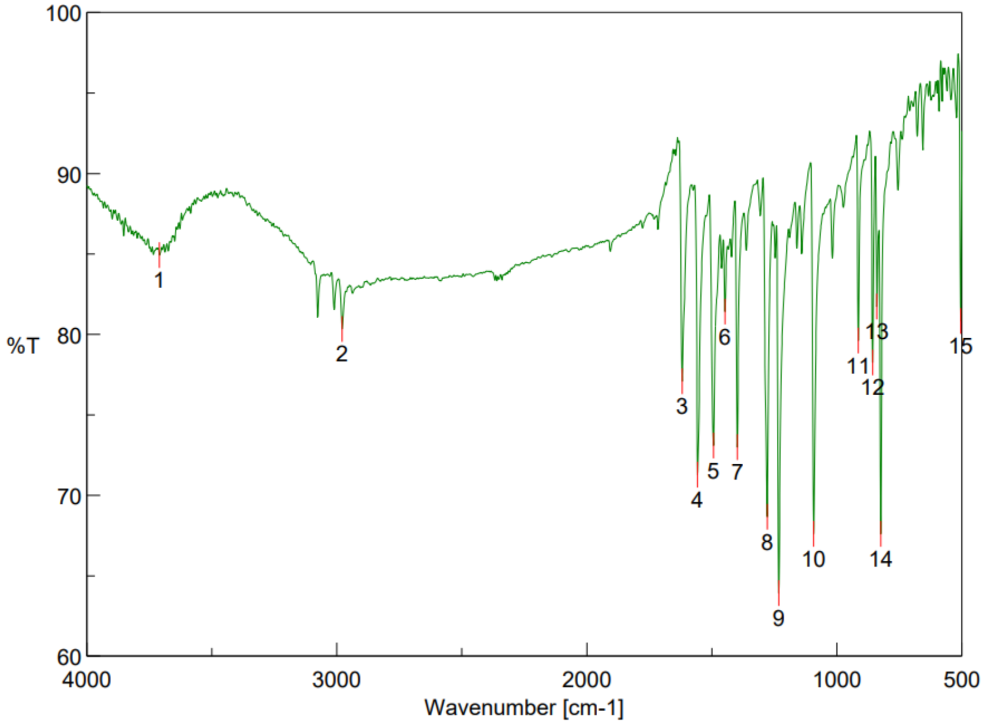

[ピーク検出結果]

No.	位置	強度	No.	位置	強度
1	3709.41	84.9131	2	2978.52	80.3236
3	1618.95	77.0859	4	1557.24	71.2683
5	1493.6	73.0854	6	1447.31	81.4006
7	1398.14	72.9882	8	1278.57	68.6514
9	1232.29	63.9186	10	1092.48	67.59
11	914.093	79.6167	12	856.239	78.2443
13	839.847	81.7268	14	824.42	67.5839
15	504.294	80.8256			

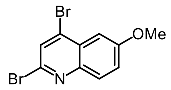

19

Supplementary Figure 35 IR (neat) spectra of **19**

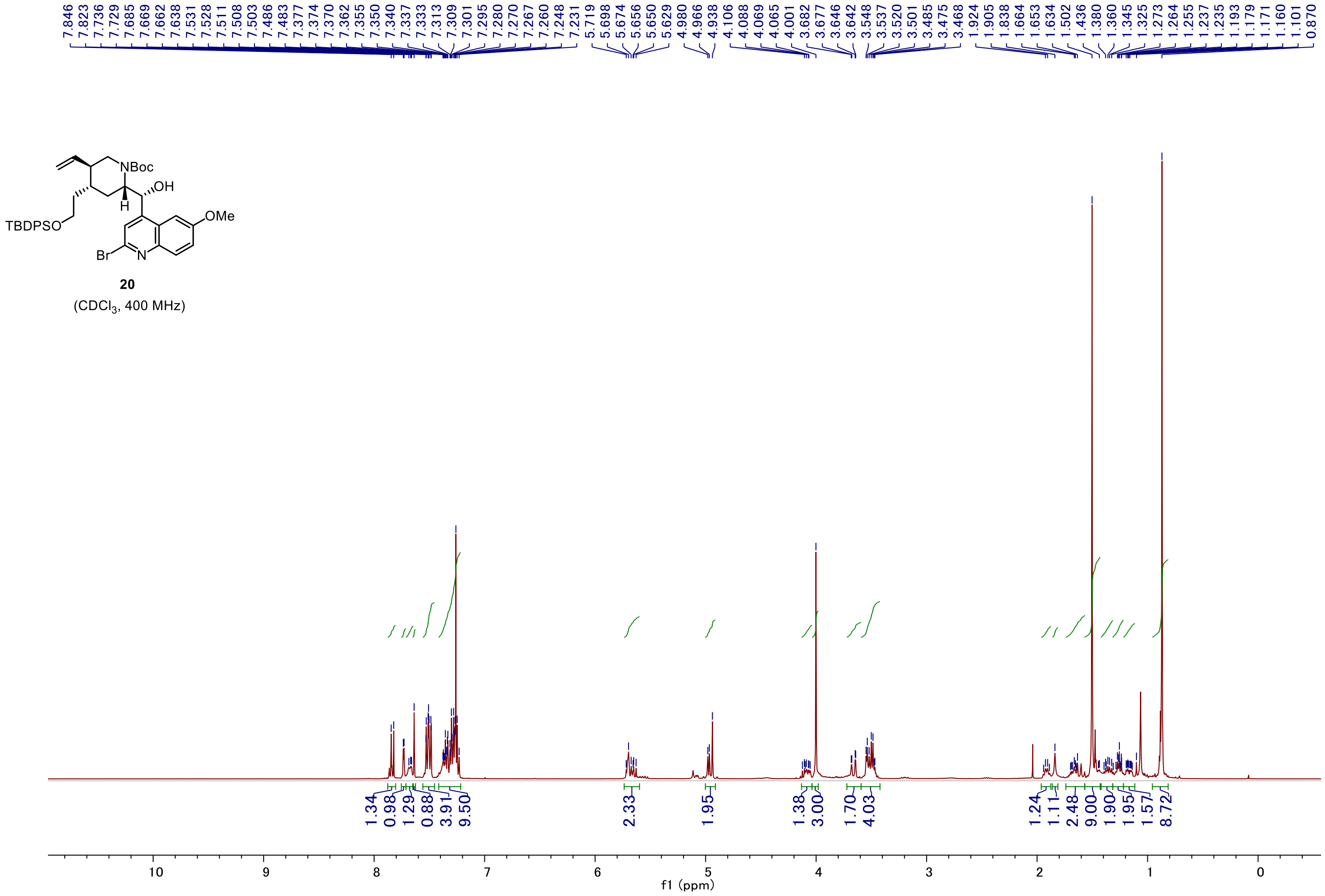

Supplementary Figure 36 ^{1}H NMR (400 MHz, $CDCl_3$) spectra of **20**

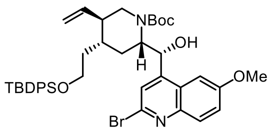

20
($CDCl_3$, 100 MHz)

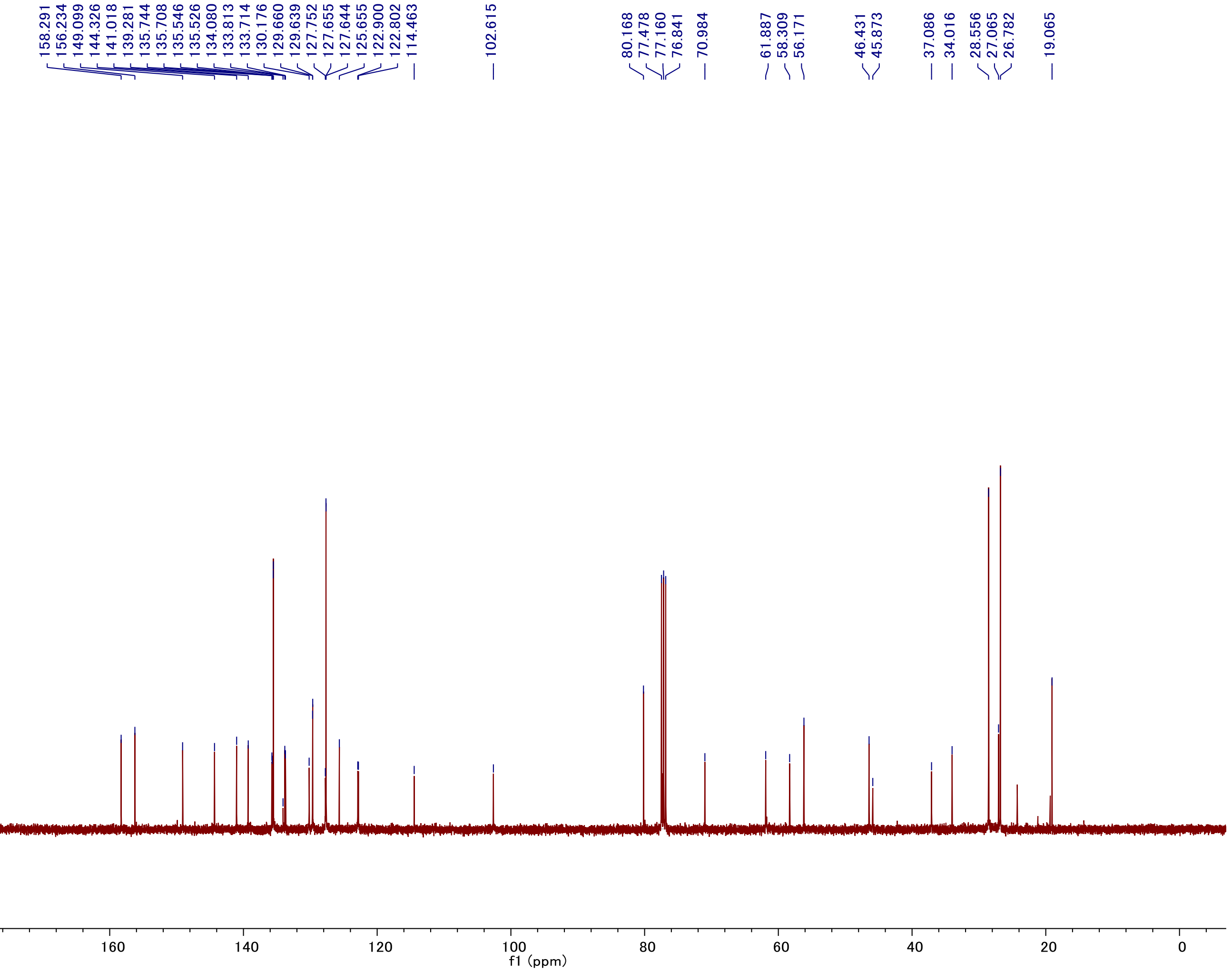

Supplementary Figure 37 ^{13}C NMR (100 MHz, $CDCl_3$) spectra of **20**

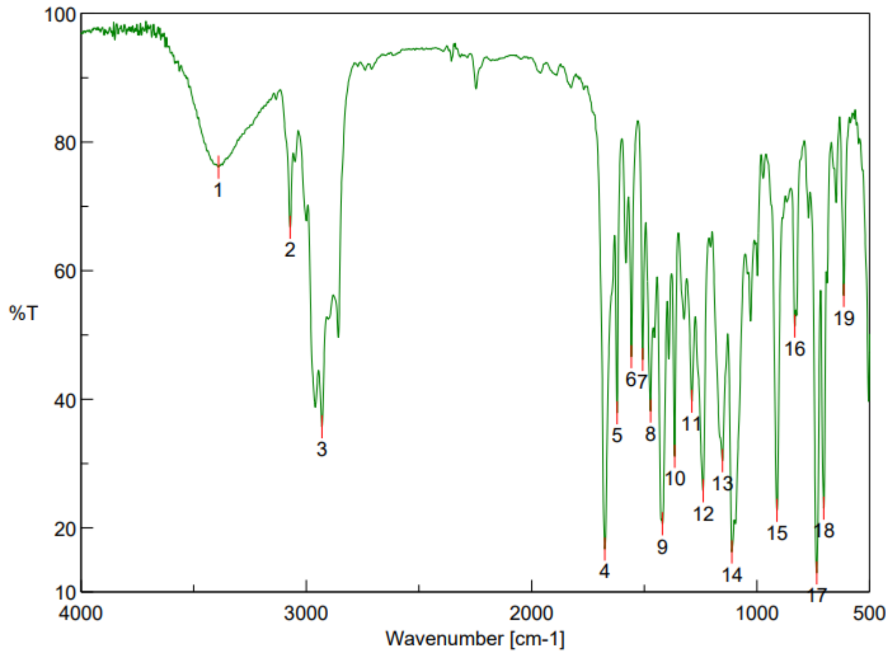

[ピーク検出結果]

No.	位置	強度	No.	位置	強度
1	3390.24	76.0634	2	3072.05	66.687
3	2930.31	35.7045	4	1674.87	16.6723
5	1620.88	37.8834	6	1557.24	46.5757
7	1507.1	46.1589	8	1472.38	38.1129
9	1419.35	20.6133	10	1365.35	31.099
11	1289.18	39.67	12	1239.04	25.7312
13	1153.22	30.4015	14	1110.8	16.2159
15	911.201	22.7152	16	832.133	51.3598
17	734.746	12.9427	18	702.926	23.0362
19	614.217	56.091			

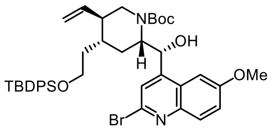

20

Supplementary Figure 38 IR (neat) spectra of **21**

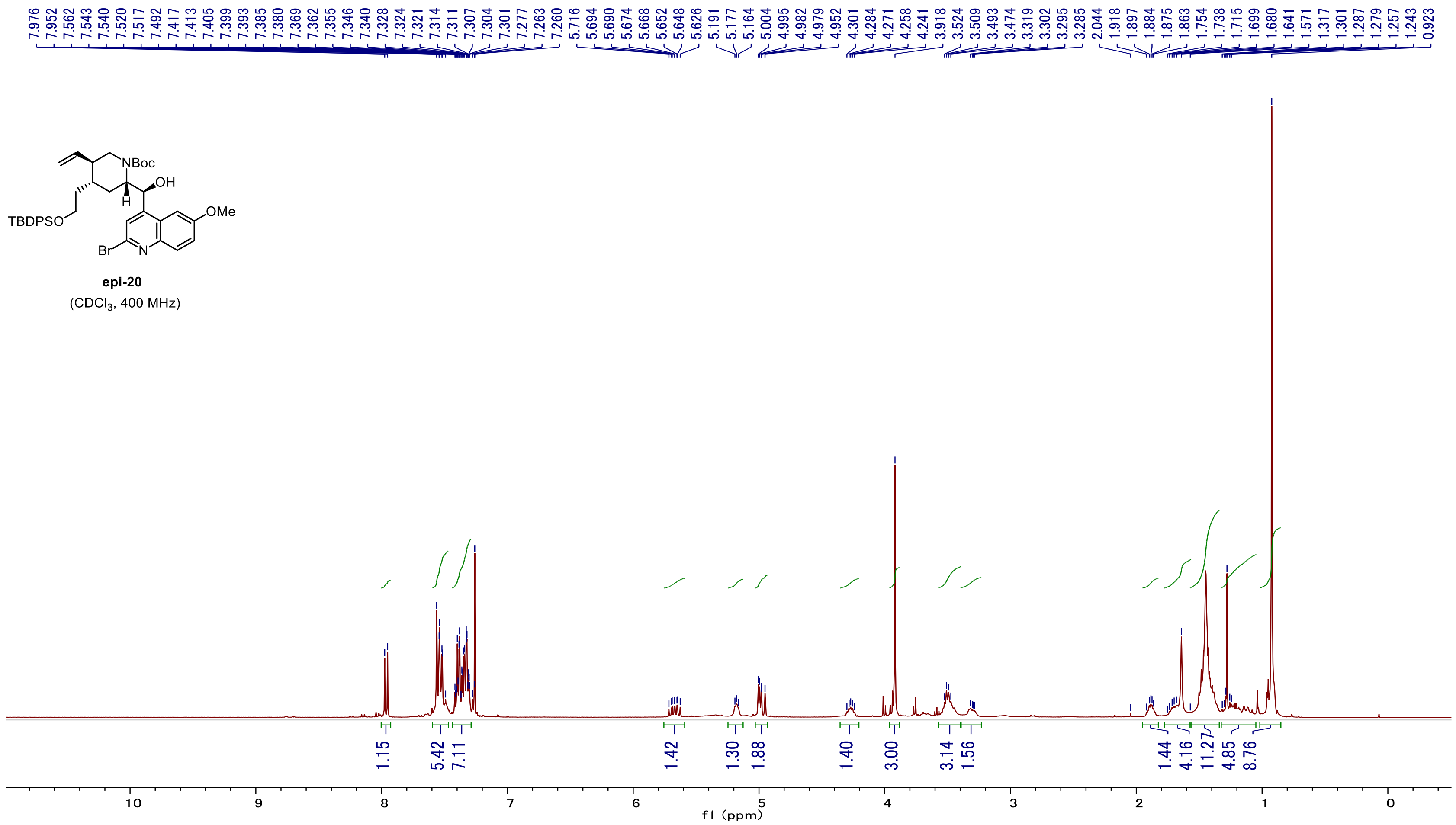

Supplementary Figure 39 ^{1}H NMR (400 MHz, $CDCl_3$) spectra of **epi-20**

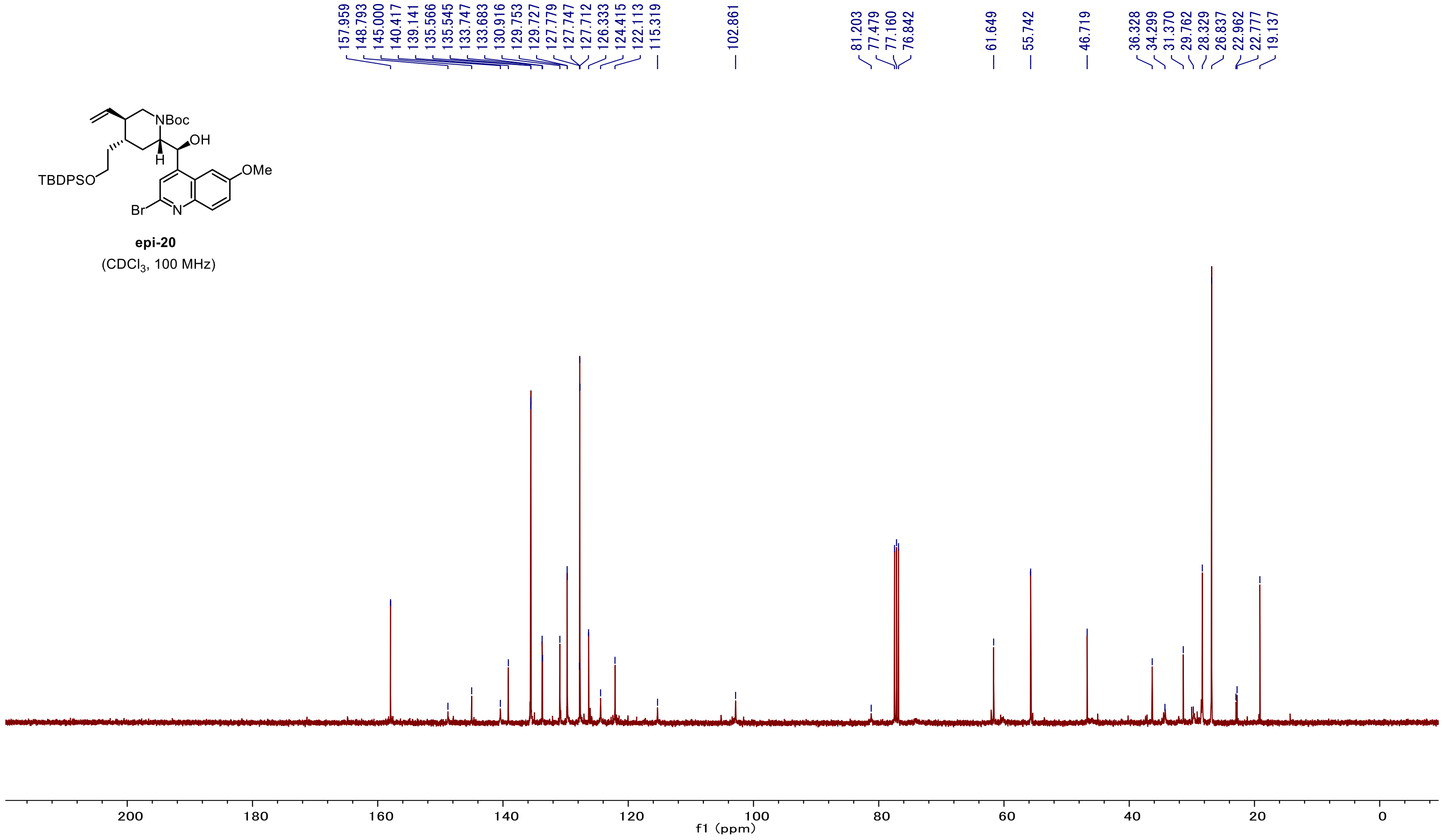

Supplementary Figure 40 ^{13}C NMR (100 MHz, $CDCl_3$) spectra of **epi-20**

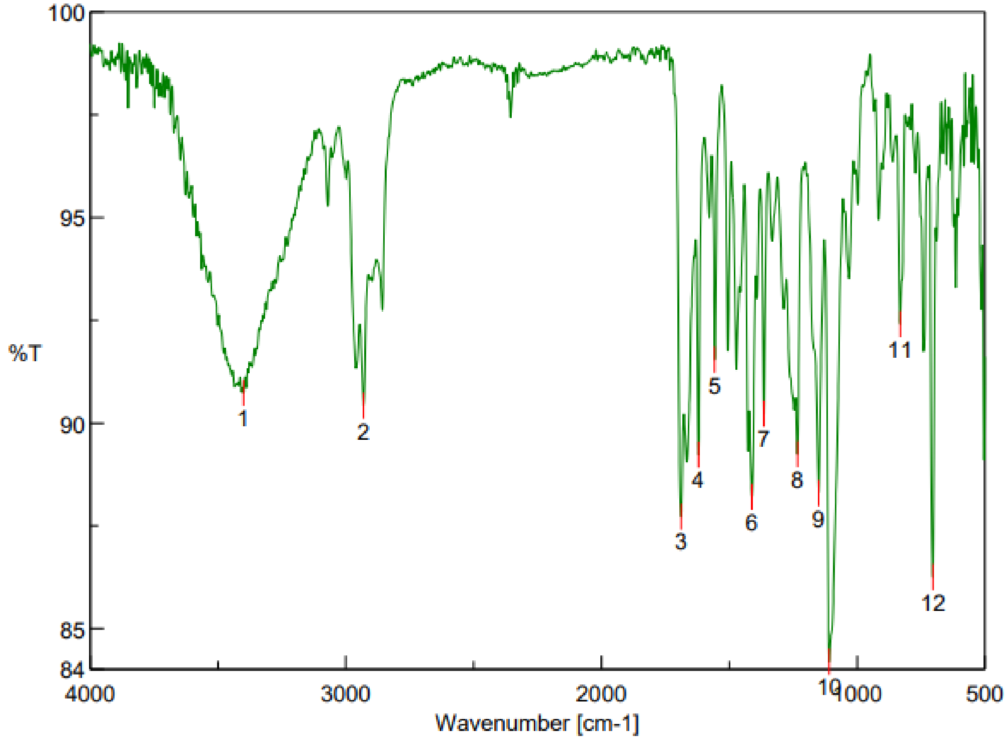

[ピーク検出結果]

No.	位置	強度	No.	位置	強度
1	3400.85	90.7332	2	2930.31	90.4171
3	1688.37	87.7186	4	1620.88	89.2249
5	1557.24	91.54	6	1411.64	88.1961
7	1364.39	90.2288	8	1233.25	89.2386
9	1150.33	88.2877	10	1108.87	84.1871
11	831.169	92.4028	12	703.89	86.242

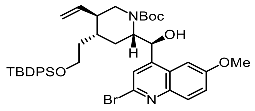

epi-20

Supplementary Figure 41 IR (neat) spectra of **epi-20**

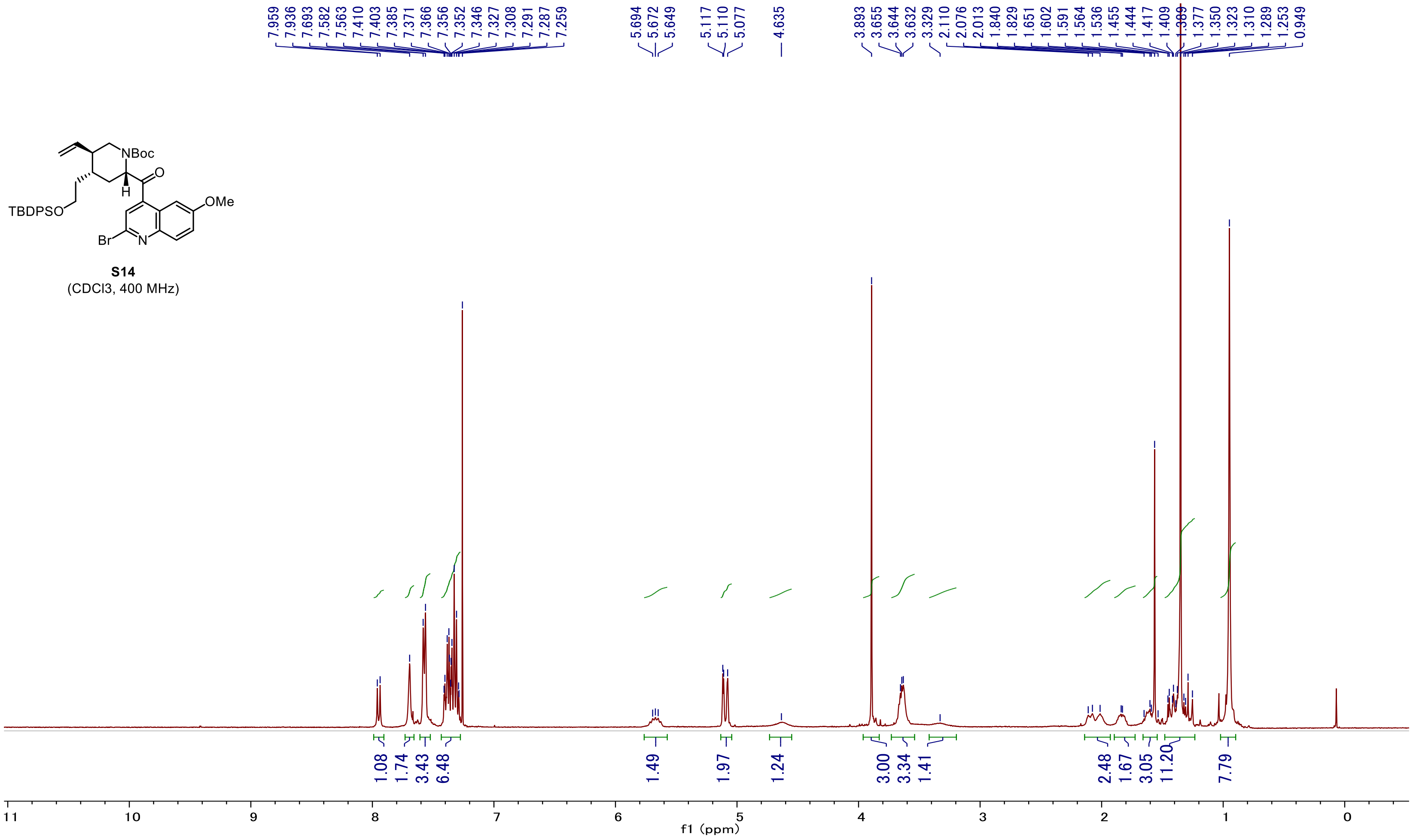

Supplementary Figure 42 ¹H NMR (400 MHz, $CDCl_3$) spectra of **S14**

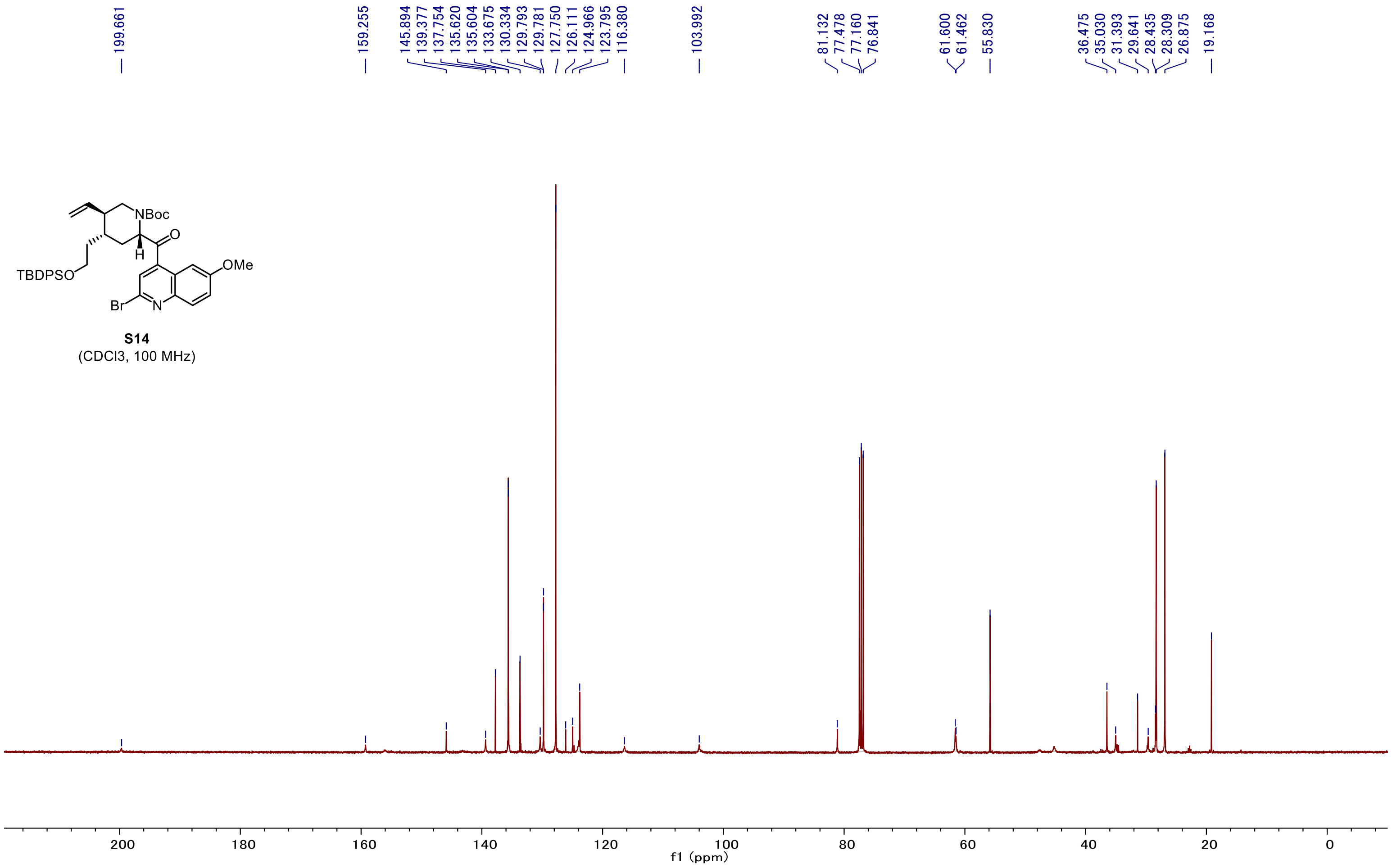

Supplementary Figure 43 ^{13}C NMR (100 MHz, $CDCl_3$) spectra of **S14**

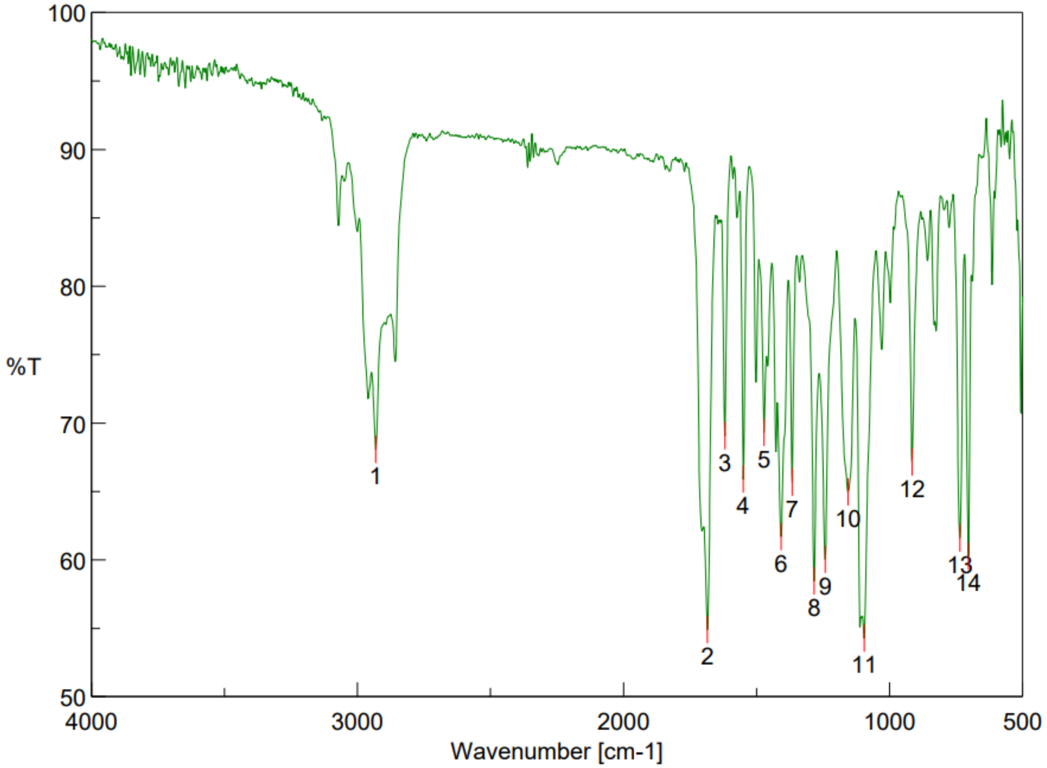

[ピーク検出結果]

No.	位置	強度	No.	位置	強度
1	2931.27	68.0656	2	1684.52	54.8925
3	1618.95	69.0573	4	1549.52	65.9071
5	1471.42	69.3171	6	1407.78	61.6943
7	1366.32	65.6614	8	1283.39	58.4448
9	1241.93	60.035	10	1156.12	64.9607
11	1095.37	54.2689	12	915.058	67.1468
13	735.71	61.5996	14	702.926	60.2493

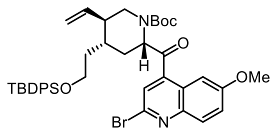

S14

Supplementary Figure 44 IR (neat) spectra of **S14**

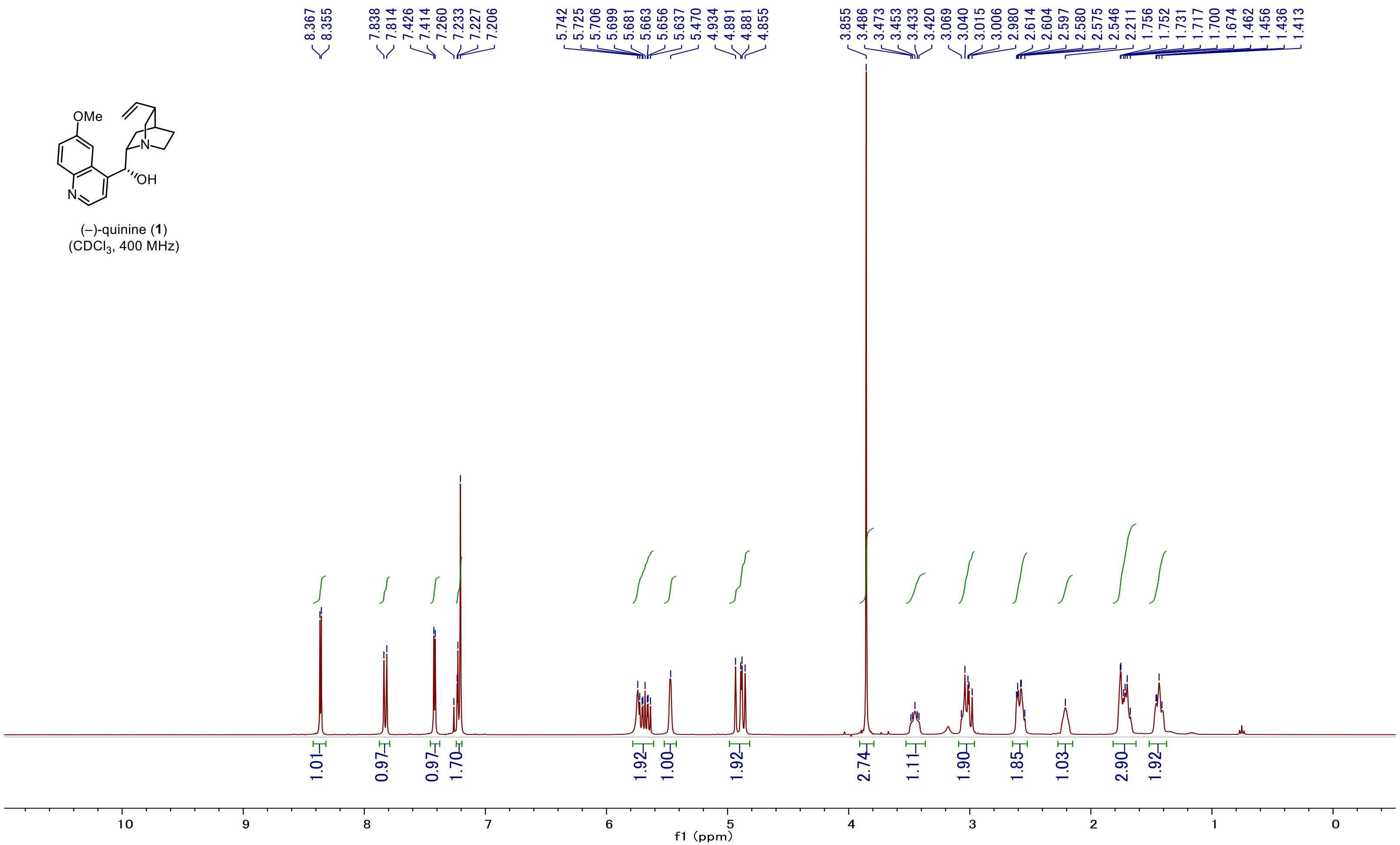

Supplementary Figure 45 ^{1}H NMR (400 MHz, $CDCl_3$) spectra of **1**

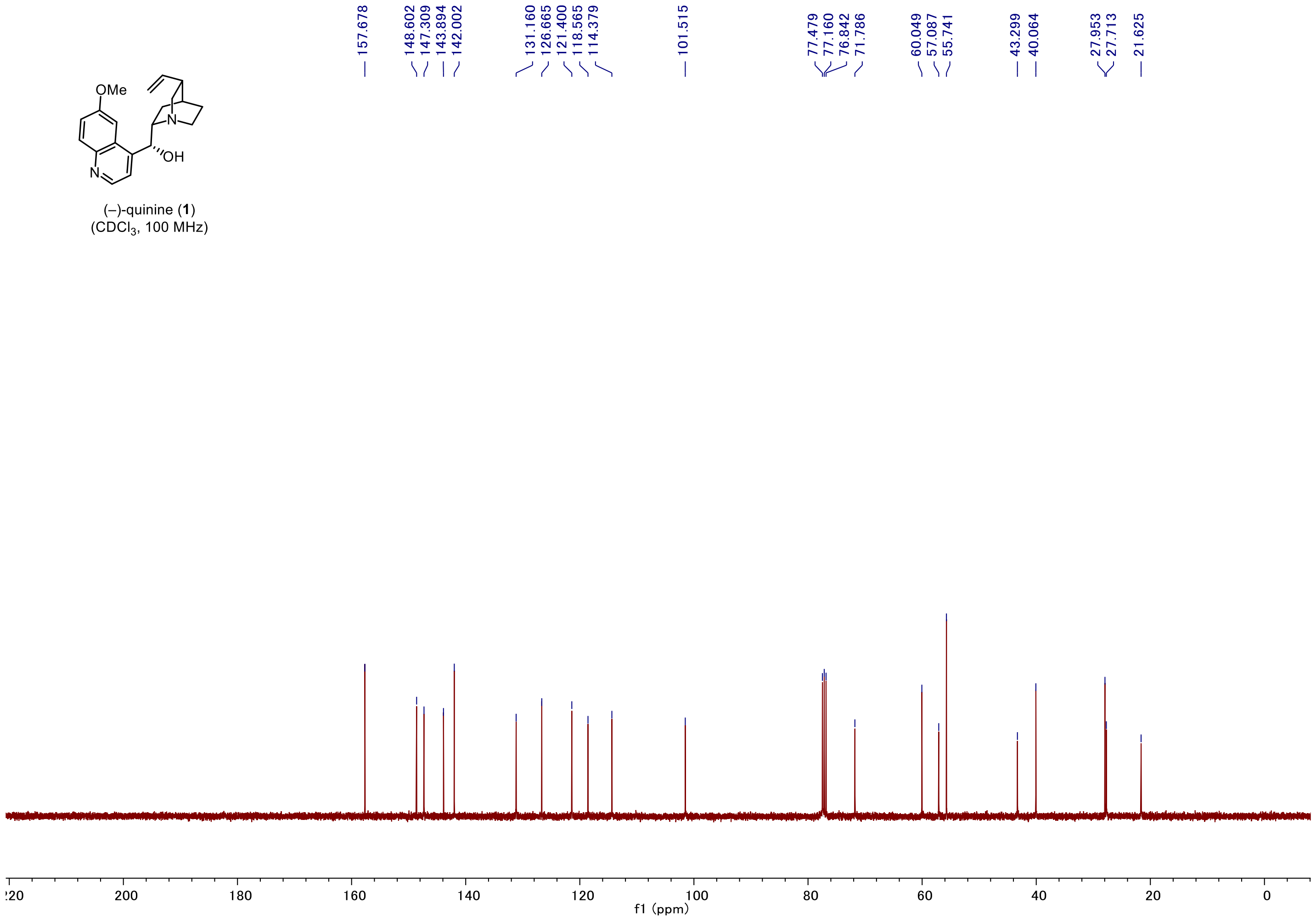

Supplementary Figure 46 ^{13}C NMR (100 MHz, $CDCl_3$) spectra of **1**

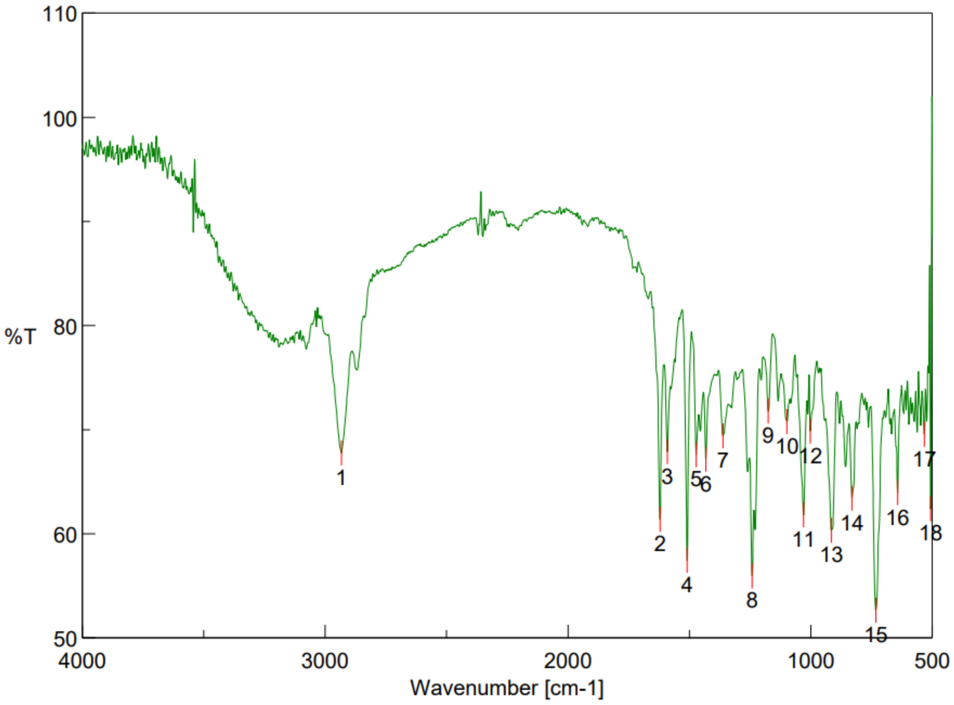

[ピーク検出結果]

No.	位置	強度	No.	位置	強度
1	2933.2	67.7649	2	1620.88	61.3809
3	1590.99	67.8687	4	1509.03	57.4291
5	1472.38	67.5859	6	1431.89	67.1426
7	1361.5	69.4054	8	1241.93	55.938
9	1175.4	71.783	10	1098.26	70.7649
11	1029.8	61.8216	12	1001.84	69.8403
13	915.058	60.3201	14	830.205	63.4156
15	731.853	52.6473	16	642.179	63.9365
17	532.257	69.557	18	506.223	62.3793

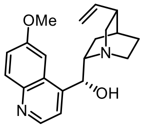

(–)-quinine (**1**)

Supplementary Figure 47 IR (neat) spectra of **1**

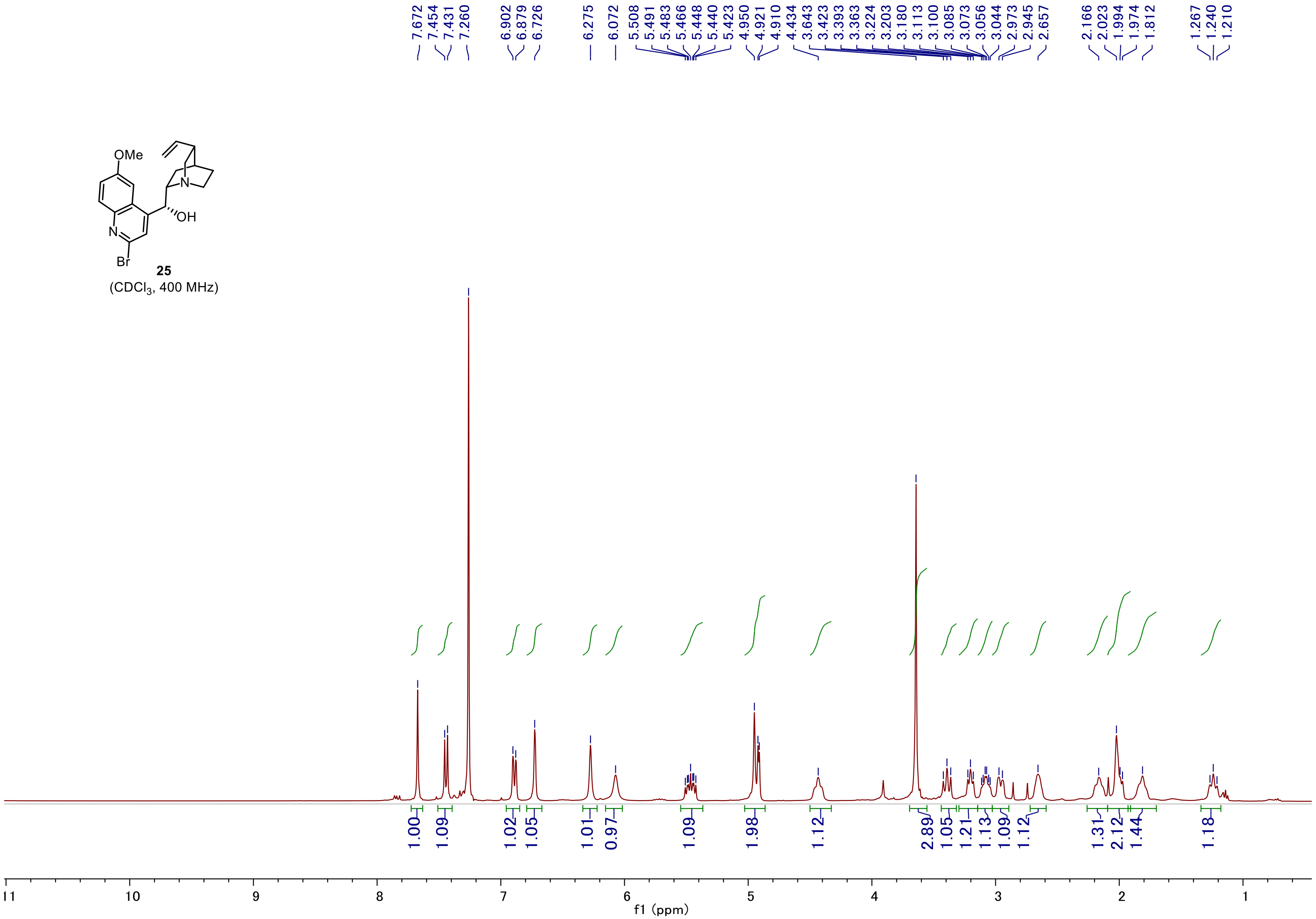

Supplementary Figure 48 ^{1}H NMR (400 MHz, $CDCl_3$) spectra of **25**

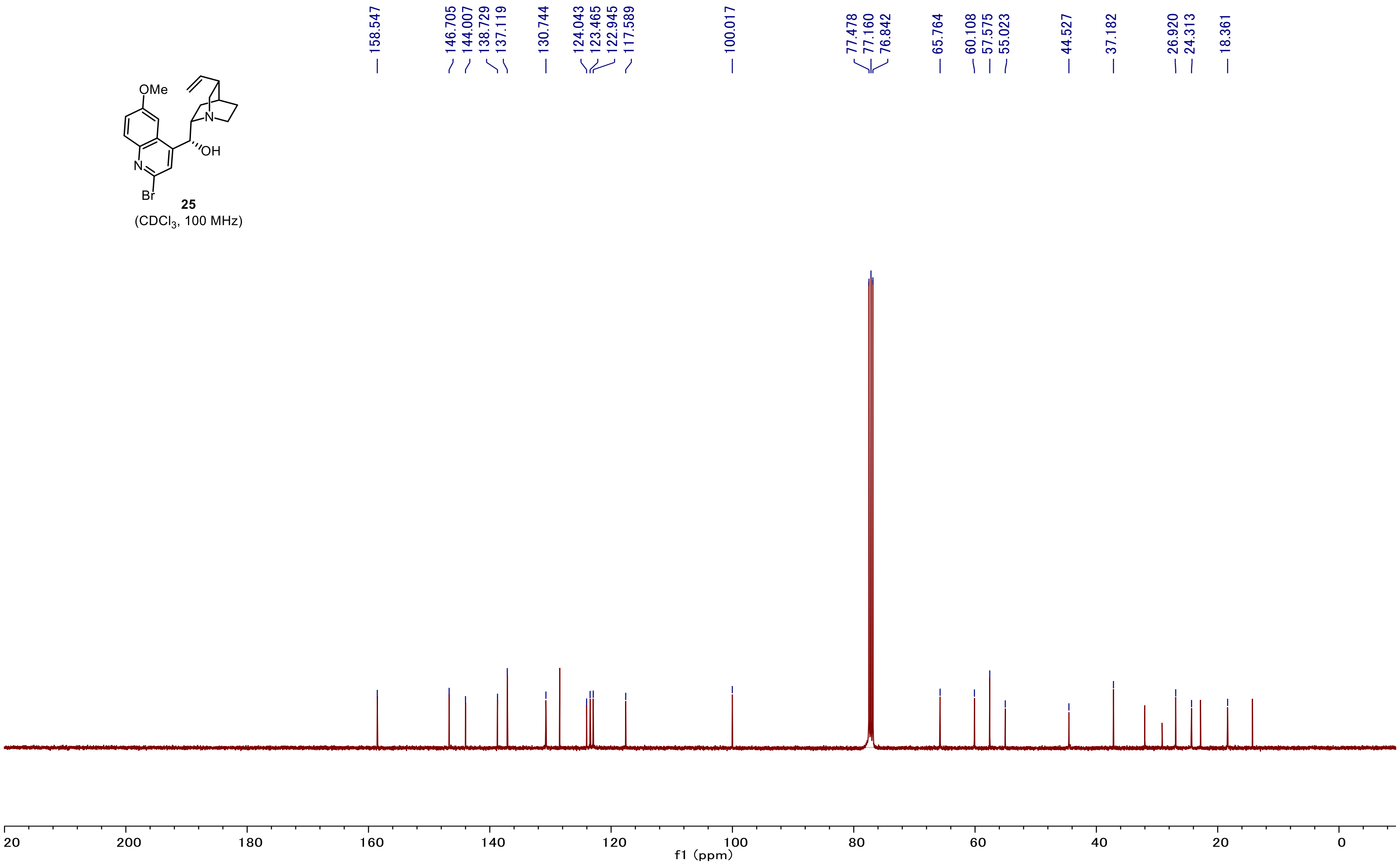

Supplementary Figure 49 ^{13}C NMR (100 MHz, $CDCl_3$) spectra of **25**

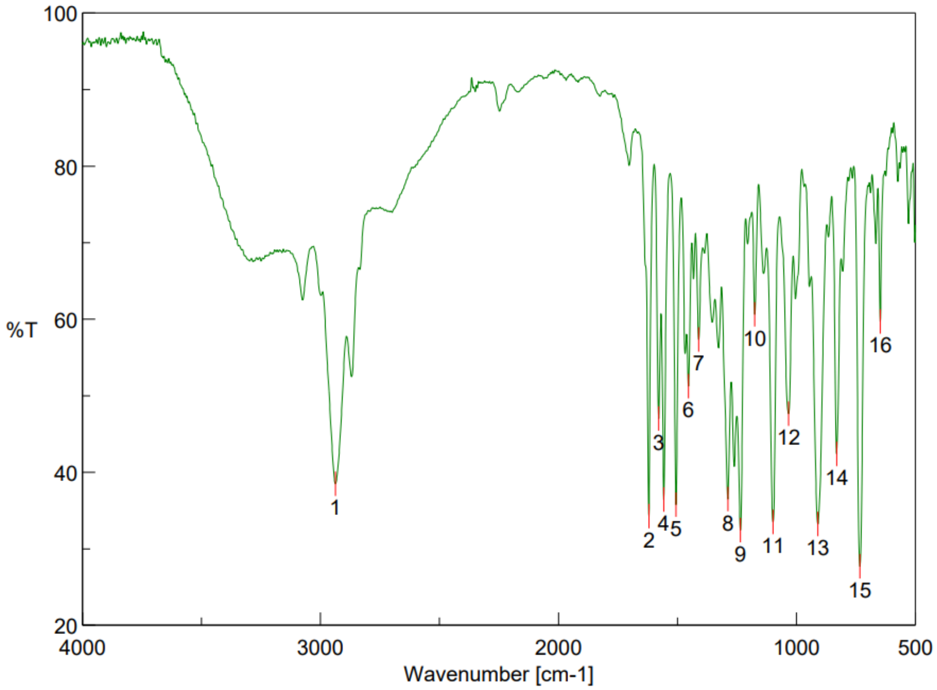

[ピーク検出結果]

No.	位置	強度	No.	位置	強度
1	2937.06	38.4798	2	1619.91	34.2404
3	1578.45	47.0115	4	1557.24	36.403
5	1506.13	35.7427	6	1454.06	51.221
7	1410.67	57.3193	8	1288.22	36.4719
9	1235.18	32.4103	10	1175.4	60.6207
11	1098.26	33.4858	12	1032.69	47.6284
13	910.236	33.2162	14	831.169	42.3782
15	732.817	27.7018	16	647.965	59.6958

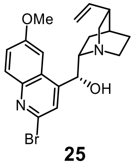

25

Supplementary Figure 50 IR (neat) spectra of **25**

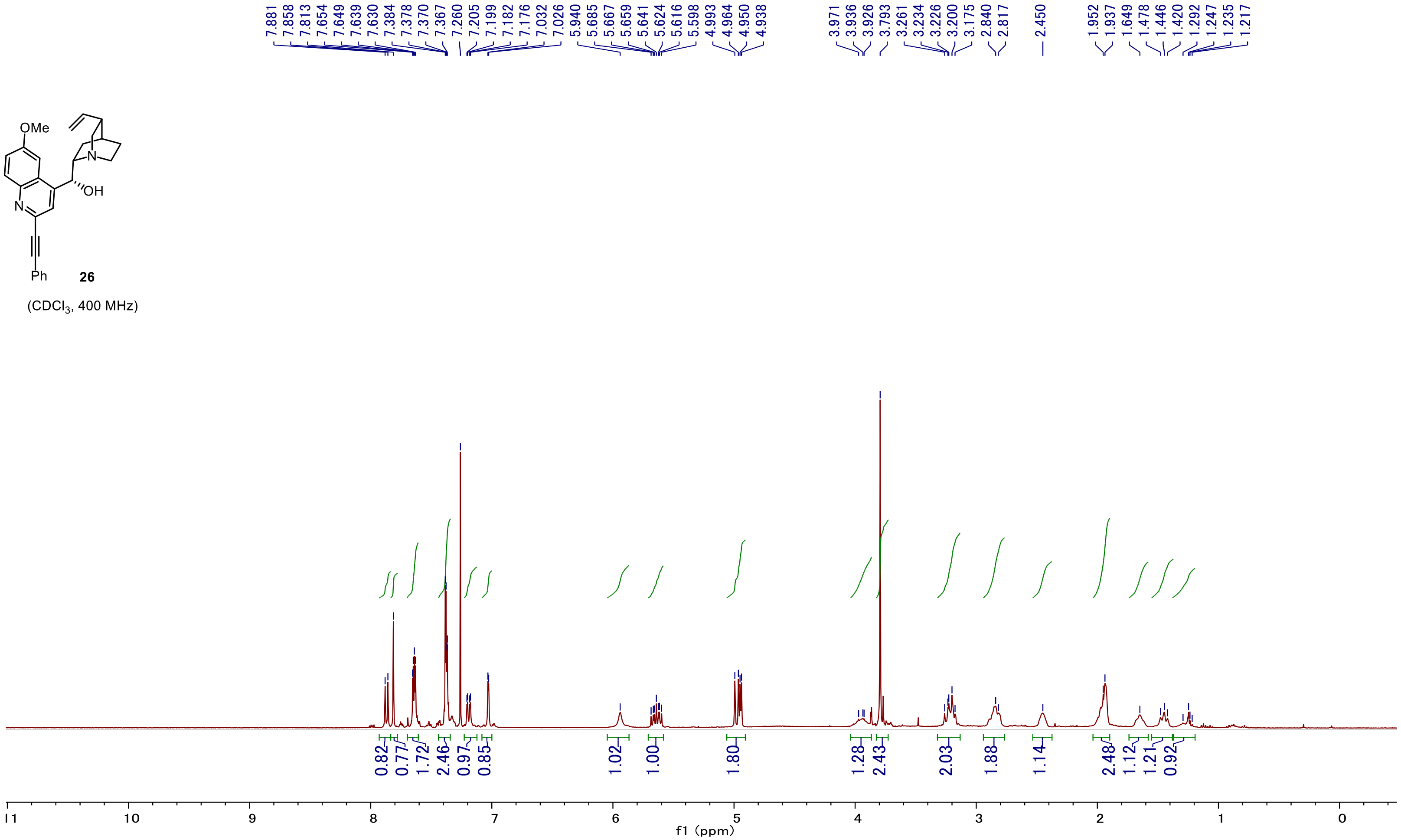

Supplementary Figure 51 ^{1}H NMR (400 MHz, $CDCl_3$) spectra of **26**

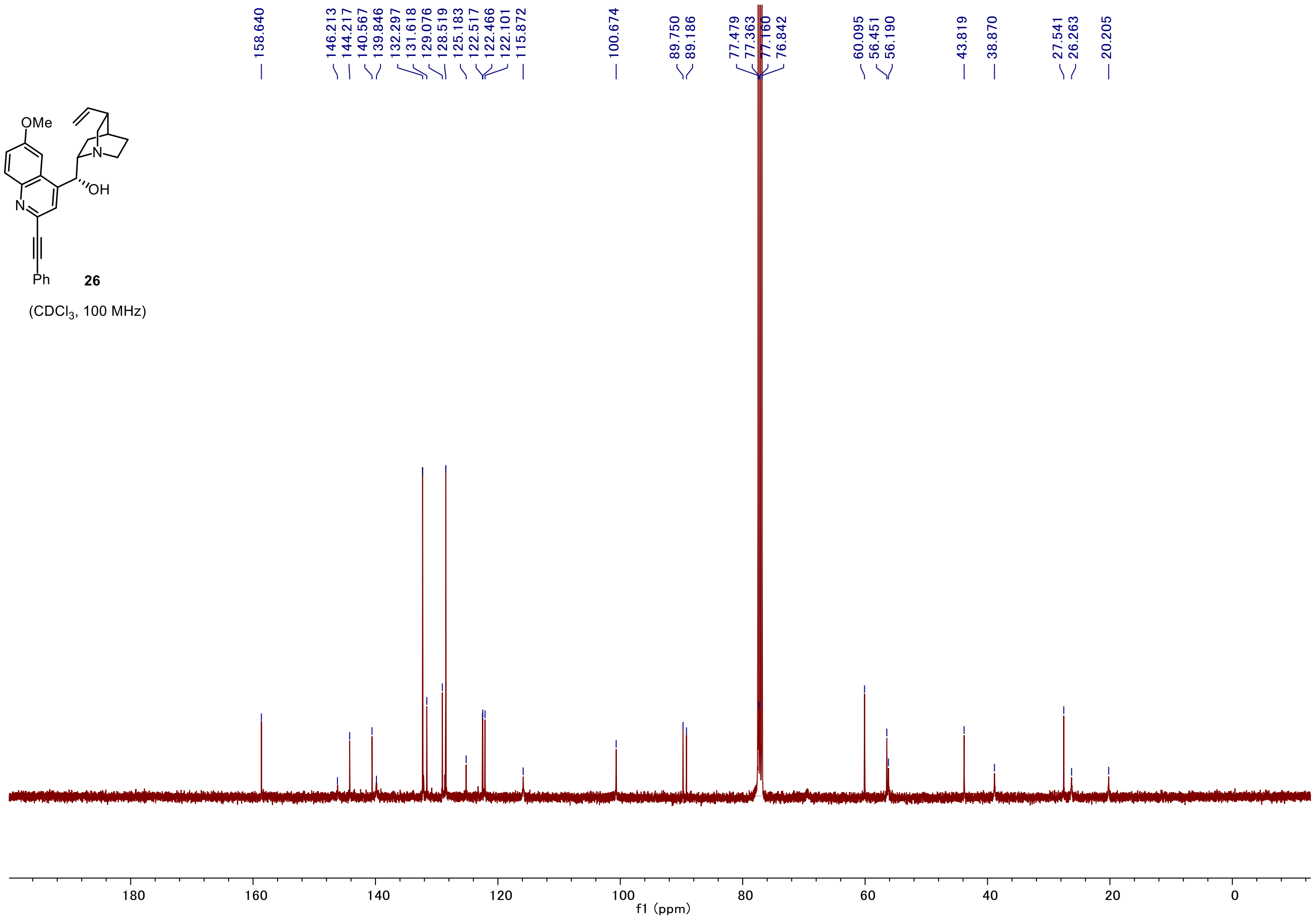

Supplementary Figure 52 ^{13}C NMR (100 MHz, $CDCl_3$) spectra of **26**

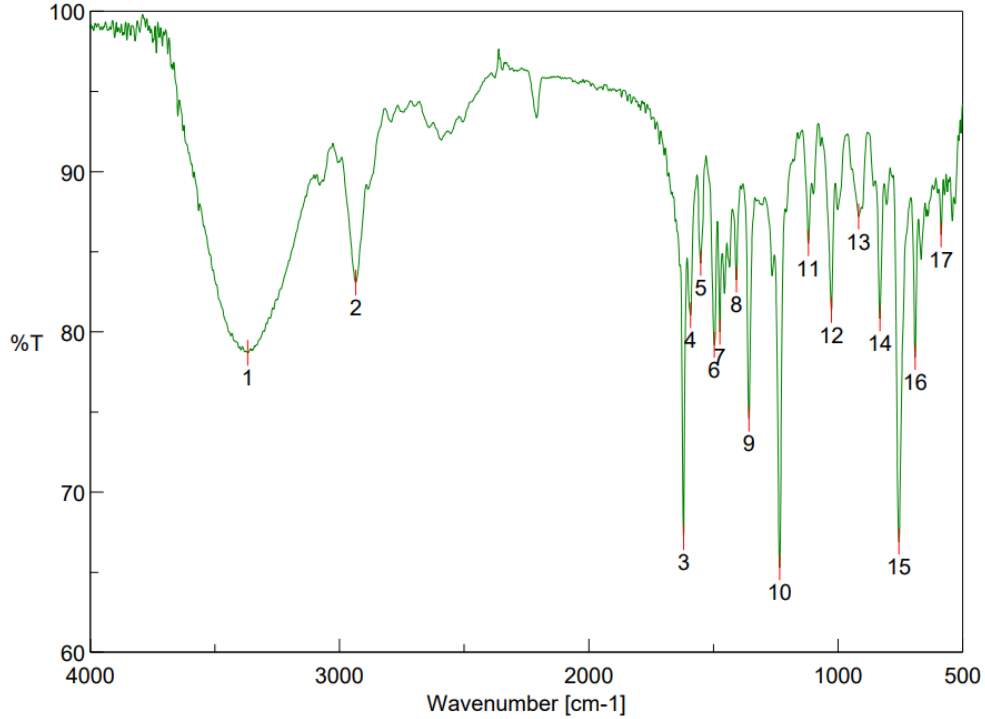

[ピーク検出結果]

No.	位置	強度	No.	位置	強度
1	3368.07	78.6754	2	2935.13	83.064
3	1619.91	67.1746	4	1591.95	81.0228
5	1551.45	84.2794	6	1496.49	79.1749
7	1474.31	79.9971	8	1407.78	83.2299
9	1357.64	74.5592	10	1234.22	65.2918
11	1118.51	85.516	12	1026.91	81.3484
13	916.986	87.1804	14	832.133	80.8169
15	755.959	66.8836	16	690.391	78.4051
17	586.254	86.0706			

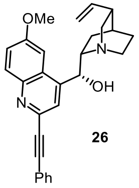

26

Supplementary Figure 53 IR (neat) spectra of **26**

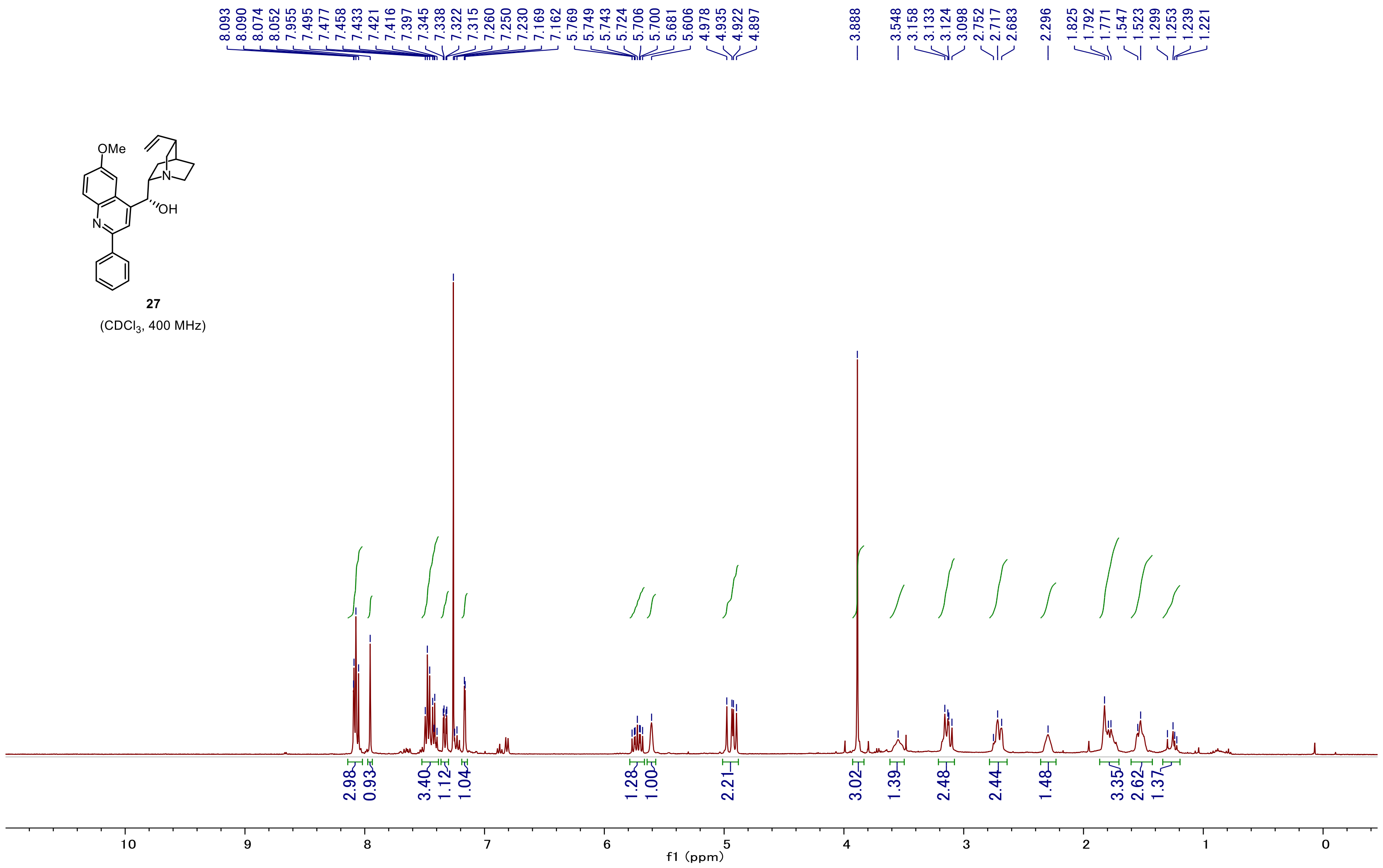

Supplementary Figure 54 ^{1}H NMR (400 MHz, $CDCl_3$) spectra of **27**

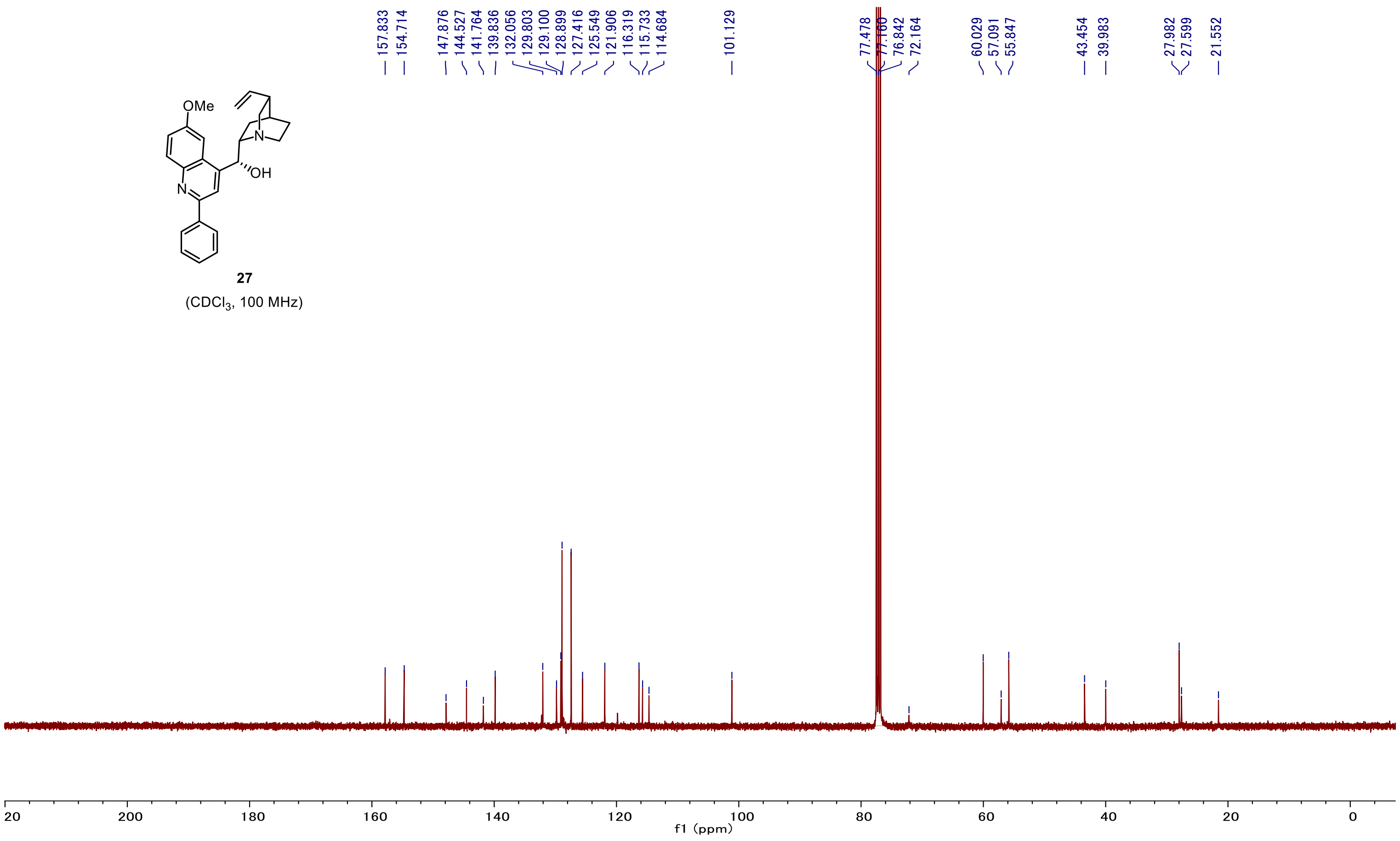

Supplementary Figure 55 ^{13}C NMR (100 MHz, $CDCl_3$) spectra of **27**

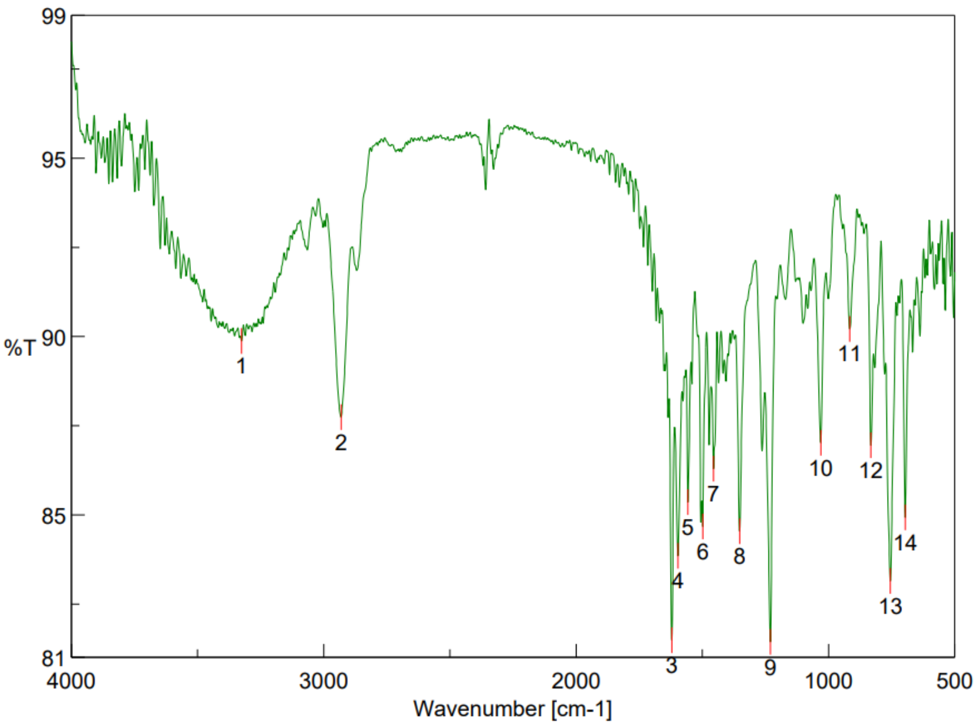

[ピーク検出結果]

No.	位置	強度	No.	位置	強度
1	3325.64	89.8696	2	2931.27	87.7344
3	1620.88	81.4799	4	1596.77	83.85
5	1557.24	85.3507	6	1498.42	84.6736
7	1455.99	86.2915	8	1351.86	84.5393
9	1230.36	81.4342	10	1030.77	87.013
11	916.022	90.2062	12	832.133	86.9483
13	754.995	83.1391	14	696.177	84.9235

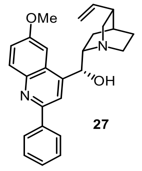

27

Supplementary Figure 56 IR (neat) spectra of **27**

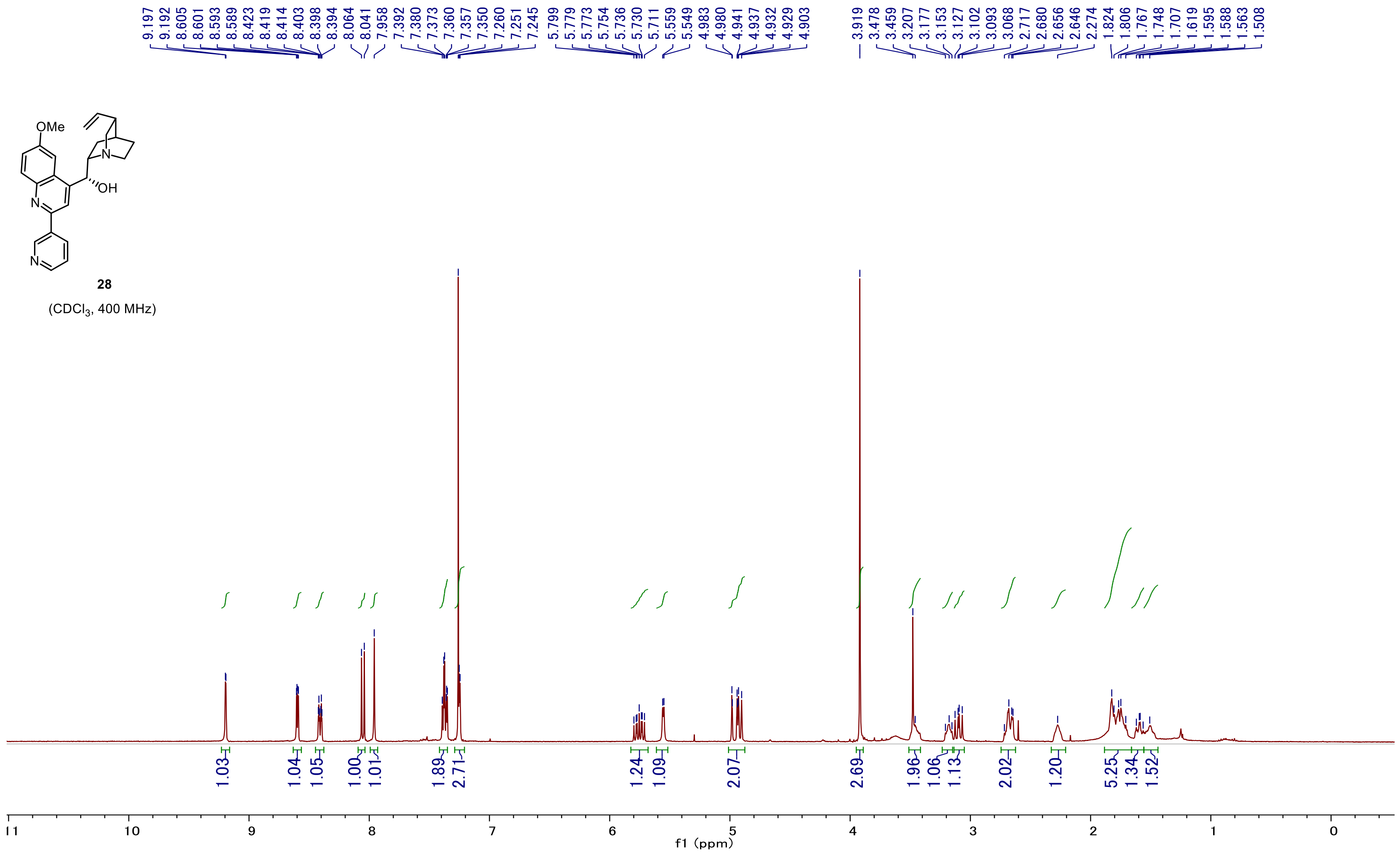

Supplementary Figure 57 ^{1}H NMR (400 MHz, $CDCl_3$) spectra of **28**

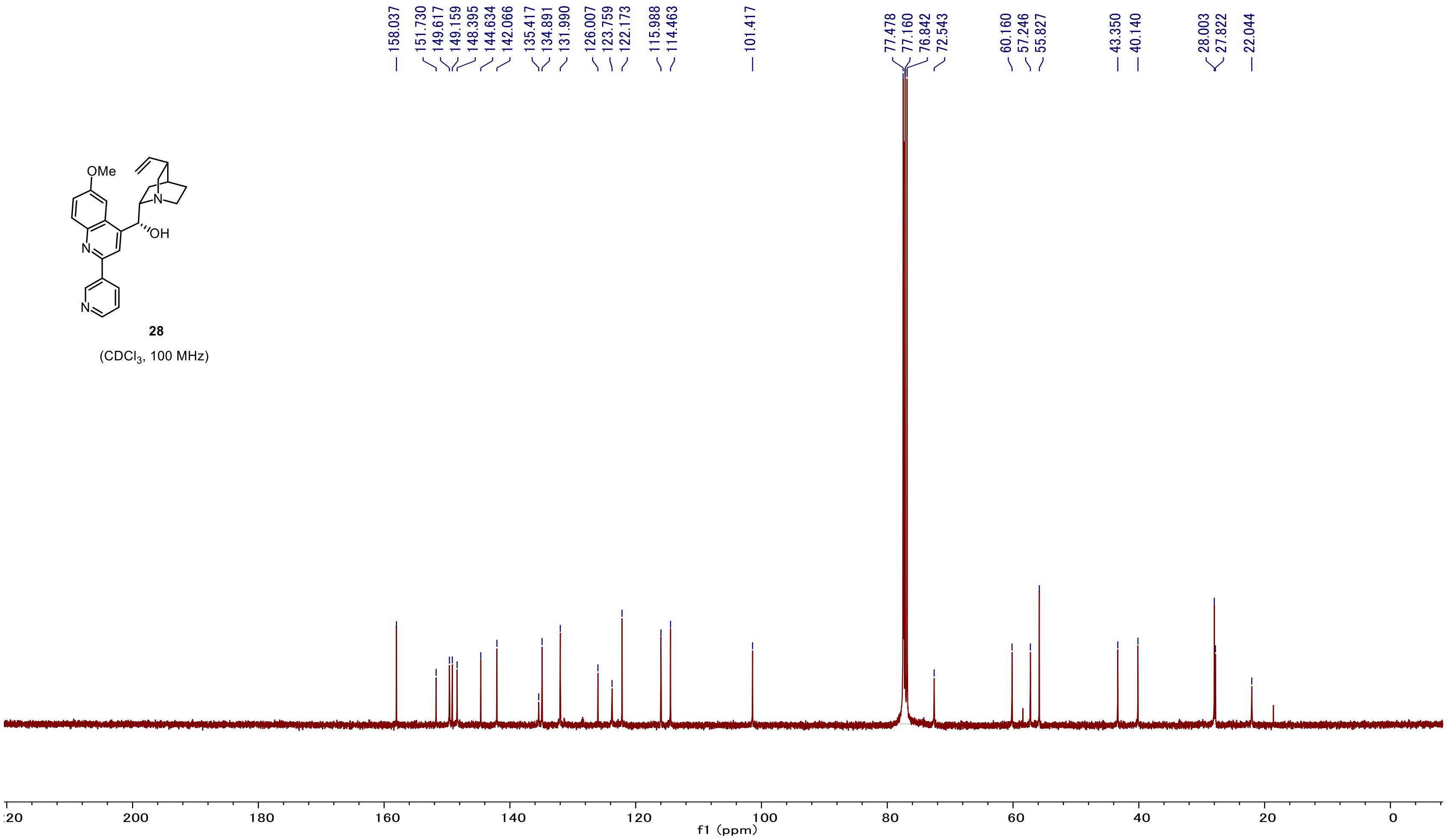

Supplementary Figure 58 ^{13}C NMR (100 MHz, $CDCl_3$) spectra of **28**

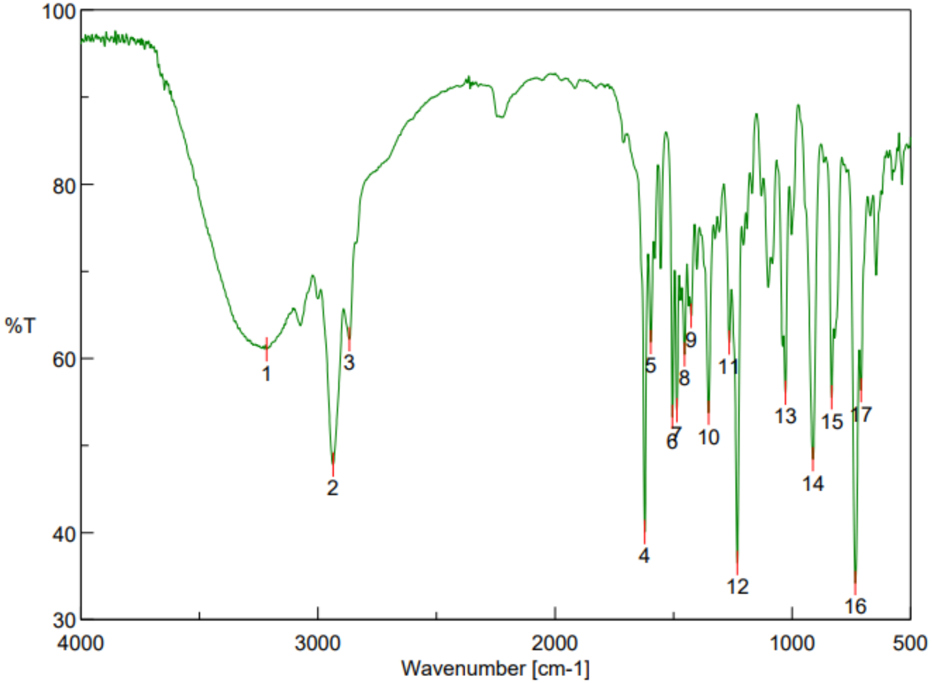

［ピーク検出結果］

No.	位置	強度	No.	位置	強度
1	3216.68	61.0335	2	2936.09	47.7908
3	2867.63	62.196	4	1621.84	40.0552
5	1595.81	61.876	6	1505.17	53.2646
7	1485.88	54.0425	8	1454.06	60.4482
9	1426.1	64.8562	10	1351.86	53.7492
11	1265.07	61.8132	12	1231.33	36.507
13	1027.87	56.0407	14	912.165	48.4176
15	833.098	55.529	16	732.817	34.2093
17	708.712	56.3387			

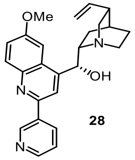

28

Supplementary Figure 59 IR (neat) spectra of **28**

2. Supplementary References

1. Blowers, J. W., Saxton, J. & Swanso, A. G. Approaches to the synthesis of aspidosperma alkaloids. Part II. The synthesis of 18,19-didehydrotabersonine. *Tetrahedron* **42**, 6071-6095 (1986).

2. Nagano, T. & Kinoshita, H. A New and Convenient Method for the Synthesis of Dehydroamino Acids Starting from Ethyl *N*-Boc- and *N-Z-α*-Tosylglycinates and Various Nitro Compounds. *Bull. Chem. Soc. Jpn.* **73**, 1605-1613 (2000).

3. Maloney, K. M., Nwakpuda, E., Kuethe, J. T. & Yin, J. One-Pot Iodination of Hydropyridines. *J. Org. Chem.* **74**, 5111-5114 (2009).

4. Kim, M. S., Choi, Y. M. & An, D. K. Lithium diisobutyl-*t*-butoxyaluminum hydride, a new and efficient reducing agent for the conversion of esters to aldehydes. *Tetrahedron Lett.* **48**, 5061-5064 (2007).

5. Liu, W., Qin, W., Wang, X., Xue, F., Liu, X.-Y. & Qin, Y. Bioinspired Synthesis of (+)-Cinchonidine Using Cascade Reactions. *Angew. Chem. Int. Ed.* **57**, 12299-12302 (2018).

6. Raheem, I. T., Goodman, S. N. & Jacobsen, E. N. Catalytic Asymmetric Total Syntheses of Quinine and Quinidine. *J. Am. Chem. Soc.* **126**, 707-706 (2004).

7. Wengryniuk, S. E., Weickgenannt, A., Reiher, C., Strotman, N., Chen K., Eastgate M. D. & Baran, P. S. Regioselective Bromination of Fused Hetereocyclic N-Oxides. *Org. Lett.* **15**, 792-795 (2013).
